# Circular RNAs in human diseases

**DOI:** 10.1002/mco2.699

**Published:** 2024-09-04

**Authors:** Yuanyong Wang, Jin Zhang, Yuchen Yang, Zhuofeng Liu, Sijia Sun, Rui Li, Hui Zhu, Tian Li, Jin Zheng, Jie Li, Litian Ma

**Affiliations:** ^1^ Department of Thoracic Surgery Tangdu Hospital Air Force Medical University Xi'an China; ^2^ Key Laboratory of Carcinogenesis and Translational Research (Ministry of Education) The First Department of Thoracic Surgery Peking University Cancer Hospital and Institute Peking University School of Oncology Beijing China; ^3^ Department of Traditional Chinese Medicine Tangdu Hospital Air Force Medical University Xi'an China; ^4^ Key Laboratory of Integrated Traditional Chinese and Western Medicine Tumor Diagnosis and Treatment in Shaanxi Province Xi'an China; ^5^ Department of Traditional Chinese Medicine The Third Affiliated Hospital of Xi'an Medical University Xi'an China; ^6^ Department of Epidemiology School of Public Health Air Force Medical University Xi'an China; ^7^ Department of Anatomy Medical College of Yan'an University Yan'an China; ^8^ Institute of Medical Research Northwestern Polytechnical University Xi'an China; ^9^ School of Basic Medicine Fourth Military Medical University Xi'an China; ^10^ Department of Endocrine Xijing 986 Hospital Air Force Medical University Xi'an China; ^11^ Department of Gastroenterology Tangdu Hospital Air Force Medical University Xi'an China; ^12^ School of Medicine Northwest University Xi'an China

**Keywords:** biomarker, cancers, circular RNAs, exosome, prognosis, progression

## Abstract

Circular RNAs (circRNAs) are a unique class of RNA molecules formed through back‐splicing rather than linear splicing. As an emerging field in molecular biology, circRNAs have garnered significant attention due to their distinct structure and potential functional implications. A comprehensive understanding of circRNAs’ functions and potential clinical applications remains elusive despite accumulating evidence of their involvement in disease pathogenesis. Recent research highlights their significant roles in various human diseases, but comprehensive reviews on their functions and applications remain scarce. This review provides an in‐depth examination of circRNAs, focusing first on their involvement in non‐neoplastic diseases such as respiratory, endocrine, metabolic, musculoskeletal, cardiovascular, and renal disorders. We then explore their roles in tumors, with particular emphasis on exosomal circular RNAs, which are crucial for cancer initiation, progression, and resistance to treatment. By detailing their biogenesis, functions, and impact on disease mechanisms, this review underscores the potential of circRNAs as diagnostic biomarkers and therapeutic targets. The review not only enhances our understanding of circRNAs’ roles in specific diseases and tumor types but also highlights their potential as novel diagnostic and therapeutic tools, thereby paving the way for future clinical investigations and potential therapeutic interventions.

## Graphical abstract

Circular RNAs (circRNAs) play significant roles in various human diseases, including respiratory, endocrine, metabolic, musculoskeletal, cardiovascular, renal diseases, and cancer. This review highlights their biogenesis, pathogenic potential, and potential applications as diagnostic biomarkers and therapeutic targets.

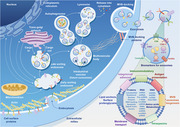

## 1 INTRODUCTION

Circular RNAs (circRNAs), initially considered splicing byproducts or degradation‐resistant intermediates, have gained recognition through advanced detection technologies for their diverse roles as noncoding RNAs containing hundreds to thousands of nucleotides.^1^ They form closed loops by linking 3′‐ and 5′‐terminals of linear RNAs, with biogenesis processes including reverse splicing across viral, eukaryotic, and prokaryotic genomes.^2^ Classified into intronic circRNAs (ciRNAs),^3^ exonic circRNAs (EcircRNAs),^4^ and exon–intron circRNAs (EIciRNAs),^5^ circRNAs play crucial roles in RNA processing and gene expression regulation, influencing various biological activities.^6^, ^7^, ^8^, ^9^, ^10^

Delving deeper into their implications beyond biogenesis, circRNAs exhibit significant involvement in non‐neoplastic diseases.^11^, ^12^ They are increasingly associated with respiratory diseases, metabolic disorders such as diabetes and obesity, musculoskeletal diseases like rheumatoid arthritis (RA), cardiovascular diseases (CVDs) including heart failure (HF) and atherosclerosis, and renal diseases including kidney injury and fibrosis. This broad spectrum highlights circRNAs as potential biomarkers and therapeutic targets across diverse medical conditions.

**FIGURE 1:**
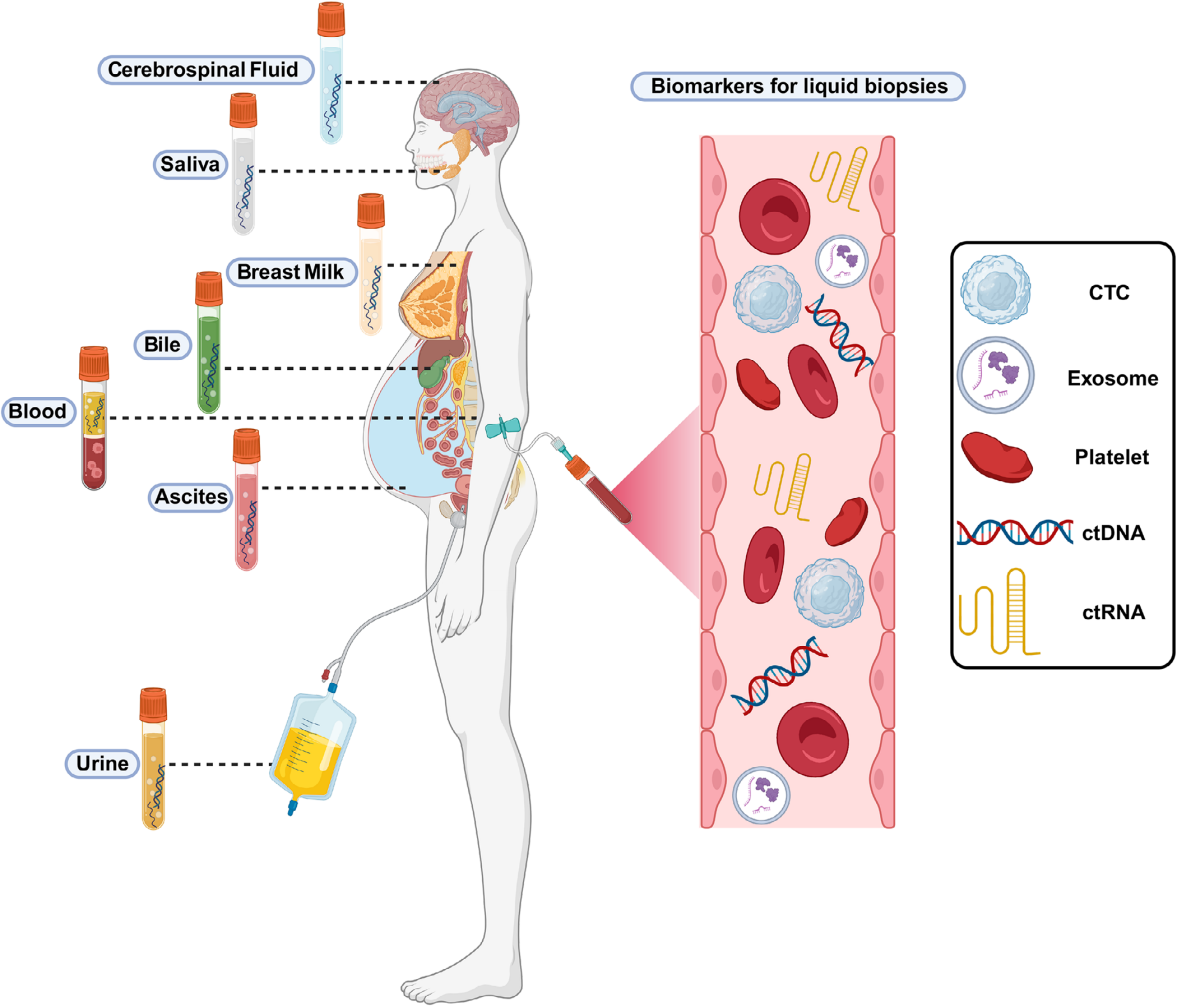
Liquid biopsy of tumors. This figure elucidates the concept and fundamental components of liquid biopsy. It delineates various bodily fluids as potential sources and underscores critical biomarkers such as ctDNA and ctRNA for the detection and monitoring of cancer. The diagram accentuates the interrelationship between bodily fluids and the human body, thereby illustrating the simplicity and convenience of liquid biopsy in the context of cancer diagnosis and surveillance. (ctDNA, circulating tumor DNA; ctRNA, circulating tumor RNA).

Transitioning to cancer, circRNAs emerge as pivotal players in tumorigenesis, tumor progression, and therapeutic resistance, addressing persistent challenges in cancer diagnosis and treatment.^13^, ^14^ Extracellular vesicles (EVs), particularly exosomes, are explored as carriers of circRNAs, offering insights into cancer biology^15^, ^16^ and resistance mechanisms^17^, ^18^, ^19^ (Figure 1). EVs, enriched with biomolecules like proteins, nucleic acids, and lipids, hold promise for revolutionizing cancer diagnostics and treatments through liquid biopsy approaches.^20^, ^21^, ^22^

In summary, this review comprehensively explores circRNAs’ multifaceted roles across human diseases, underscoring their potential in clinical diagnostics, prognostics, and therapies. By elucidating circRNA dysregulation mechanisms and their implications, this review aims to provide valuable insights into leveraging circRNAs for improved disease management and patient outcomes.

## 2 FUNCTION OF circRNAs

In this section, we explore the diverse functions of circRNAs, elucidating their pivotal roles in RNA processing and gene regulation. By examining their formation mechanisms and structural characteristics, we highlight how circRNAs serve as critical intermediates and regulators in cellular processes. This section aims to underscore the functional versatility of circRNAs and their implications across various biological contexts.

### 2.1 CircRNAs origin and activity

CircRNAs represent novel endogenous ncRNAs that contain covalently closed loop structures but without 3′‐polytails, 5′‐caps, or polyadenylated tails.^23^ CircRNAs regulate themselves independently of their linear transcripts due to their evolutionarily conserved structure and cell‐specific expression profiles.^24^, ^25^ As discovered by Li et al.^26^ through RNA‐seq, there are numerous exosomal circRNAs (exocircRNAs), and they are new, stable RNAs existing in exosomes. CircRNAs are suggested to exist in cell RNAs and migrate into exosomes; then, molecular information can be transferred to the recipient cells.^27^, ^28^

### 2.2 miRNA sponges

CircRNAs can function as competitive endogenous RNAs (ceRNAs), which are rich in binding sites for miRNAs (also referred to as miRNA response elements [MREs]), and they show competitive binding to miRNAs, thereby removing the inhibition against downstream targets, thus affecting the transduction of intracellular signals and the expression of target genes.^29^, ^30^ The function of circRNAs as miRNA sponges represents the classic model of their function.^31^, ^32^ For instance, the cerebellar degeneration‐related protein 1 antisense, or circRNA sponge for miRNA (miR)‐7 (ciRS‐7), as identified by Hansen et al.^33^ exhibited a negative regulatory effect on miR‐7 levels. Additionally, over 70 binding domains for miR‐7 were discovered in ciRS‐7, which exhibited high expression in HEK293 cells while binding as many as 20,000 miR‐7 molecules per cell. Moreover, circRNA–PVT1 silencing has been suggested to suppress sirtuin 7 by increasing miR‐3666 expression, eventually inhibiting hepatocellular carcinoma (HCC) cells in proliferation and metastasis.^34^ Furthermore, circRNAs can function as miRNA sponges in humans and in parasites including nematodes.^35^ Additionally, circRNAs that can sponge miRNAs have been detected in plants such as citrus,^36^ wheat,^37^ and Arabidopsis.^38^

### 2.3 Involvement in translation

Although circRNAs were once thought to be ncRNAs, they have recently been found to be associated with peptide/protein translation, refuting the widely accepted theory.^39^, ^40^, ^41^, ^42^ When circRNAs contain the internal ribosome entry site (IRES), eukaryotic ribosomes are thought to initiate translation from such circRNAs.^43^, ^44^ For instance, circ‐PINT is translated through the IRES into PINT87aa polypeptide, containing 87 amino acids. PINT87aa can interact with the polymerase‐associated factor complex gene, thereby inhibiting glioma occurrence.^45^ In circRNAs, their IRES domains are later estimated as the binding sites for numerous RNA‐binding proteins (RBPs), such as PTB and HUR, and they regulate IRES element‐mediated protein translation.^46^ Based on further research developments, circRNAs that do not contain IRES elements have been found to be translated into various functional proteins. For instance, N6‐methyladenosine (m6A) has a certain impact on mRNA translation in the case of heat shock stress and can modulate circRNAs translation.^42^, ^47^ This may be related to m^6^A modification via the methyltransferase like (METTL)3/METTL14–WTAP protein complex, FTO‐mediated m^6^A de‐modification, and the m^6^A modification‐containing site that initiates protein translation through recruiting YTHDF3 and subsequent eIF4G2, thus shedding more light on circRNA chemical modifications.^48^ In addition, circRNAs are found to utilize overlapping codons in protein translation, representing a unique translation approach.^49^ As discovered, circ‐AKT3 can produce the new functional protein AKT3‐174aa using overlapping codons. The latter can bind to p‐PDK1 to impact AKT2/3 phosphorylation, thereby exerting negative regulation on the PI3K/AKT pathway and suppressing brain tumor genesis and progression.^50^ This unique translation process is significant for investigating the novel activities of circRNAs.

### 2.4 Protein–circRNA interactions

To regulate protein levels, circRNAs function as protein sponges and can bind to RBPs to form RNA–protein complexes.^51^, ^52^ For instance, MBL, an RNA splicing factor, can bind to the parent gene at the second exon, thereby promoting its cyclization to form circ‐MBL in Drosophila.^52^ Meanwhile, many sites exist in circ‐MBL, which can bind to MBL protein and reduce MBL's effective content.^52^ Additionally, circRNAs interact with specific target proteins, playing roles in cellular division, proliferation, and apoptotic processes.^53^

For instance, circ‐Foxo3, in combination with cyclin‐dependent kinase 2 (CDK2) and cyclin‐dependent kinase inhibitor 1 (p21), forms the circ‐Foxo3–p21–CDK2 ternary complex, thereby inhibiting CDK2 activity and blocking the cell cycle. Furthermore, endogenous circ‐Foxo3 knockdown enhances cell proliferation.^54^ As discovered in another study, circ‐ANRIL binds with PES1 protein, which suppresses the binding to pre‐rRNA and the exonuclease‐regulated maturation of rRNA. Therefore, circANRIL decreases ribosomal biogenesis, thereby activating p53, subsequently increasing apoptosis, and decreasing proliferation.^55^ AKT and cardiomyocyte survival can both be improved by the upregulation of circAmotl1 in neonatal cardiac tissue. AKT1 and PDK1 are bound by circAmotl1, which activates nuclear translocation and phosphorylation of AKT.^56^ As proteins possess numerous activities, further investigations are needed to explore the protein‐binding activity of circRNAs.

### 2.5 Gene transcriptional regulation

**FIGURE 2:**
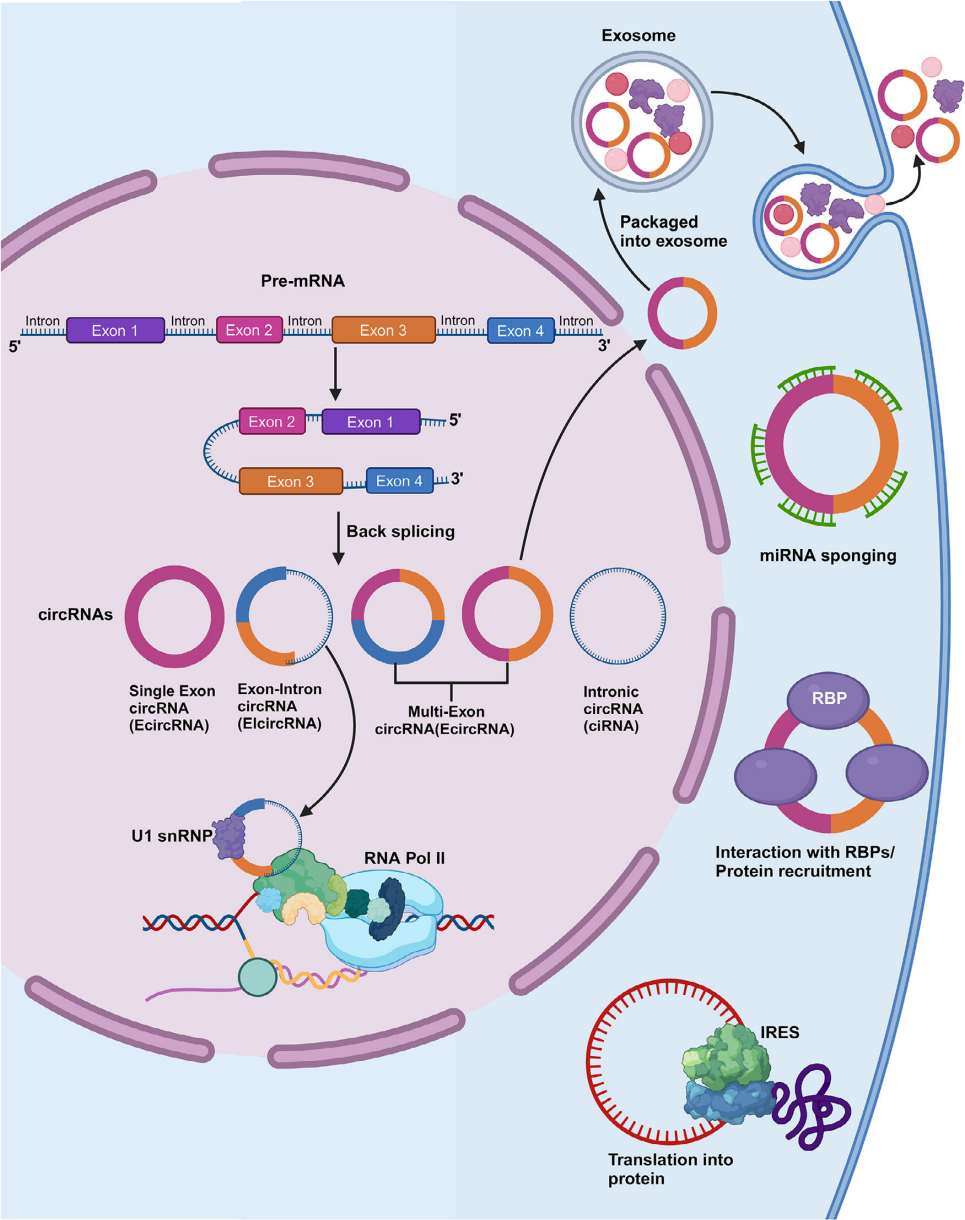
Major activities of circRNAs. This figure elucidates the multifaceted processes involved in eukaryotic gene expression, encompassing transcription within the nucleus and translation in the cytoplasm. It underscores critical stages such as pre‐mRNA splicing, miRNA sponging, interactions with RBPs, packaging into exosomes, and ultimately, protein translation. The illustration emphasizes the intricate interplay of molecular machineries and regulatory mechanisms that govern these processes. (RBPs, RNA binding proteins).

EIciRNAs and ciRNAs exhibit competitive regulation of parental gene transcription via linear splicing. CiRNAs act as active regulatory factors for RNA polymerase (Pol) II, which in turn can regulate parental gene transcription.^57^ EIciRNAs interact with U1 small nuclear RNA (snRNP) to form the EIciRNA–U1 snRNP complex, which further interacts with the RNA Pol II transcriptional complex, affecting parental gene expression.^5^, ^52^ Noncoding intron transcripts, like ci‐ankrd52, can positively regulate the transcription by RNA Pol II, and exhibit cis‐regulation of the parental coding genes; moreover, they are associated with the RNA Pol II extension mechanism.^3^ According to a 2020 study, circ‐DAB1 increases the recombinant signal‐binding protein level of the immunoglobulin kappa J region (RBPJ), thereby enhancing RBPJ's binding to the promoter of DAB adaptor protein 1 (DAB1) and promoting DAB1 transcription^58^ (Figure 2).

### 2.6 Exosomal‐circRNAs in cancer

Most types of cells release exosomes, which are small EVs originating from endocytosis.^59^, ^60^ Exosomes function as mediators of intercellular communication throughout tumorigenesis. Additionally, circRNAs can be loaded into exosomes to create exosomal‐circRNAs (exo‐circRNAs), which transport their cargo molecules horizontally to recipient cells, communicating with nearby or distant cells.^61^ Exo‐circRNAs modify biological signaling pathways, affecting the onset and progression of cancer. According to a recent study, circLPAR1 levels are reduced in colorectal cancer (CRC) tissues and are encapsulated in exosomes. Additionally, the level of circLPAR1 in plasma exosomes is considerably lower during CRC progression but reverts postoperatively.^62^ Currently, two theories may elucidate how exo‐circRNAs function: circRNA clearance and cell communication.^63^, ^64^ According to Huang et al.,^63^ exocircRNA‐100338 increases the metastatic capacity of HCC cells when delivered to recipient cells inside EVs. Conversely, since circRNAs are more abundant than linear forms in EVs, Lasda and Parker^64^ proposed that exosomes may use EVs to remove endogenous cellular circRNAs. Nevertheless, Alhasan et al.^65^ hypothesized that exosomal exonucleases are responsible for circRNA enrichment in exosomes. Exosomes are easily accessible and protect the RNAs in different human biofluids from degradation, irrespective of the mechanism involved. All of these discoveries suggest that exo‐circRNAs may be useful biological markers and treatment targets for human diseases.

### 2.7 CircRNA detection and characterization

Over the past few decades, researchers have identified over 100,000 distinct human circRNAs.^66^, ^67^ Nevertheless, due to their distinct circular morphology, low abundance, and absence of 5′‐caps and 3′‐poly (A) tails, circRNAs are challenging to accurately identify and detect using standard approaches.^68^

The initial identification and characterization of circRNAs involved using Northern blotting and PCR methods (Table 1).^69^ The most accurate method for validating circRNA is Northern blotting, which utilizes RNA probes for hybridization and denatured polyacrylamide gel electrophoresis to separate circRNAs into their linear counterparts.^3^, ^70^, ^71^ However, low‐abundance circRNAs are difficult to identify by Northern blotting, and there is no high‐throughput detection method available.^72^ The most popular technique for circRNA analysis is RT‐qPCR, which uses a series of primers to amplify circRNA fragments and detect circRNA at the position flanking the BSJ locus.^70^, ^73^ CircRNA molecules may yield false positive results in reverse transcription due to rolling circle replication and strand displacement.^74^

**TABLE 1:** Methods for detecting and quantifying circRNA.

Method	Sensitivity/accuracy	Throughput	Advantages and disadvantages	References
Northern blotting	Low	Low	Gold standard analysis of circRNA validation; insensitive to low expression of circRNA	^3^, ^69^, ^70^, ^71^, ^72^
RT‐qPCR	Medium‐high	Low‐medium	Provide quantitative data; false positive signal may be generated	^70^, ^73^, ^74^
RCA	High	Medium	Simple operation, no need for high‐precision temperature cycle and additional separation steps.	^75^, ^76^
NanoString Technologies nCounter assays	High	Medium‐high	Quantify circRNA; requires special equipment and is expensive	^77^
Microarrays	Medium	High	High throughput and high detection efficiency; difficult to compare between different studies	^69^, ^78^, ^79^
FISH	High	Low	Forming stable DNA‐RNA hybrid; highly affected by personnel operations and instrumentation and costly	^80^, ^81^
RNA‐seq	Medium‐high	High	Widely used in the discovery of novel circRNAs; overlap with linear molecular signals, data processing capabilities are required	^82^, ^83^, ^84^, ^85^

Abbreviations: FISH, fluorescence in situ hybridization; RCA, rolling circle amplification; RNA‐seq, RNA‐sequencing; RT‐qPCR, reverse transcription quantitative real‐time PCR.

The circular morphology of circRNAs makes for a perfect template for the process of rolling circle amplification (RCA), making rolling RCA, a recently established technology, highly appropriate for circRNA analysis.^75^ The benefits of the reverse transcription RCA approach are ease of use and low cost while selectively amplifying target circRNAs.^76^ CircRNA detection using enzymes eliminates the requirement for primers. A DNA/circRNA hybrid is digested by duplex‐specific nucleases, which release a fluorescent probe fragment and circRNA that enhances the fluorescence signal when exposed to target circRNA.^86^ The most recent technique for precise and sensitive circRNA detection is the nCounter assay from NanoString Technologies. Without enzymatic reactions or bias, NanoString correctly quantifies circRNA using dual‐probe hybridization containing biotinylated capture probes as well as distinctive color‐coded reporter probes.^77^ Nonetheless, this approach necessitates expensive specialized equipment. Microarrays are a popular high‐throughput assay with strong sensitivity and accuracy for circRNA identification. These tests are appropriate for circRNA expression research since they are unaffected by lower transcript expression levels.^78^, ^79^ Nevertheless, it might be challenging to compare microarray data from different investigations.^69^ Fluorescence in situ hybridization is a popular method for studying the subcellular localization of RNA, using fluorescently labeled DNA probes.^80^, ^81^ However, this method takes a lot of time and costly signal‐detecting apparatus.^69^

The discovery of circRNAs and advances in biological research have both been considerably accelerated by the advent of RNA‐seq technology.^82^, ^83^ Before short‐read deep sequencing, circRNAs had to be biochemically enriched due to their low abundance in vivo and the absence of poly(A) tails.^68^ RNase R treatment significantly enriches circRNAs in samples.^73^, ^87^ For preliminary circRNA analysis, rRNA depletion is often utilized instead of poly(A) screening and transcriptomic sequencing before sequencing and building circRNA libraries.^84^ Numerous circRNAs have been discovered using ribo‐RNA sequencing, which also provides data on the expression profiles of coding and noncoding RNAs.^88^ Ribo‐RNA‐seq allows for direct quantitative comparison of the expression levels of circRNAs and their cognate linear RNAs.^89^ However, this method results in overlapping circRNA and linear RNA signals that cannot be distinguished in exonic areas.^85^ Numerous human genes that produce circRNA isoforms have been identified using the poly(A)‐RNA‐seq method, which is suitable for circRNAs lacking poly(A) tails.^90^ Small circRNAs might not be detected by this method, and it may also struggle to accurately detect and quantify unusual circular isoforms.^90^ Compared with ribo‐ or poly(A)‐RNA‐seq, the RNase R RNA‐seq approach may detect a greater number of individual circRNAs, thereby maximizing circRNA enrichment.^70^, ^71^ A straightforward, accurate, sensitive, and efficient technique for the analysis and detection of circRNA is still needed.

### 2.8 Application of CircRNAs

CircRNAs have attracted more attention due to their distinctive structure, immunogenicity, stability, and potential biological benefits. CircRNAs represent a stable alternative to mRNA‐based treatments and may serve as a complement using adeno‐associated vector technology.^91^, ^92^ Nanoformulations of circRNA delivery containing IRES have demonstrated a prolonged translational period.^91^, ^93^ The SARS‐CoV‐2 circRNA vaccine demonstrated long‐lasting antigen synthesis and the ability to generate neutralizing antibodies.^94^ The team from Peking University, led by Prof. Wei, developed a circRNA vaccine for COVID‐19's Delta variant for the first time. The vaccine provided broad‐spectrum defense against several novel coronavirus strains.^94^ However, developing methods to enhance circRNA synthesis and transport, and to attenuate the cellular immune response caused by circRNA, remains a challenge and requires further investigation and improvement.^95^, ^96^

Numerous diseases, including autoimmune disorders, cancer, liver disorders, neurological disorders,^97^, ^98^, ^99^ cardiovascular disorders, and diabetes, are accompanied by dysregulated circRNA expression.^85^, ^100^, ^101^, ^102^, ^103^ Numerous DNA, RNA, and protein‐based biological markers are currently used in clinical settings as tools for disease diagnosis.^104^ CircRNAs are excellent candidates for biological markers, as they are not readily broken down by nucleic acid exonucleases and have a significantly longer half‐life compared with linear RNAs.^105^ CircRNAs are abundantly expressed in the urine, saliva, gastric juice, and blood of prostate cancer patients, suggesting they could serve as biological markers for early cancer detection.^66^, ^106^, ^107^, ^108^, ^109^ Prostate cancer diagnosis was significantly enhanced in clinical studies by combining circRNA detection with prostate‐specific antigen testing.^110^

CircRNAs have also been identified as potential clinical treatment targets for a variety of diseases. F‐circRNA, for instance, promotes cellular survival, transformation, and therapy resistance in acute promyelocytic leukemia, thus representing a potential therapeutic target for disease management.^111^ CircSLC8A1 is abundantly present in the heart, and its suppression in vivo alleviates myocardial ischemia–reperfusion (I/R) injury and cardiac hypertrophy caused by stress overload.^112^, ^113^ CircRNA treatments are still in the early stages of development, and numerous challenges still need to be addressed. Designing and optimizing circRNA overexpression vectors, enhancing cyclization, creating effective delivery methods, improving chemical production processes, and controlling process development are significant challenges.^84^ Although much remains to be learned about circRNAs, they are anticipated to become a powerful tool for clinical diagnosis and treatment in the near future.

## 3 CircRNAs IN NONCANCER DISEASES

This section delves into the involvement of circRNAs in non‐neoplastic diseases, ranging from respiratory and metabolic disorders to musculoskeletal and cardiovascular conditions. By exploring specific examples and underlying mechanisms, we illuminate how dysregulated circRNAs contribute to disease pathogenesis and their potential as diagnostic biomarkers and therapeutic targets. This discussion aims to provide a comprehensive understanding of circRNAs’ roles beyond cancer biology.

### 3.1 CircRNAs in respiratory diseases

#### 3.1.1 CircRNAs in COVID‐19

COVID‐19 research on differentially expressed circRNAs (DEcircRNAs) is sparse, but holds promise for shedding light on DEcircRNAs post‐SARS‐CoV‐2 infection and their distribution across various samples. A genome‐wide dynamic analysis identified over 5000 circRNAs at diverse genomic locations in human lung epithelial cells infected with SARS‐CoV‐2.^114^ In addition, DEcircRNAs were detected in whole‐blood samples from recurrent COVID‐19 cases compared with healthy controls.^115^ Infection with SARS‐CoV‐2 may disrupt the expression of host circRNAs in the blood. A distinct expression profile of circRNAs has been observed in cerebrospinal fluid among COVID‐19 cases, healthy controls, and neurological disease cases.^116^ It is essential to investigate potential overlaps or differences in DEcircRNA types between neural cells and blood cells, given the tissue‐ and cell‐specific expression of circRNAs. Using techniques such as single‐cell RNA sequencing and spatial transcriptomics sequencing, we could gain a deeper understanding of SARS‐CoV‐2's systemic effects.

##### Possible mechanisms of circRNAs underlying SARS‐COV‐2 infection

**FIGURE 3:**
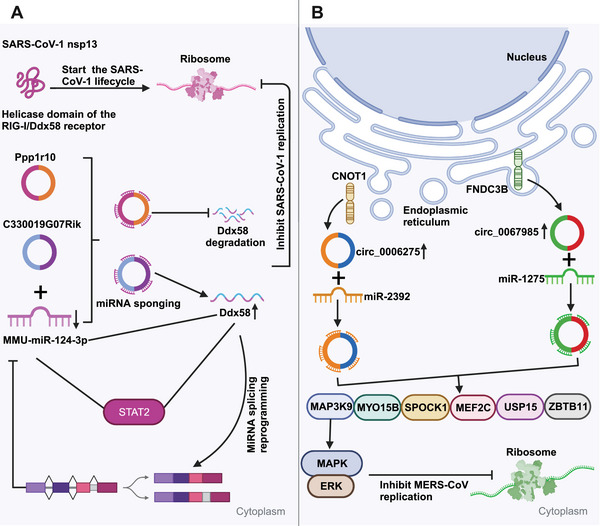
Regulation of viral replication by RIG‐I/Ddx58 and circRNA‐mediated miRNA sponging. (A) RIG‐I/Ddx58 receptor interacts with SARS‐CoV‐1 nsp13, initiating viral replication. miR‐124‐3p degrades Ddx58, reducing replication. CircRNAs Ppp1r10 and C330019G07Rik act as miR‐124‐3p sponges, preserving Ddx58. (B) circRNAs hsa_circ_0067985 and hsa_circ_0006275 are upregulated in MERS‐CoV infection, sponging hsa‐miR‐1275 and hsa‐miR‐2392, respectively, and regulating key viral replication targets. These circRNAs may be potential antiviral targets.

*CircRNAs sponge miRNAs to influence viral replication*. CircRNAs, which show a high evolutionary conservation degree, exhibit tissue‐ and cell‐specific expression patterns.^117^, ^118^, ^119^ CircRNAs containing miRNA‐responsive elements are the vital components of ceRNA network, which can modulate the expression of downstream target genes through sponging microRNAs (miRNAs), swiftly binding to specific miRNAs, and alleviating their inhibition against messenger RNAs (mRNAs) translation. Clearly, circRNAs containing miRNA‐responsive elements can effectively sponge miRNAs in the ceRNA network in order to impede the miRNA‐dependent target gene modulation.^33^, ^120^ CircRNAs are verified to have higher sponging activities than long noncoding RNA (lncRNA) transcripts and linear miRNAs.^70^, ^121^ Arora et al.^122^ recently detected the ceRNA network within SARS‐CoV‐1‐infected cells, which comprised a lncRNA (Gm26917), a miRNA (MMU‐miR‐124‐3p), a mRNA (Ddx58), a transcription factor (TF) (Stat2), together with two circRNAs (C330019G07Rik, Ppp1r10). The RIG‐I/Ddx58 receptor in this ceRNA network interacts with SARS‐CoV‐1 nsp13, which initiates the viral life cycle (Figure 3A). Ddx58 also participates in mRNA splicing and miRNA biosynthesis. The increased expression can result in miRNA splicing reprogramming, which decreased miRNA expression. miR‐124‐3p upregulation induces Ddx58 degradation, leading to decreased viral replication. Moreover, miR‐124‐3p can regulate Toll‐like receptor (TLR)‐dependent innate immunity via Stat3, therefore downregulating TNF‐α and IL‐6.^123^ The above findings indicate a potential similar role of miR‐124‐3p in modulating Stat2, impacting viral life cycle. In the ceRNA network, two circRNAs (C330019G07Rik and Ppp1r10) execute crucial modulating functions as the miR‐124‐3p sponges to inhibit Ddx58 degradation, which can affect SARS‐CoV‐1 replication. Additionally, as observed by Zhang et al.,^124^ host circRNAs mainly served as the miRNA sponges to influence MERS‐CoV replication. From Figure 3B, hsa_circ_0067985 originates in FNDC3B gene, which sponges hsa‐miR‐1275, while hsa_circ_0006275 originates in CNOT1 gene and sponges hsa‐miR‐2392. These two circRNAs show significant upregulation during MERS‐CoV infection, thereby regulating the expression of key downstream target genes including MYO15B, MAP3K9, MEF2C, SPOCK1, ZBTB11, and USP15. Collectively, the findings provide novel insights into the circRNA regulation and associated signaling pathways, presenting potential host‐targeted antiviral measures to resist SARS‐CoV‐2 infection.

*CircRNAs affect cytokines to regulate the immunity against SARS‐CoV‐2 infection*. CircRNAs exert a vital role in preventing viral damage by regulating the immunity in host–virus interaction.^125^ According to Li et al.,^126^ NF90/NF110, originating in human interleukin‐enhanced binding factor 3 (ILF3), made a direct impact on governing back‐splicing and collaboration with circRNA generation during viral infection. In the case of viral invasion, NF90/NF110 translocates into nucleus, which partly contributes to a reduction in circRNA production. The accumulation of NF90/NF110–circRNP complexes in the cytoplasm probably affects the host immunity. Based on the obtained results, circRNAs compete against viral mRNAs to bind to NF90/NF110, which potentially serve as the NF90/NF110 molecular reservoir to achieve rapid immunity during viral infection.^126^, ^127^ According to Chen et al.,^128^ self‐splicing introns‐derived circRNAs could bind to retinoic acid‐inducible gene I (RIG‐I) receptor to efficiently activate immune signaling during viral infection. It has been recently suggested that hsa_circ_0000479 upregulation among COVID‐19 patients modulates RIG‐I and IL‐6 levels through sequestering hsa‐miR‐149‐5p.^129^ Furthermore, circRNAs regulate immunity by mediating signaling pathways and cytokine production. Recently, an integrative analysis of protein transcription demonstrates significant modulation of hypoxia‐inducible factor‐1 (HIF‐1), epidermal growth factor receptor (ErbB), tumor necrosis factor (TNF), and mammalian target of rapamycin (mTOR) signaling pathways through SARS‐CoV‐2 infection.^130^ For DEcircRNAs, their parental genes associated with the above pathways are involved in various antiviral signaling pathways, including chemokines, interferon (IFN), RIG‐I‐like receptors, and mitogen‐activated protein kinases (MAPKs).^117^ Therefore, circRNAs can regulate cellular signaling and immuno‐inflammatory responses in SARS‐CoV‐2 infection.

Small molecular polypeptides, cytokines and glycoproteins produced via various cells, exert vital effects on numerous physiological events, including immuno‐inflammatory response regulation. Acting as immunomodulators, cytokines are related to endocrine, paracrine, and autocrine signaling, contributing to the immune response against viral infections.^131^, ^132^ Viral infections can induce cytokine production, which is of great importance for immune response control, antiviral defense, and target cell capacity to support viral replication.^133^ The infection with SARS‐CoV‐2 induces auto‐immunity by activating specific immune factors, including Th1 chemokines CXCL9/10/11, interferon‐inducible proteins, and 2′−5′ oligoadenylate synthase (OAS1‐3).^134^ Additionally, it decreases ribosomal protein transcription.^134^ OAS activation necessitates the participation of viral genome dsRNA, leading to 2′−5′ oligoadenylate (2′−5′A) generation. Then, 2′−5′A markedly enhances RNase L activity to execute the antiviral activity, thereby degrading viral RNA whereas impeding viral protein production.^135^ As discovered by Liu et al.,^100^ dsRNA‐activated protein kinase R (PKR) inhibitors are generated by endogenous circRNAs (16–26 bp) duplexes. In addition, circRNAs undergo global degradation via endonuclease RNase L, activating PKR antiviral pathway.

#### 3.1.2 CircRNAs in asthma

##### CircRNAs and airway smooth muscle cells

In general, mild asthma can be efficiently treated, while severe asthma will bring significant burdens on the economy and society. It usually entails notable alterations of airway smooth muscle cells (ASMCs), including hypertrophy, hyperplasia, as well as changes of fibrosis‐ and inflammation‐associated proteins.^136^ These aberrations in ASMCs make significant contributions to airway remodeling in severe asthma.^137^ Regrettably, there still lack such strategies for addressing such changes in ASMCs. Hence, it is imperative to thoroughly understand the asthma pathogenesis to develop efficient ways for mitigating the influence on ASMCs caused by severe asthma and improve patient prognosis.

The development of next‐generation sequencing has made it possible to identify many circRNAs in different types of cells and species.^88^ In asthma, there are few studies investigating the effect of circRNAs on ASMC dysfunction. The role of circRNAs in regulating the biological and cellular processes of ASMCs during asthma has recently been revealed in numerous studies. These processes, such as growth, invasion, apoptosis, extracellular matrix (ECM) generation, and inflammatory factor production, are primarily mediated through their role as ceRNAs for miRNAs, thereby regulating target gene mRNAs.

CircERBB2, originating at ERBB2 locus, is a notable circRNA in this context and is correlated with tumor genesis. CircERBB2 upregulation can be detected within bronchial biopsy tissues in patients with asthma and ASMCs treated with platelet‐derived growth factor BB (PDGF‐BB), the in vitro model of asthma.^138^ PDGF‐BB has been the factor implicated in inflammation and airway remodeling during asthma,^139^ which can stimulate ASMCs growth and invasion.^140^, ^141^ Noteworthily, PDGF‐BB upregulation is closely related to asthma severity, with increased ASMC proliferation and invasion being observed in asthma patients.^142^ CircERBB2 silencing with small hairpin RNA (shRNA) significantly alleviates the proliferation, invasion and inflammation of ASMCs caused by PDGF‐BB.^138^ From the mechanism perspective, circERBB2 can sponge miR‐98‐5p to regulate the expression of insulin‐like growth factor 1 receptor (IGF1R) and facilitate ASMC growth and invasion after PDGF‐BB administration.^138^

Another circRNA of interest, circHIPK3, also called circ_0000284, shows upregulation in ASMCs upon PDGF‐BB stimulation.^143^, ^144^ CircHIPK3 can enhance the growth and invasion of PDGF‐BB‐treated ASMCs and inhibit their apoptosis by interacting with miR‐326 for regulating stromal interaction molecule 1 (STIM1).^143^ STIM1 is an endoplasmic reticulum membrane protein exerting a role of the calcium sensor as well as the stimulatory factor for airway hyperresponsiveness (AHR) and ASMC remodeling during asthma.^145^, ^146^ In addition, circHIPK3 enhances ASMC growth, migration, and invasion after PDGF administration, which is achieved by regulating miR‐375/matrix metalloproteinase‐16 (MMP‐16) axis.^144^

Circ_0002594 and circ_CSNK1E are upregulated in PDGF‐stimulated ASMCs, and circ_0000029 exhibits decreased expression.^147^, ^148^, ^149^ Both circ_0002594 and circ_CSNK1E are significantly upregulated in samples collected from asthma patients.^147^, ^148^ Functional research shows that shRNA‐induced circ_CSNK1E silencing significantly decreases ASMC proliferation and migration after PDGF treatment.^147^ Based on computational research and experimental verification, circ_CSNK1E can regulate vesicle‐associated membrane protein 2 through binding to miR‐34a‐5p, therefore promoting the PDGF‐mediated ASMC growth and invasion.^147^ Besides, circ_0002594 can promote ASMC growth, invasion, and inflammation after PDGF administration. Moreover, circ_0002594 can sponge miR‐139‐5p to regulate tripartite motif 8 (TRIM8) expression, finally triggering cellular injury.^148^ In some studies, miR‐139‐5p administration and TRIM8 suppression decrease ASMC growth through suppressing the chromatin remodeling factor BRG1 and inhibiting NF‐κB pathway.^150^ Circ_0000029 can modulate ASMC growth and invasion by regulating KCNA1 and miR‐576‐5p expression.^149^ Combined with the above‐mentioned results, circRNAs are vital for regulating crucial activities of asthmatic ASMCs by interacting with miRNAs and target genes. Nevertheless, the complete circRNA activity scope and their interplay with additional mechanisms within ASMCs during asthma remain unclear. More investigations are needed to comprehensively investigate the impacts of circRNAs on asthmatic ASMCs.

##### CircRNAs and airway and bronchial epithelial cells

Airway epithelial cells play a crucial role as the first‐line protecting barrier, safeguarding both airway and lungs from antigens and inflammatory stimuli. Airway and bronchial epithelial cell impairments have been identified as a common feature of asthma. Abnormal circRNAs expression can significantly impact the functions of airway and bronchial epithelial cells.

In a comprehensive profiling study, Jia et al.^151^ utilized microarrays to investigate circRNAs within PM2.5‐exposed human bronchial epithelial cells (BEAS‐2B), and PM2.5 was the respiratory risk associated with pulmonary disorders like asthma. Circ_406961 is one of the dysregulated circRNAs, exhibiting the notable difference in fold change. Experiments indicate that circ_406961 is dose‐dependently downregulated within PM2.5‐exposed BEAS‐2B cells. Circ_406961 knockdown with siRNA aggravates inflammation and decreases cell viability, while its upregulation exerts the opposite effects. Mechanism research suggests that circ_406961 is vital for regulating inflammation through interaction with ILF2 and activation of STAT3/JNK pathways.

Moreover, circVPS33A is upregulated in plasma samples collected from plasma patients and in BEAS‐2B cells stimulated by house dust mites (HDM) protein (Der p1).^152^ Der p1 stimulation reduces cell viability, growth, invasion, migration, and promotes autophagy, inflammation, and apoptosis of BEAS‐2B cells. CircVPS33A knockdown with siRNA can mitigate the Der p1‐induced cellular injuries. Mechanically, circVPS33A can improve the above cellular injuries through sponging miR‐192‐5p, thus regulating HMGB1 expression. CircARRDC3 can promote mucus generation and aggravate inflammation of IL‐13‐treated nasal epithelial cells during allergic rhinitis, serving as the prominent risk factor related to asthma progression.^153^ Its regulation can be achieved via miR‐375/KLF4 axis. Collectively, the above results suggest the contribution of dysregulated circRNAs to asthma‐related dysfunction in bronchial epithelial cells. Nevertheless, the above results need to be validated by clinical studies and in vivo animal models.

##### CircRNAs and airway goblet cells

Airway goblet cells are crucial for keeping airway homeostasis by producing mucin.^154^ Goblet cells can differentiate from airway epithelial cells called club (Clara) cells upon stimulation with growth factors, genetic factors, environmental insults, and inflammation.^154^, ^155^ The out‐of‐control proliferation of goblet cells promote mucin generation in the airway.^156^ Excess mucin accumulation can be frequently observed in different airway disorders, like asthma, cystic fibrosis, and chronic obstructive pulmonary disease (COPD).^156^ Hyperplasia of goblet cells (the higher goblet cell count) in asthma is an important airway remodeling feature, which is correlated with chronic excessive mucus production and the resulting airway obstruction.^157^ CircRNAs can induce hyperplasia of goblet cells. For example, Wang et al.^158^ suggested that circZNF652, also called circ_0000782, enhanced goblet cell hyperplasia in the allergic epithelium. CircZNF652 is specifically and significantly expressed within airway epithelium in asthmatic children and OVA‐mediated experimental mouse models.^158^ CircZNF652 upregulation promotes metaplasia of bronchial goblet cells and result in excess mucus production. CircZNF652 can sponge miR‐452‐5p and activate JAK2/STAT6 axis to promote goblet cell metaplasia.^158^ ESRP1, which is a splicing factor, can facilitate circZNF652 expression, leading to goblet cell metaplasia.^158^ The above results indicate that ESRP1/circZNF652/miR‐452‐5p/JAK2/STAT6 axis is vital for modulating mucus hypersecretion, goblet cell metaplasia, and AHR within the experimental asthmatic mouse models. Therefore, it is the possible strategy to target circZNF652 and activate miR‐452‐5p to intervene with epithelial remodeling in experimental asthma. However, the precise mechanism related to ESRP1 upregulation in allergic airway epithelium is still unknown. CircZNF652 can regulate RhoA, EIF5, JAK2, and SRSF3. JAK2 is found to be related to goblet cell metaplasia during asthma, while the effects of additional factors on asthma need to be further explored.

##### CircRNAs and immune cells

The airways are infiltrated and activated by different immune cells during asthma, including dendritic cells (DCs), innate lymphoid cells (ILCs), T cells (Th1/Th2/Th9/Th17/Th22 cells), neutrophils, eosinophils, mast cells, and B cells.^159^ The two major types of asthma are type 2 (dominant type) and non‐type 2. Type 2 asthma is generally caused by allergies and nonallergic factors. In contrast, non‐type 2 asthma usually results from infections and contaminants. Once ILCs and Th2 cells, particularly ILC2 cells, detect an allergen, they are capable of secreting cytokines including IL‐4, IL‐5, and IL‐13. Subsequently, these cytokines trigger the recruitment of eosinophils, ultimately causing inflammation in the airways.^159^ Mast cells can produce histamine, thereby facilitating bronchoconstriction, while neutrophils are associated with severe asthma. To initiate immune responses, DCs present antigens to T cells, whereas macrophages accelerate or suppress inflammation according to their M1 or M2 type. Allergies can be triggered by IgE antibodies produced by B cells. It is possible to experience typical asthma symptoms, like bronchoconstriction or acute hypotension, caused by the above‐mentioned immune mechanisms.

Recently, circRNAs have been suggested as important ceRNAs for miRNAs in the function and development of T cells. m_circRasGEF1B, the LPS‐mediated circRNA, is an example suggesting that circRNAs are involved in immunoregulation. It can modulate the ICAM‐1 mRNA stability to promote immunoregulation in human body.^160^ As the cell surface glycoprotein, ICAM1 can be found in certain immune cells and endothelial cells, and it has been identified as the inflammation biomarker. Through the analysis of circRNA expression patterns in diverse cells, different expression patterns are revealed, indicating that they can regulate some cellular processes. For instance, hsa_circ_0012919 downregulation can result in DNA methylation of CD11a and CD70 in CD4 T cells.^161^, ^162^, ^163^, ^164^ Hsa_circ_0045272 may negatively regulate IL2 production and T cell apoptosis through interaction with hsa‐miR‐6127.^158^ CircIKZF1, circTNIK, circTXK, and circFBXW7 are circRNAs with specific expression in T cells.^163^

Recently, circRNAs have been increasingly suggested to be associated with the asthma pathophysiology via regulating immune responses.^165^, ^166^ CD4 T cells are substantially demonstrated to drive disease occurrence by modulating IgE generation upon allergic conditions, generating proinflammatory factors, and recruiting and activating inflammatory cells including neutrophils, eosinophils, and macrophages.^167^, ^168^, ^169^, ^170^, ^171^, ^172^, ^173^, ^174^, ^175^, ^176^ CD4^+^ T cells can produce cytokines. Specifically, Th1 cells produce IFN‐γ, while Th2 cells secrete IL‐4, IL‐5, IL‐6, IL‐9 and IL‐13, and Th17 cells can release IL‐17A and IL‐17F.^177^ In asthma, the complicated immune response can be modulated through the complicated cytokine network.^168^, ^173^, ^174^, ^175^, ^178^ To analyze the effect of circRNAs on CD4 T cells during asthma, Huan et al.^179^ examined the circRNAs expression patterns within CD4 T cells collected in five asthma patients and five normal subjects by micro‐array analysis. Totally 597 circRNAs were abnormally expressed between asthma patients and normal subjects. Among them, hsa_circ_0005519 was most significantly upregulated, as subsequently verified through qRT‐PCR assay in the current cohort samples and in another cohort including 65 asthma individuals and 30 normal controls. Through bioinformatic analysis, literature search and reporter assays, the authors suggested the role of hsa_circ_0005519 in regulating IL13 and IL6 expression, probably by interaction with let‐7a‐5p. IL‐13 and IL‐6 are extensively suggested to be significantly related to the asthma pathogenesis.^167^, ^178^, ^180^, ^181^ Hsa_circ_0005519 expression within CD4 T cells is negatively related to let‐7a‐5p, whereas positively related to IL13 and IL6 mRNA expression, FeNO and peripheral blood eosinophil ratio. As a result, hsa_circ_0005519 is the candidate biomarker for asthma.

In one article, hsa_circ_0002594 was found to be upregulated within CD4^+^ T cells from a present microarray dataset containing five asthma patients and five normal subjects.^179^ For validation, another cohort including 83 asthma patients and 54 normal controls was analyzed, revealing that hsa_circ_0002594 expression was significantly upregulated in CD4^+^ T cells from asthma patients.^179^ Hsa_circ_0002594 expression in CD4+ T cells of asthma patients was positively related to FeNO, but negatively correlated with PD20 (the methacholine dosage necessary for inducing FEV1 decline by 20%). The correlation between hsa_circ_0002594 expression of asthma group and specific clinical factors was analyzed, showing that the hsa_circ_0002594 high‐expression group had increased FeNO expression but decreased PD20 expression in relative to hsa_circ_0002594 low‐expression group. The high‐expression subgroup had more skin prick test (SPT)‐positive and Th2‐high patients than SPT‐negative and non‐Th2 inflammation counterparts. Based on the above results, asthma patients who showed hsa_circ_0002594 high‐expression exhibited clinical indicators consistent with a Th2‐mediated allergic response. Inhaled corticosteroids (ICS) administration significantly reduces hsa_circ_0002594 expression, suggesting that it may be the therapeutic target. Furthermore, hsa_circ_0002594 is sensitive and specific in asthma diagnosis, no matter whether ICS is applied or not. Molecular mechanisms related to hsa_circ_0002594 underlying asthma occurrence were explored by computational research; as a result, possible target miRNAs were identified, proposing the role of hsa_circ_0002594 in the competitive sequestering of the activities of hsa‐miR‐503‐5p, hsa‐let‐7e‐5p, hsa‐miR‐587, hsa‐miR‐16‐5p, and hsa‐miR‐514a‐3p. However, this study still has the following limitations: (1) The sample size was small, and thus large‐scale and more studies are warranted, (2) experimental verification for miRNA‐hsa_circ_0002594 interaction was not available, and (3) the hsa_circ_0002594 level in circulation was not evaluated for its potential as a biomarker.

Mmu_circ_0001359 has been recently found to be downregulated within lungs in the murine model of OVA‐mediated asthma.^182^ Delivering adipose‐derived stem cells (ADSCs)‐derived exosomes after mmu_circ_0001359 modification can alleviate airway remodeling by activating M2‐like macrophages via miR‐183‐5p/FoxO1 pathway.^182^

CircRNAs expression within lungs in mice with HDM‐induced experimental asthma was analyzed.^183^ The circRNA–miRNA axis was explored, which identified two circRNAs with upregulated expression, including circ_0000629 and circ_0000455 targeting miR‐29b and miR‐15a separately. Previous research reported that such miRNAs were inversely related to allergic reactions.^134^, ^184^ By contrast, two circRNAs with downregulated expression, namely, circ_0000723 and circ_0001454, target miR‐214 and miR‐146b, respectively. MiR‐214 and miR‐146b show a positive relationship to asthma.^135^, ^185^ Therefore, the aforementioned four circRNAs are candidates deserving subsequent analysis concerning the association with asthma.

#### 3.1.3 CircRNAs in COPD

CircRNAs have been increasingly suggested to be related to the COPD pathophysiology. TO identify circRNAs related to COPD, some profiling research is conducted using NGS technology. For instance, Duan et al.^186^ explored the circRNA expression patterns in peripheral blood mononuclear cells (PBMCs) from 21 COPD cases together with 21 normal subjects. The results showed that 2132 DEcircRNAs and 2734 DEmRNAs were obtained from COPD cases. As a result of further investigation, circ_0008672 was related to some pivotal COPD pathways, including the NOD‐like receptor pathway, NK cell‐driven cytotoxicity, and Th17 cell differentiation. A circRNA–miRNA–mRNA network was established to investigate how circRNAs can act as miRNA sponges in COPD. According to the results, circRNAs modulated mRNAs through sponging at least one miRNA. For example, circ_0008672 interacts with miR‐1265 to modulate MAPK1 expression. Activation of MAPK pathway is crucial for the COPD pathophysiological characteristics, like airway mucus hypersecretion, lung inflammation, T‐cell activation, and airway fibrosis.^187^ Therefore, the ceRNA network is of great importance for COPD. However, there are some limitations. The results should be validated with a larger COPD cohort, the accurate functions of circRNAs must be experimentally investigated, and the PBMC subsets need to be further assessed.

According to serum samples from 21 smokers and 24 smokers with COPD compared with 17 nonsmokers, Circ‐HACE1 is overexpressed in serum samples from both groups.^188^ Circ‐HACE1 was significantly upregulated in cigarette smoke extract (CSE)‐exposed human bronchial epithelial cells (16HBE cells), serving as the in vitro COPD model.^188^, ^189^ The siRNA‐based targeting of circ‐HACE1 expression improved the CSE‐mediated injuries to 16HBE cells, which was supported by the enhanced cell viability, decreased oxidative stress, apoptosis, and inflammation. The obtained benefits were attained by the interaction of circ‐HACE1 with miR‐485‐3p to regulate TLR4 expression. TLR4 exerts a vital impact on innate immune activation. Besides, it is tightly associated with inflammatory responses in certain diseases, like COPD. The above results show that circ‐HACE1 is the candidate target for enhancing the COPD diagnostic accuracy; moreover, suppressing circ‐HACE1 may be beneficial for the treatment of COPD.

Moreover, circ_0040929 expression is significantly upregulated in the serum samples collected from COPD patients and within CSE‐exposed 16HBE cells.^190^ Silencing circ_0040929 protects CSE‐treated 16HBE cells from injuries by regulating miR‐515‐5p/IGFBP3 pathway. Circ‐RBMS1, also called hsa_circ_0002136, is derived from RBMS1 gene and overexpressed within PBMCs in COPD patients.^186^ Nevertheless, its functions and associated molecular mechanisms are not explored through circRNA profiling and bioinformatic analysis. Similarly, Qiao et al.^191^ discovered that circ‐RBMS1 was upregulated in COPD patients.^191^ Circ‐RBMS1 was dose‐dependently upregulated within CSE‐exposed 16HBE cells. Functional analysis indicated that siRNA‐mediated circ‐RBMS1 suppression attenuated CSE‐induced injuries in 16HBE cells. From the mechanism perspective, circ‐RBMS1 interacted with miR‐197‐3p to regulate FBXO11, which belongs to the F‐box family of proteins and is involved in substrate degradation, ubiquitination, and genome stability modulation. FBXO11 is previously found to be related to inflammation, apoptosis and airway remodeling resulting from CSE.^192^ Nevertheless, the above results were acquired based on in vitro analysis. Therefore, larger COPD cohorts and in vivo assays need to be performed to verify the above findings.

CircFOXO3 displays upregulation within CSE‐exposed mouse alveolar epithelial cells and lungs in mice with CS‐induced experimental COPD.^193^ However, lentivirus‐mediated CircFOXO3 knockdown mitigates CS‐induced mouse lung inflammation through CircFOXO3/miR‐214‐3p/NF‐κB pathway, targeting the CS‐induced inflammatory responses within mouse lungs. Therefore, targeting circFOXO3 is the new way to prevent CS‐mediated inflammation.

Circ‐OSBPL2 modulates apoptosis, inflammation and oxidative stress of CSE‐exposed HBECs by regulating miR‐193a‐5p/BRD4 pathway.^194^ Circ_0061052 expression increases within CSE‐exposed HBECs, which can regulate CS‐induced EMT and airway remodeling by sponging miR‐515‐5p, consequently modulating downstream targets Snail and FoxC1.^195^ Circ_0026466 is upregulated within blood from CSE‐exposed HBECs and smokers with COPD.^196^ Circ_0026466 knockdown alleviates the CSE‐mediated suppression of proliferation and viability, promotes apoptosis, inflammation, and oxidative stress of HBECs via circ_0026466/miR‐153‐3p/TRAF6/NF‐κB pathway.^196^ Similarly, circXPO1 expression increases within lungs in mice with CS‐induced experimental COPD and CSE‐exposed AT2 cells.^197^ Inhibiting circXPO1 can inhibit CSE‐induced cell aging and inflammatory response. Mechanistic analysis indicates that circXPO1 can sponge miR‐23b‐3p to regulate TAB3 mRNA expression, thus modulating cell aging and inflammatory response. Therefore, circXPO1 may sponge miR‐23b‐3p to accelerate COPD pathogenesis, thereby regulating TAB3 expression.

On the contrary, circRNAs knockdown promotes the COPD pathogenesis. For example, circ_0006892 expression decreased in lungs of COPD cases and CSE‐exposed BEAS‐2B and 16HBE cells based on qRT‐PCR results.^198^ Circ_0006892 exhibited a positive relationship to FEV1% of COPD patients. To explore the function of circ_0006892, the gain‐of‐function (GOF) test was performed on CSE‐exposed BEAS‐2B and 16HBE cells to evaluate cell apoptosis, survival, and apoptosis. The findings indicate that circ_0006892 upregulation based on plasmid transfection decreased the CSE‐induced inflammatory response and cell apoptosis, suggesting that circ_0006892 protected against the CSE‐induced bronchial epithelial injury. Comparatively, circ_0006892 silencing promoted inflammation and apoptosis of CSE‐induced HBECs via miR‐24/PHLPP2 axis. Nevertheless, the study only analyzed the in vitro effect of circ_0006892. In this regard, the conclusions might not precisely indicate the complicated environment among COPD patients. To comprehensively comprehend the COPD pathogenesis, more research based on preclinical animal models is required.

CircRNAs can regulate pulmonary smooth muscle and endothelial cells in the in vitro COPD models. For instance, circANKRD11 is upregulated within lungs in smokers with or with no COPD as well as within CSE‐exposed human pulmonary microvascular endothelial cells (HPMECs).^199^ CircANKRD11 silencing within HPMECs lowers the CSE‐induced cell apoptosis, oxidative stress, and inflammatory response by regulating miR‐145‐5p/BRD4 axis. CircBPTF is upregulated within pulmonary arteries in COPD patients, which facilitates hypoxic pulmonary artery smooth muscle cells (PASMCs) proliferation. The circBPTF/miR‐486‐5p/CEMIP pathway enhances PASMC proliferation.^200^ Dysfunction of endothelial cells and PASMCs can be observed in pulmonary arterial hypertension, and these cells make great contributions to lung damage and chronic vascular inflammation in COPD. More investigations need to be performed to explore the effects of circRNAs on the above cells by clinical and in vivo studies. On the whole, circRNAs are dysregulated in COPD cells, which exert important effects on disease occurrence and development. Nonetheless, the above results are mainly obtained based on in vitro assays and cannot comprehensively represent the complicated environment in COPD patients. To further comprehend COPD pathogenesis and explore functions of circRNAs, more investigations are warranted by using preclinical animal models.

#### 3.1.4 CircRNAs as emerging biomarkers for asthma and COPD

CircRNAs are previously indicated to be stable due to the closed‐loop structures, conversed degree among different species, and developmental stage‐ and tissue‐specific expression patterns. Furthermore, circRNAs exhibit high abundance within saliva, blood, urine, and exosomes; as a result, they can be sensitively and specifically detected. Considering the above distinct characteristics, circRNAs may be used to discover biomarkers.

CircRNAs are the candidate biomarkers for COPD and asthma. For example, based on receiver operating characteristic (ROC) curve for circ_0002594 expression within CD4+ T cells collected in 83 asthma patients without ICS exposure, the area under the curve (AUC) was extremely high (0.727).^201^ Similarly, the AUC value was 0.767 for evaluating the diagnostic performance of circ_0002594 in 48 asthma cases receiving ICS treatment.^201^ Circ_0002594 expression is positively related to family history, exhaled nitric oxide level, Th2 cytokine expression, and positive SPT results.^201^ CircSORT1 and circSERPINB1 were upregulated within induced sputum from 68 asthma cases in relative to 20 normal controls, suggesting that they were more specific and sensitive in asthma prediction.^202^ CircSORT1 expression is related to different clinical factors of asthma, like EOS%, FeNO, IFN‐γ, IL‐17A, and PC20.^202^ CircSERPINB1 expression is associated with IL‐6, IFN‐γ, IL‐17A, FVC%, and FEV1%.^202^ Furthermore, circ_0005519 expression markedly elevated within CD4^+^ T cells and PBMCs from 30 asthma patients in comparison with 24 normal subjects, and the upregulation was correlated with the exhaled nitric oxide level.^179^ In addition, circ_0001859 expression in serum decreased among 38 COPD patients and 24 acute exacerbation of COPD (AE‐COPD) patients in relative to 28 normal subjects. The downregulation was highly specific and sensitive in the prediction of COPD and AE‐COPD, which was also related to FEV1% predicted. Furthermore, circ_004092 expression increases within blood collected from smokers with or with no COPD (*n* = 22 each) compared with nonsmokers (*n* = 22). Collectively, circRNAs may be the candidate biomarkers, which can be used to diagnose and predict the prognosis of asthma and COPD. Nevertheless, large sample cohorts are warranted to confirm whether circRNAs can be the biomarkers for asthma and COPD.

#### 3.1.5 CircRNAs in acute respiratory distress syndrome

Acute respiratory distress syndrome (ARDS), the severe stage or type of acute lung injury (ALI), is a major fatal respiratory disease characterized by respiratory failure and refractory arterial hypoxemia. Despite extensive research efforts, progress in understanding the pathogenesis and developing effective therapies for ARDS/ALI is slow, causing the increased incidence and mortality. miRNAs like miR‐155^203^ and miR‐17^204^ are previously suggested to be crucial for ALI occurrence and management. CircRNAs can sponge miRNAs, which provide a novel perspective for developing effective therapies against ARDS. Wan et al.^205^ examined circRNA expression patterns in lung tissues collected from LPS‐mediated ARDS rats, and identified 395 upregulated and 562 downregulated circRNAs. Among them, four upregulated circRNAs (mmu_circRNA_19423, mmu_circRNA_30664, rno_circRNA_010489, rno_circRNA_011426) and one downregulated circRNA (rno_circRNA_005564) showed significant changes. Ye et al.^206^ obtained 10 DEcircRNAs in rat lungs after smoke inhalation‐mediated ALI. According to Zhong et al.,^207^ circFLNA exhibited abnormal upregulation within ARDS, and circFLNA depletion increased CD4^+^CD25^+^Foxp3^+^ Tregs level while reducing the inflammatory response. These changes in circRNAs shed more lights on the biological roles and molecular mechanisms associated with circRNAs in ARDS/ALI. However, further research is warranted to identify their specific roles and mechanisms and to demonstrate these findings in patients with ARDS/ALI.

#### 3.1.6 CircRNAs in pulmonary hypertension

Pulmonary hypertension (PH) is the pathophysiological and hemodynamic state characterized by the elevation of pulmonary arterial pressure to higher than the specific threshold. Many ncRNAs (miRNAs and lncRNAs) are related to the PH pathogenesis. CircRNAs, belonging to the ncRNA family, are emerging as key diagnostic biomarkers and therapeutic targets for PH. Based on our knowledge, our group is the first to identify dysregulation of circRNAs within lungs from hypoxia‐related PH mice through circRNA microarray analysis. Among them, 23 circRNAs showed significant upregulation, while 41 exhibited significant downregulation. Furthermore, we employed circRNA–miRNA–mRNA network analysis, Gene Ontology (GO) as well as Kyoto Encyclopedia of Genes and Genomes (KEGG) analysis for shedding more lights on associated pathways and mechanisms. The findings indicated that dysregulated circRNAs were vital for the hypoxia‐related PH pathogenesis, which were the candidate therapeutic targets.^208^ Miao et al.^209^ obtained 351 DEcircRNAs (including 122 with upregulation whereas 229 with downregulation) in chronic thromboembolic pulmonary hypertension (CTEPH). Based on their findings, upregulated circRNAs probably affected ribonucleotide biosynthesis to exert their effect on CTEPH, while downregulated ones might regulate cellular response to stress, gene expression and DNA damage stimulus to exert their impact. Among them, hsa_circ_0002062 sponged hsa‐miR‐942‐5p and was mostly related to cancer‐related pathways, while hsa_circ_0022342 sponged hsa‐miR‐940 and was primary expressed in ErbB signaling pathway. Both of them are essential for CTEPH occurrence, and targeting them is the efficient way to treat CTEPH. Based on the obtained findings, circRNAs are crucial for PH occurrence, while they concentrate on analyzing the dysregulated circRNAs expression patterns, while there still lacks further functional and mechanistic research.

#### 3.1.7 CircRNAs in pulmonary tuberculosis

Pulmonary tuberculosis (TB) results from Mycobacterium TB, which still seriously threatens public health due to the delayed diagnosis and management. The existing diagnostic approaches of pulmonary TB show unsatisfactory specificity and sensitivity. Therefore, novel biomarkers should be identified to diagnose and treat pulmonary TB early. Zhang et al.^210^ obtained 170 circRNAs with dysregulation in pulmonary TB patients relative to normal controls and established the ceRNA networks. According to the results, circRNA‐related ceRNA‐dependent gene regulation was critical for regulating the pulmonary TB pathogenesis. Based on Huang et al.,^211^ hsa_circ_001937 was important for TB diagnosis (AUC = 0.873), which was related to TB severity and could be the TB‐specific signature circRNA; besides, it was significantly upregulated among TB patents in comparison with pneumonia, lung cancer and COPD patients. According to Zhuang et al.,^212^ hsa_circ_0005836 and hsa_circ_0009128 showed decreased expression within PBMCs of active pulmonary TB patients in relative to normal subjects. GO and KEGG analysis revealed that the main biological functions of DEcircRNAs were associated with immune system activation, indicating that TB infection was related to immune system activity. Qian et al.^213^ obtained DEcircRNAs in PBMCs of TB patients and chose seven circRNAs for the construction of the circRNA‐based TB index for all patients. TB patients displayed the increased TB index relative to normal controls, with the AUC value being 0.946 for the validation groups. Through KEGG enrichment, multiple pathways associated with bacterial invasion and inflammation were enriched into active TB patients. Yi et al.^214^ analyzed circRNA expression patterns in serum samples collected from active TB cases. According to their results, hsa_circRNA_103571 expression markedly decreased among active TB patients. Based on bioinformatics analysis, hsa_circRNA_103571 was related to regulating T‐ and B‐cell receptor and actin cytoskeleton pathways. Certain circRNAs are previously demonstrated with diagnostic significance for TB infection. Future studies need to concentrate on nontuberculous mycobacteria (NTM) that cause TB‐like imaging findings and clinical symptoms, which may usually result in misdiagnosis and mistreatment. Most NTM are highly resistant to anti‐TB agents, which imposes significant disease burden on the society. CircRNAs probably serve as novel biomarkers to help physicians differentiate NTM infections from TB.

#### 3.1.8 CircRNAs in silicosis

Silicosis is a frequently seen, rapid‐growing and severe pneumoconiosis type, exhibiting the representative characteristic of excessive pulmonary nodular fibrosis resulting from long‐time inhalation of excess free silica dust. Currently, circRNAs related to silicosis are studied extensively, primarily by the Jie Chao's group. CircZC3H4 RNA is found to sponge miR‐212, relieving the miR‐212‐induced ZC3H4 protein suppression. CircZC3H4 RNA and ZC3H4 upregulation after exposure to SiO2 affects macrophage activation as well as the subsequent influences on fibroblast growth and invasion. ZC3H4 protein upregulation is revealed in tissues of silicosis patients, exhibiting the feasibility of ZC3H4 as the candidate therapeutic target for silicosis.^215^

According to Zhou et al.,^216^ ircHEZCD1/HECTD1 pathway was associated with SiO2‐mediated macrophage activation and HECTD1 upregulation among silicosis patients. In the meanwhile, circHEZCD1 downregulation and HECTD1 upregulation after SiO2 exposure are modulated via ZC3H12A, the new RBP. Following this, Fang et al.^217^ discovered the possible role of circHECTD1 in regulating HECTD1 (the host gene) protein expression, through competing with the corresponding pre‐mRNA. The circHECTD1/HECTD1 pathway, which promotes EMT in vitro after SiO2 exposure, is the candidate mechanism underlying fibrosis and the novel therapeutic target for pulmonary silicosis patients.

### 3.2 CircRNAs in endocrine and metabolic diseases

#### 3.2.1 CircRNA in diabetes

##### Type 1 diabetes

Type 1 diabetes (T1D), also called insulin‐dependent diabetes, is the chronic autoimmune disorder with the representative characteristic of β‐cell destruction and dysfunction, absolute insulin deficiency, and increased blood glucose content.^218^ Substantial evidence demonstrates the effects of circRNAs on autoimmune disorders, partially by evaluating autoimmune disorders in T1D research.

There were 61 circRNAs with upregulation while 7 with downregulation detected within plasma from new‐onset T1D patients, with four circRNAs (hsa_circRNA_100332, hsa_circRNA_101062, hsa_circRNA_085129, and hsa_circRNA_103845) being demonstrated in succession using quantitative real‐time PCR.^219^ There were 93 DEcircular transcripts detected from peripheral blood in T1D patients relative to normal subjects, with 30 being upregulated while 63 being downregulated. Based on GO and KEGG analyses, such circRNAs contributed to T1D occurrence through diverse pathways. Hsa_circ_0072697 was the possible endogenous miRNA sponge to regulate β‐cell activity.^220^ The above dysregulated circRNAs serve as potential biomarkers for T1D, which should be further validated based on longitudinal studies. CircRNAs are related to the inflammatory response during T1D, which may offer the new therapeutic target for the disease. CircPPM1F is increasingly suggested to be mainly expressed within and promotes M1 macrophage activation through circPPM1F–HuR–PPM1F–NF‐κB pathway. CircPPM1F upregulation may aggravate injuries to pancreatic islet.^221^ Macrophages, which are the immune response inflammatory mediators, are essential for insulitis. Besides, they can support autoimmune T cells during T1D through promoting the infiltration of inflammatory cells.^222^ As discovered by Yang et al.,^223^ hsa_circ_0060450 expression increased in T1D patients, and it sponged miR‐199a‐5p for suppressing macrophage‐induced inflammation through JAK‐STAT pathway. Moreover, 1922 significantly expressed circRNAs were detected in mouse islet β‐cells after induction of different cytokines, including interleukin‐1β (IL‐1β), TNF‐α, and INF‐γ.^224^ To analyze the impacts of circRNAs on T1D, Yi et al. constructed a circRNA–lncRNA–miRNA–mRNA ceRNA network of T1D by computational and bioinformatic analyses.^225^ Nonetheless, there is limited research on therapeutic circRNAs for T1D, and a larger sample size is needed for further validation.

##### Type 2 diabetes

Type 2 diabetes (T2D) has an increasing incidence rate due to the growing living standards and the changed diet structures, bringing significant health burdens on the patients and society.^226^, ^227^ The function of circRNAs in T2D pathogenic mechanisms and development has recently been explored, suggesting that circRNAs may serve as diagnostic biomarkers and therapeutic targets in diabetics.

Serum hsa_circ_0054633 was used to diagnose prediabetes and T2D among 63 prediabetics, 64 T2D patients and 60 normal controls.^228^ Serum hsa_circ_0054633 expression among T2D patients was related to low‐density lipoprotein, hemoglobin A1c, and fasting blood glucose levels.^229^ Hsa_circ_0063425 combined with hsa_circ_0056891 could differentiate T2D patients and those with fasting glucose impairment from normal subjects among 313 subjects, and the AUC values were 0.837 and 0.719, respectively.^230^ Hsa_circ_0063425 and hsa_circ_0056891 are found to sponge miR‐19a‐3p and miR‐1‐3p, respectively, aiming to exert crucial effects on insulin phosphatidylinositol 3‐kinase/protein kinase B pathway.^230^ Furthermore, circHIPK3 and CDR1as have increased expression among T2D patients.^231^ CircHIPK3, also known as hsa_circ_0000284, is a highly abundant circRNA within pancreatic islets and is obtained based on exon 2 in HIPK3 that is conserved between mouse and human cells.^232^ CircHIPK3 facilitates insulin resistance and hyperglycemia by increasing FOXO1 expression and sponging miR‐192‐5p.^233^ CircHIPK3 expression declines within DB/DB islets. CircHIPK3 knockdown promotes β‐cell apoptosis while decreasing glucose‐stimulated insulin production. CircHIPK3 significantly affects islet cells partially by sponging miR‐29‐3p, miR‐30, miR‐124‐3p, and miR‐338‐3p, which is achieved through regulating critical β‐cell genes like Slc2a2, Mtpn, and Akt1.^234^ The above miRNAs are widely suggested to significantly affect β‐cell proliferation, development, function, and survival.^235^, ^236^, ^237^, ^238^ CircHIPK3 can differentiate T2D patients from normal subjects, and the specificity and sensitivity are 88.4 and 50.0%, respectively.^231^ CDR1, first discovered in brain tissue^239^ is widely expressed in pancreatic β‐cells. CDR1as is reported to modulate insulin production and transcription in islet cells by inhibiting miR‐7 through regulating endogenous target genes Pax6 and Myrip.^240^ CDR1as expression is downregulated within OB/OB and DB/DB mice, conforming to its effect on diabetes occurrence. Furthermore, CDR1as overexpression increases islet cell insulin production and insulin level,^240^ while CDR1as knockdown has opposite effects and reduces the prolactin‐mediated MIN6B cell proliferation in isolated rat islets.^234^ CDR1as is shown to differentiate prediabetics from normal controls (AUC = 0.605).^231^

Circ‐Tulp4 is the candidate treatment against T2D.^241^ Based on in vitro experimental results, circTulp4 modulated MIN6 cell proliferation. Its overexpression upregulated SOAT1, which then increased cyclin D1 expression, finally promoting cell cycle progression while alleviating β‐cell dysfunction by inhibiting miR‐7222‐3p.^241^ CircGlis3, the exocircRNA obtained in β‐cells, exhibits higher expression within serum samples collected from T2D patients and diabetic mice. It is translocated in islet endothelial cells and serves as the therapeutic target.^242^ CircGlis3 is related to lipotoxicity‐mediated β‐cell disease and diabetes through inhibiting cell growth and insulin production. From the mechanism perspective, circGlis3 can decrease islet endothelial cell invasion and viability and is transported to target cells by exosomes. Intronic circRNAs produced by insulin genes exert a vital impact on T2D occurrence. For instance, circular intronic‐Ins2 (ci‐Ins2) is found to modulate β‐cell activity.^243^ CircRNA containing the lariat sequence of intron 2 in insulin gene is reported to modulate insulin production in T2D patients and diabetes rodent models, which may clarify the impairment of secretion ability.^244^ CircANKRD36 is also associated with chronic inflammatory factors in T2D. According to subsequent functional analysis, circANKRD36 expression was positively correlated with IL‐b and blood glucose levels.^245^ Furthermore, dysregulated circRNAs is correlated with pancreatic β‐cell autophagy in T2D rats, which may result from rno_circRNA_008565 after interacting with miRNAs.^246^ Collectively, circRNAs are potentially related to inflammatory response, insulin resistance, and β‐cell failure within T2D, and may be the candidate therapeutic target for T2D.

##### Gestational diabetes mellitus

CircRNAs are crucial for the prediction of several adverse reactions among gestational diabetes mellitus (GDM) patients. CircVEGFC, the new regulatory factor for glucose metabolism, has elevated expression among GDM pregnant women. Its upregulation is related to hypertension and fetal malformations.^247^ Pregnant women with circACTR2 upregulation may experience GDM. According to Zhu et al.^248^ aberrant circACTR2 expression was related to premature delivery, fetal malformations, miscarriage and intrauterine infection. Therefore, circRNAs are the candidate biomarkers that can be used for predicting the complication risk of GDM patients. However, their clinical application and the associated mechanisms should be further explored.

GDM is the frequently seen pregnancy complication, which has seriously threatened the maternal and fetal health. CircRNAs are found to be of therapeutic significance. For example, placental villous circRNAs are explored for their expression patterns in GDM women, suggesting that the dysregulated circRNAs are probably associated with GDM.^249^, ^250^ Chen et al.^251^ discovered the high circ_0008285 expression among GDM patients, and circ_0008285 depletion inhibited cell growth, migration, and invasion. Hsa_circ_0005243 is related to suppressing cell growth and invasion and promoting IL‐6 and TNF‐α expression within GDM via β‐catenin/NF‐κB pathway.^252^ CircPNPT1 expression increases within placental tissues in GDM patients and high glucose (HG)‐stimulated trophoblast cells.^253^ At the mechanism level, circPNPT1 sponges miR‐889‐3p and activates p21‐activated kinase 1 to modulate trophoblast cell dysfunction. CircPNPT1 is packaged in exosomes and subsequently delivered to additional cells.^253^

##### Effects of circRNAs on diabetic complications

**FIGURE 4:**
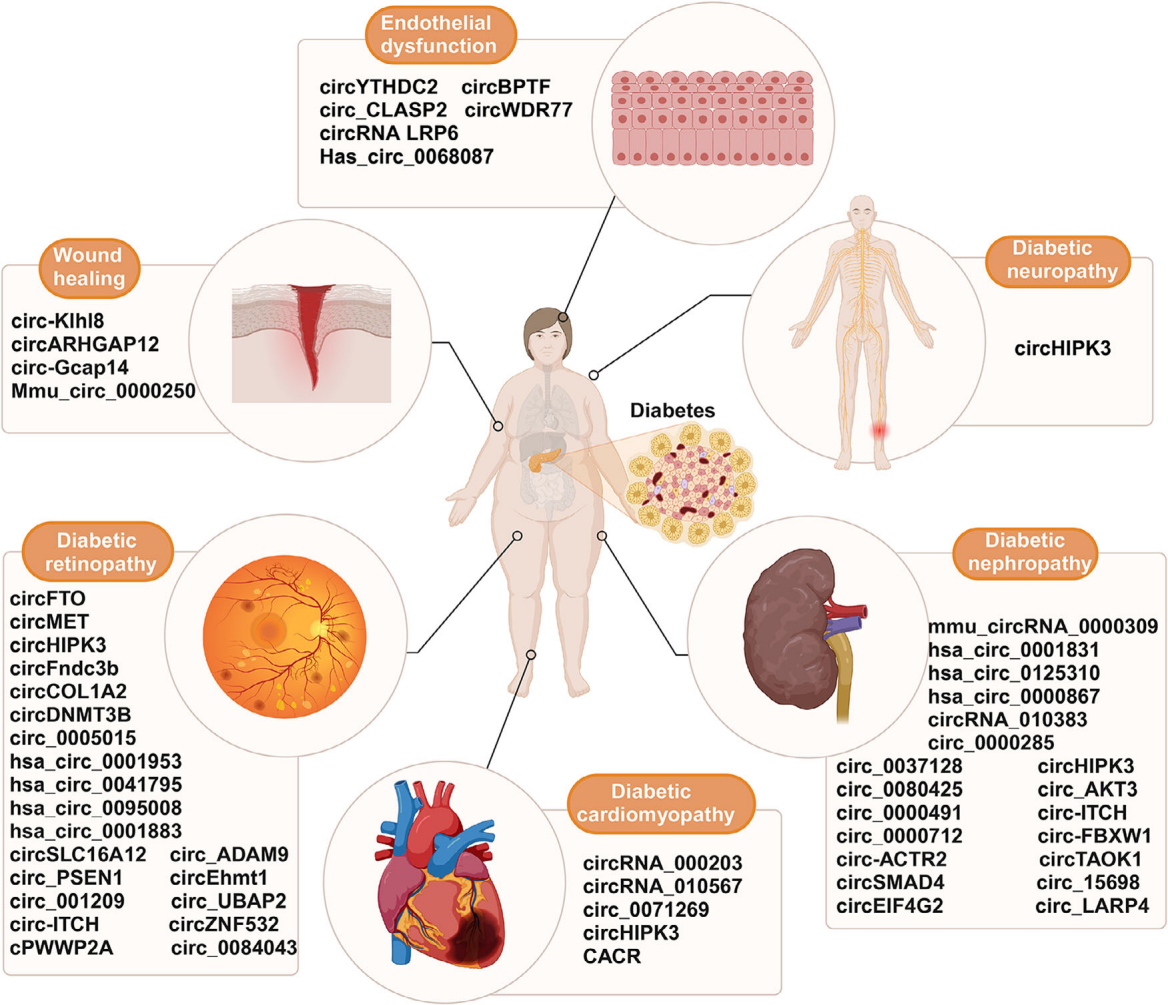
The role of circRNAs in diabetic complications. This figure summarizes the involvement of circRNAs in various diabetic complications, including diabetic retinopathy (DR), diabetic nephropathy (DN), diabetic neuropathy (DNP), diabetic cardiomyopathy (DCM), as well as high glucose‐related endothelial dysfunction and impaired wound healing.

Diabetes complications can result from persistent hyperglycemia, including diabetic retinopathy (DR), diabetic nephropathy (DN), diabetic neuropathy (DNP), diabetic cardiomyopathy (DCM), and HG‐mediated endothelial dysfunction. The complications have seriously threatened human life. CircRNAs are widely suggested to play a key role in the occurrence of diabetic complications. Figure 4 displays circRNAs that are verified to be related to diabetes‐related disorders.

##### CircRNAs in DR

DR can be classified as proliferative or nonproliferative DR based on its severity.^254^ DR, the frequent diabetes‐related microvascular complication, is the major factor inducing blindness among working‐aged individuals.^255^ It is correlated with alterations of endothelial cells and pericytes and shows the typical features of vascular leakage and capillary occlusion.^255^ CircRNA dysregulation is linked with DR progression; therefore, they may be the key biomarkers and reasonable therapeutic targets.

With regard to proliferative DR, circRNAs are the diagnostic biomarkers. According to Zhang et al.,^256^ the blood hsa_circ_0001953 expression increased in proliferative DR individuals in relative to nonproliferative DR counterparts and normal subjects. Nevertheless, this should be verified with a larger population. Totally 529 dysregulated circRNAs were detected from two diabetic human retinas. Among them, hsa_circ_0005015 had increased expression within plasma, fibrovascular membrane, and vitreous samples in DR individuals. It promoted retinal endothelial angiogenesis through modulating endothelial cell growth, tube formation, and migration. Hsa_circ_0005015 was the candidate diagnostic biomarker for DR. Subsequent functional experimental analysis showed that hsa_circ_0005015 sponged miR‐519d‐3p to inhibit miR‐519d‐3p activity, thus upregulating XIAP, MMP‐2, and STAT3.^257^ Hsa_circ_0001883 and hsa_circ_0095008 have been recently identified as early diagnostic biomarkers for DR patients, and the AUCs for diagnosis are 0.607 and 0.671, respectively.^258^

Circhipk3 is upregulated in retinal endothelial cells and diabetic retinas, which blocks mir‐30a‐3p to increase the expression of vascular endothelial growth factor‐C (VEGF‐C), WNT2 and FZD4, resulting in vascular dysfunction and endothelial proliferation.^259^ circfto, circdnmt3b, circcol1a2, circ_001209, circslc16a12, circ‐UBAP2, and exosomal circfndc3 can modulate the diabetic retinal vascular function.^260^, ^261^, ^262^, ^263^, ^264^ They are the candidate targets for controlling DR. CircDNMT3B knockdown aggravated visual injury, while its upregulation alleviated retinal vascular dysfunction. By contrast, upregulation of circ_001209 sponges miR‐15b‐5p to exacerbate retinal injury of diabetic rats.^265^, ^266^ CircRNAs affect the pericyte‐endothelial cell crosstalk to modulate DR.^267^, ^268^ cPWWP2A, the new DR‐related circRNA, is the ceRNA interacting with miR‐579 for promoting retinal vascular dysfunction by upregulating angiopoietin 1, SIRT1, and occludin.^267^ Interestingly, cPWWP2A is expressed on pericytes rather than on endothelial cells, while it has indirect regulation on endothelial cell biology via exosomes. Pericytes‐derived exosomal circEhmt1 is transported into endothelial cells, where it prevents from HG‐induced injury.^268^ CircRNAs are essential for human retinal pigment epithelial (ARPE‐19) cells. CircRNA_0084043 and hsa_circ_0041795 are significantly upregulated within HG‐mediated ARPE‐19 cells, which can decrease their growth and survival. At the mechanism level, hsa_circ_0041795 sponges miR‐646 to facilitate HG‐induced apoptosis in ARPE‐19 cells by activating VEGFC miR‐646.^269^ CircRNA_0084043 deficiency sponges miR‐140‐3p and induces TGFA to significantly increase HG‐exposed ARPE‐19 cell apoptosis. CircRNA_0084043 deficiency efficiently suppresses HG‐induced inflammation by suppressing the inflammatory factors TNF‐α, Cox‐2, and IL‐6 within ARPE‐19 cells.^270^ Circ‐PSEN1 and circ‐ADAM9 are upregulated in DR. Circ‐ADAM9 promotes injuries to ARPE‐19 cells, while circ‐PSEN1 knockdown alleviates ferroptosis.^271^, ^272^ CircZNF532 modulates pericyte biology through sponging miR‐29a‐3p, thereby upregulating NG2, CDK2, and LOXL2 expression. Besides, it regulates pyroptosis and apoptosis of ARPE‐19 cells to facilitate inflammation and angiogenesis in DR.^273^, ^274^, ^275^ Circ‐ITCH is related to the DR pathology and physiology in rat retinal pigment epithelial cells.^276^ Recent research has shown that silencing circMET can reduce retinal angiogenesis.^277^ CircRNAs are involved in DR progression and may be used for the treatment of the disease.

##### CircRNAs in DN

DN is a major factor inducing mortality among diabetics.^278^ Its pathological features include progressive ECM deposition, basement membrane thickening, persistent mesangial cells (MCs) proliferation, and renal fibrosis.^279^ MC and ECM dysfunction play a vital role and serve as early warning signals for DN pathogenesis. It is of great importance to unravel molecular mechanism underlying DN and identify candidate therapeutic targets, aiming to improve patient survival.

Currently, circRNAs are identified as biomarkers for DN. Hsa_circ_0000867 and hsa_circ_0001831 show increased expression within peripheral blood in early type 2 DN cases.^280^ Hsa_circ_0001831 can be used to diagnose early type 2 DN, and the sensitivity, specificity, and AUC values are 0.85, 0.85, and 0.95, respectively.^280^ Hsa_circRNA_102682 is identified to independently predict the risk of DR in one article involving 73 patients), with the AUC for prediction being 0.97.^281^

CircRNAs are widely used to treat DN, including circEIF4G2, circTAOK1, circACTR2, and hsa_circ_0125310.^282^, ^283^, ^284^, ^285^ CircFBXW12 has elevated expression within the serum of DN cases and HG‐induced human MCs. Based on in vitro functional experimental results, circFBXW12 knockdown inhibited human MC proliferation and decreased ECM generation and oxidative stress via miR‐31‐5p/LIN28B pathway.^286^ CircSMAD4 can mitigate ECM accumulation, while circRNA_15698 can promote ECM accumulation.^287^, ^288^ Liu et al.^289^ suggested that circ_0080425 expression was related to DN development, which also positively affected MCs fibrosis and growth via the miR‐24‐3p/FGF11 axis. Circ_0000712 enhances the HG‐induced apoptosis, fibrosis, oxidative stress and inflammation in DN through regulating SOX6 and miR‐879‐5p expression.^290^ Similarly, circ_0000491 sponges miR‐455‐3p to regulate Hmgb1 expression, therefore promoting HG‐mediated apoptosis, fibrosis, oxidative stress and inflammation in DN.^291^ In vivo, circRNA_010383 upregulation suppresses miR‐135a expression, increases transient receptor potential cation channel, subfamily C, and member 1 expression, and suppresses kidney fibrosis and proteinuria of DN mice.^292^ Circ_0000285 and circ_0037128 are upregulated within the DN mouse models. Circ_0000285 sponges miR‐654‐3p to activate MAPK6 expression and induce podocyte injury.^293^ Circ_0037128 has been recently suggested to modulate DN occurrence by miR‐17‐3p/AKT3 pathway.^294^ Circ_0037128 is reported to suppress HK‐2 cell growth and enhance their fibrosis and inflammation by miR‐497‐5p/NFAT5 pathway. By contrast, circAKT3, circLARP4, circITCH, and mmu_circRNA_0000309 expression decreases within DN rats and mice, as well as in DN cell models and tissues.^224^, ^295^, ^296^, ^297^ Upregulation of circAKT3 sponges miR‐296‐3p to suppress the fibrosis‐associated protein production, therefore modulating E‐cadherin, and it may be the significant therapeutic target for patients with DN.^295^ CircHIPK3 can modulate insulin production and islet proliferation in order to facilitate insulin resistance. In two articles regarding DN, circHIPK3 significantly influenced rat MCs and human renal tubular epithelial HK‐2 cells. From the mechanism perspective, circHIPK3 expression significantly decreased within HG‐treated HK‐2 cells. CircHIPK3 overexpression reduced the accumulation of miR‐326 and miR‐487a‐3p, thereby upregulating SIRT1 while reducing HG‐mediated suppression on HK‐2 proliferation.^298^ Furthermore, circHIPK3 expression was found to be upregulated within rat MCs. CircHIPK3 knockdown suppressed cell growth and inhibited the mRNA abundance of PCNA, cyclin D1, TGF‐β1, FN, and Col. I within MCs.^299^ However, more investigations are warranted to demonstrate the effect of circHIPK3 on DN.

##### CircRNAs in DNP

DNP, a common diabetic complication, can substantially decline the life quality of diabetic patients. DNP involves the sensory functional loss, which initiates at the distal lower extremities and is featured by stimulus‐evoked and spontaneous pain, along with many illnesses.^300^ Nonetheless, the DNP etiology is still unknown. CircRNAs are found to regulate DNP progression.

NcRNAs, including miRNAs, circRNAs, and long ncRNAs, were analyzed for their expression in streptozotocin‐caused DNP mice. There were totally 148 miRNAs, 135 circRNAs, 30 mRNAs, and nine long ncRNAs with significant dysregulation among DNP mice. They were enriched in Rap1, MAPK, protein digestion, and absorption pathways, and human T‐cell lymphotropic virus‐I infection pathways.^301^ In addition, 133 mRNAs and 15 circRNAs with dysregulated expression were identified within dorsal root ganglia in diabetic and wild‐type mice through chip scanning and high‐throughput RNA sequencing. Meanwhile, there were 14 mRNAs and seven circRNAs closely associated with diabetic peripheral neuropathy.^302^ They are the candidate diagnostic and prognostic biomarkers for DNP.

CircHIPK3 is abundantly expressed in DNP rats and patients. CircHIPK3 overexpression is positively related to neuropathic pain among T2D patients. CircHIPK3 knockdown mitigates neuropathic pain of diabetic rats, which may be probably associated with the reduced neuroinflammation. Mechanistically, circHIPK3 interacts with miR‐124 to downregulate miR‐124. The study provides the first evidence supporting the use of intrathecal circHIPK3 shRNA in the treatment of rat DNP.^303^

##### circRNAs in DCM

DCM is a main factor inducing morbidity and mortality among diabetics and brings significant burdens on the economy and society globally.^304^, ^305^ DCM exhibits the typical features of cardiomyocyte apoptosis, hypertrophy, myocardial interstitial fibrosis, as well as subsequent HF without dyslipidemia, hypertension and coronary artery disease (CAD).^304^, ^306^

CircRNAs are substantially related to DCM development. According to Zhou et al., circRNA_010567 was significantly upregulated within Ang‐II‐induced cardiac fibroblasts (CFs) and the myocardium of diabetic mice. It modulated fibrosis‐associated protein expression within CFs, including Col I, α‐SMA, and Col III by miR‐141/TGF‐β1 pathway.^307^ CircRNA_000203 expression significantly increased within CFs and DCM mouse myocardium. Functional experimental analysis showed that circRNA_000203 upregulation inhibited miR‐26b‐5p and activates CTGF and Col1a2, finally upregulating α‐SMA and Col3a1 within CFs.^308^ Subsequently, CircHIPK3 was found to promote myocardial fibrosis while reducing cardiac function of DCM mice. CircHIPK3 deficiency suppressed CFs proliferation by the Col3a1 and miR‐29b‐3p/Col1a1 regulatory network.^309^ Therefore, circRNA_010567, circHIPK3, and circRNA_000203 are related to the myocardial fibrosis pathogenesis, providing the therapeutic target and molecular mechanism for DCM.

CircHIPK3 is also discovered with downregulated expression in HG‐exposed human cardiomyocytes and in DCM. CircHIPK3 upregulation inhibits the expression of PTEN, the tumor suppressor, and suppresses the HG‐induced apoptosis in human cardiac myocytes.^310^ Circ_0071269 is markedly upregulated in HG‐exposed rat cardiomyocytes. Circ_0071269 silencing can sponge miR‐145 to enhance cell viability and inhibit inflammatory response, pyroptosis, and cytotoxicity of rat cardiomyocytes in vitro, thereby upregulating GSDMA.^311^ Hsa_circ_0076631, called caspase‐1‐associated circRNA (CACR) as well, has elevated expression in HG‐exposed cardiomyocytes and patients with diabetes. CACR knockdown activates caspase‐1 by targeting miR‐214‐3p within cardiomyocytes. On the contrary, miR‐214‐3p deficiency partly reverses the benefits of CACR knockdown for the pyroptosis of cardiomyocytes. Consequently, CACR is the candidate new therapeutic target for DCM through regulating CACR/miR‐214‐3p/caspase‐1 pathway.^312^

##### CircRNAs in hyperglycemia‐associated wound healing and endothelial dysfunction

Endothelial cells constitute the inner vascular lining, which are the anticoagulant barrier between blood and vessel wall.^313^ These unique multifunctional cells regulate vasomotor tone, metabolic homeostasis, vascular smooth muscle cell (VSMC) growth, and immune/inflammatory responses.^314^ Hyperglycemia has been previously suggested to induce endothelial cell dysfunction among diabetics.^315^, ^316^ Hyperglycemia is widely suggested to change circRNA expression, while circRNAs are related to different hyperglycemia‐mediated endothelial dysfunction processes during diabetes.^317^

Human umbilical vein endothelial cells (HUVECs) can be easily obtained and extensively applied for in vitro research on endothelial cells. Based on Cheng et al.,^318^ hsa_circ_0068087 promoted the inflammation and dysfunction of endothelial cells via miR‐197/TLR4/NF‐κB/NLR family pyrin domain containing 3 (NLRP3) pathway. CircBPTF is under close regulation in hyperglycemia‐mediated HUVECs. CircBPTF deficiency targets miR‐384 and downregulates LIN28B to suppress oxidative stress and inflammatory injuries.^319^ Comparatively, circ_CLASP2 expression declines in hyperglycemia‐mediated HUVECs. Circ_CLASP2 regulates the miR‐140‐5p/FBXW7 pathway to prevent hyperglycemia‐induced dysfunction of HUVECs.^320^

Based on significant evidence, circRNAs like circWDR77, circLRP6, and circYTHDC2 show upregulation within hyperglycemia‐exposed VSMCs.^321^, ^322^, ^323^ The above circRNAs enhance VSMCs growth and invasion via diverse mechanisms. Suppressing circWDR77 inhibits VSMC growth and invasion via miR‐124/fibroblast growth factor 2 pathway.^321^ CircYTHDC2 is found to modulate VSMC dysfunction through YTHDC2‐induced m6A modification and regulate ten‐eleven translocation 2 expression.^323^ CircLRP6, which sponges miR‐545‐3p, contributes to the growth and invasion of hyperglycemia‐exposed VSMCs by downregulating HMGA1.^322^

Various circRNAs are related to hyperglycemia‐mediated wound healing. Circ‐Klhl8 expression is enhanced in endothelial progenitor cells under hypoxic pretreatment conditions, whereas circ‐Gcap14 expression is enhanced in ADSCs.^324^, ^325^ Upregulation of circ‐Gcap14 enhances angiopoiesis and accelerates hypoxic ADSC wound closure in diabetics.^324^ Based on Shang et al.,^325^ circ‐Klhl8 promoted autophagy, suppressed hyperglycemia‐mediated endothelial cell injury, and enhanced diabetic wound closure via the miR‐212‐3p/SIRT5 pathway. Recently, exosomal circARHGAP12 and mmu_circ_0000250 are indicated to promote diabetic wound closure by regulating autophagy.^326^, ^327^ From the mechanism perspective, circARHGAP12 can sponge miR‐301b‐3p to enhance diabetic wound healing, thereby regulating target genes ATG16L1 and ULK2 expression.^327^ CircARHGAP12 is the candidate novel therapeutic target for diabetic wound closure.

#### 3.2.2 CircRNAs in adipocyte metabolism

##### Aberrant adipocyte metabolism

Adipocyte metabolism refers to a process to digest, absorb, synthesize and decompose body adipose tissues under the action of different enzymes. Aberrant adipocyte metabolism usually results in further disorders, including metabolic syndrome, hyperlipidemia, AS, and nonalcoholic fatty liver disease (NAFLD).^328^, ^329^, ^330^ Adipocyte metabolism is a complicated physiological process that involves a variety of regulatory factors and genes. It becomes crucial to maintain the adipocyte metabolic homeostasis to prevent and treat adipogenesis‐associated diseases. CircRNAs display specific expression profiles in diverse tissues, cells, and developmental stages, impacting the metabolic processes in vivo.^331^ CircRNAs are increasingly suggested to be associated with adipocyte metabolism and the associated disorders (Table 2).

**TABLE 2:** Functional circRNAs involved in adipocyte metabolism.

CircRNA	Mechanism/partner	Role in fat metabolism	Species	References
hsa_circH19	PTBP1	Promotes hADCSs adipogenic differentiation	Human	^332^
circSAMD4A	miR‐138‐5p/EZH2	Promotes preadipocyte differentiation	Human	^333^
circARF3	miR‐103/TRAF3	Alleviates mitophagy‐mediated inflammation	Mouse	^334^
circ_0075932	PUM2	Promoting effect on inflammation and apoptosis in dermal keratinocytes	Human	^335^
circRNA‐vgll3	miR‐326‐5p	Promotes osteogenic differentiation of adipose‐derived mesenchymal stem cells	Human	^336^
circRNA CDR1as	miR‐7‐5p/WNT5B	Promotes adipogenic and suppresses osteogenic differentiation of BMSCs	Human	^337^
circRNA_013422/circRNA_22566	miR‐338‐3p	Regulates osteogenic differentiation of bone marrow stromal stem cells	Mouse	^338^, ^339^
circRNA‐23525	miR‐30a‐3p	Regulates osteogenic differentiation of adipose‐derived mesenchymal stem cells	Mouse	^340^
circRNA_0000660	miR‐693/Igfbp1	Reduces liver lipid accumulation	Mouse	^341^
circHIPK3	miR‐192‐5p	Enhances fat deposition and triglyceride content in HepG2 cells	Human	^233^
circRNA_0046366	miR‐34a/PPARα	Hinders lipid metabolism and cause liver steatosis	Human	^342^
circRNA_0046367	miR‐34a/PPAR*α*	Alleviates hepatic steatosis	Human	^343^
circRNA_021412	miR‐1972/LPIN1	Contributes to the hepatic steatosis via disrupting the balance of lipogenesis and catalytic separation	Human	^344^
circSCD1	JAK2/STAT5	Promotes the occurrence of fatty liver disease	Mouse	^345^

Abbreviations: BMSC, bone marrow mesenchymal stem cell; EZH2, enhancer of zeste homolog 2; hADC, human adipose‐derived stem cell; HepG2, hepatocellular carcinoma cell line 2; Igfbp1, insulin‐like growth factor binding protein 1; JAK2, Janus kinase 2; PPARα, peroxisome proliferator‐activated receptor alpha; PTBP1, polypyrimidine tract‐binding protein 1; PUM2, pumilio RNA binding family member 2; STAT5, signal transducer and activator of transcription 5; TRAF3, TNF receptor‐associated factor 3; WNT5B, wingless‐type MMTV integration site family, member 5B.

##### CircRNAs modulates adipocyte metabolism within adipose tissue

CircRNAs can impact adipose function in numerous species, including mice, humans, cattle and pigs. As reported by Zhu et al.,^332^ hsa_circH19 exhibited upregulation within the blood in patients experiencing metabolic syndrome. Hsa_circH19 silencing enhanced adipose stem cell differentiation via PTBP1. By conducting sequencing on human subcutaneous and visceral adipose tissues, Arcinas et al.^333^ identified circTshz2‐1 and circArhgap5‐2 as the essential regulatory factors for adipogenesis in vitro. Based on the obtained results, circRNAs expression exerts a vital role in keeping adipocyte metabolism. CircSAMD4A also sponges miR‐138‐5p and modulates EZH2 expression in human obese disorders to control the adipogenesis of obese people.^334^ CircARF3 can sponge miR‐103 to inhibit miR‐103 activity and upregulate TRAF3, miR‐103's downstream target. Then, it alleviates the high‐fat‐diet‐induced inflammation in mouse adipose tissue.^346^

CircRNAs can sponge miRNA within adipose tissue and directly bind to RBPs, thus participating in expression. Circ_0075932 expression exerts a vital role in human normal adipose tissue. Exosomes derived from circ_0075932 overexpression adipose cells significantly enhance cell apoptosis and inflammation. circ_0075932 accelerates cell apoptosis and inflammation through direct binding to RBP PUM2 and enhancing PUM2‐induced AuroraA/NF‐κB pathway activation.^335^

Mesenchymal stem cells (MSCs) are stem cells with high plasticity, which include bone marrow stem cells (BMSCs) and adipose‐derived mesenchymal stem cells (ADSCs). They are capable of self‐renewal and differentiation into bone cells, adipose cells and chondrocytes.^347^ As recently discovered, many circRNAs are related to MSCs adipogenesis.^336^, ^337^, ^348^ For instance, circFOXP1 sponges miR‐17‐3p/miR‐127‐5p and shows indirect regulation of EGFR and noncanonical Wnt pathways, thus maintaining the MSC stemness.^349^ CircRNA CDR1as silencing can promote osteogenic differentiation of BMSCs and suppress their adipogenic differentiation, whereas CDR1as overexpression has opposite effects. Mechanistically, CDR1as competitively binds miR‐7‐5p to WNT5B for enhancing adipogenic differentiation.^337^ CircRNA–vgll3, through the direct sequestering of cytoplasmic miR‐326‐5p and inhibition of miR‐326‐5p activity, promotes osteogenic differentiation of ADSCs.^336^ miR‐338‐3p can upregulate circRNA_013422 and circRNA_22566 in osteogenic differentiation of mouse ADSCs.^338^ miR‐338‐3p targets Fgfr2 and Runx2 to modulate osteogenic differentiation of mouse bone marrow stromal stem cells.^339^ CircRNA_23525 targets miR‐30a‐3p to modulate Runx2 expression, which has been detected as the active regulatory factor for the osteogenic differentiation of ADSCs.^340^

##### CircRNAs and liver lipid metabolism

The liver exerts an essential effect on various metabolic processes, including lipid digestion, absorption, production, transportation, and decomposition. Drink abuse, obesity, and diabetes can induce the liver lipid metabolic disturbance. Excess liver lipid accumulation produces massive reactive oxygen species, which causes mitochondrial dysfunction and endoplasmic reticulum stress within hepatocytes, ultimately inducing NAFLD.^350^, ^351^, ^352^ Numerous circRNAs are suggested to regulate lipid metabolism and influence lipid disorder occurrence.^345^, ^353^

A high‐fat diet in the mother can change liver miRNAs expression in her offspring and impair the metabolic health.^354^ Current research has clarified a novel mechanism underlying the role of maternal obesity in liver lipid metabolism of offspring. Through liver RNA sequencing on offspring of high‐fat‐diet‐fed C57BL/6J mice, Chen et al.^341^ detected 231 DEcircRNAs, including 121 with upregulation whereas 110 with downregulation. circRNA_0000660 expression is significantly related to Igfbp1 expression, while circRNA_0000660 silencing decreases liver lipid accumulation. Maternal obesity induces metabolic disorders in offspring by changing liver circRNA expression. CircHIPK3 modulates lipid metabolism within hepatocytes. It promotes triglyceride level and adipose deposition within HepG2 cells, and induces diabetes‐associated metabolic diseases like insulin resistance and hyperglycemia by decreasing miR‐192‐5p and increasing its downstream TF FOXO1.^233^

**FIGURE 5:**
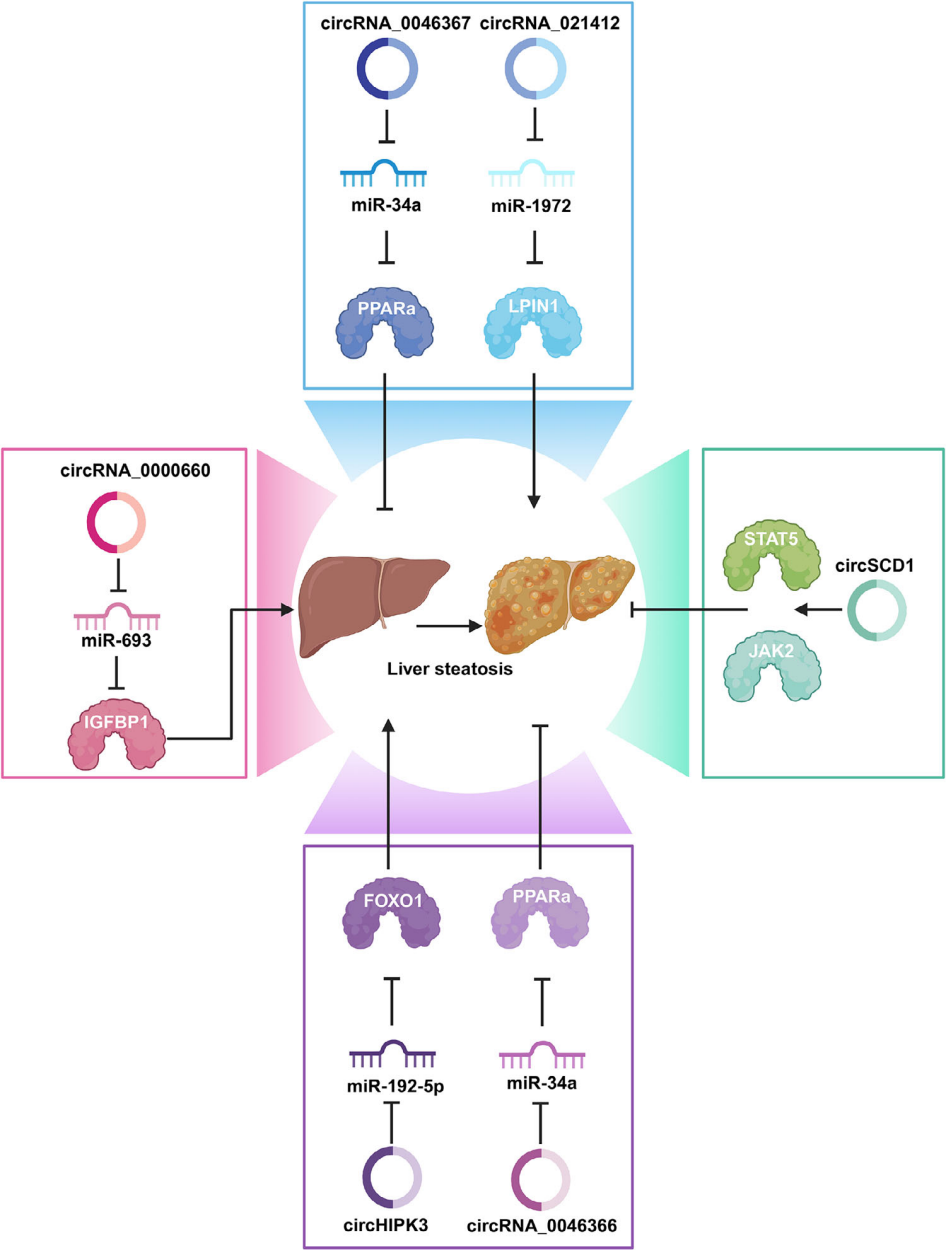
CircRNAs in the regulation of hepatic steatosis. This figure highlights the roles of circRNAs in hepatic steatosis. CircRNA_0046366 and circRNA_0046367 reduce hepatic steatosis by competing with PPARα for miR‐34a binding. In NAFLD, abnormal circSCD1 expression promotes fatty liver disease through the JAK2/STAT5 pathway.

CircRNAs are widely suggested with vital effects on regulating hepatic steatosis^355^, ^356^ (Figure 5). miR‐34a can bind to PPARα in rodents, which suppresses lipid metabolism and enhances hepatic steatosis, while circRNA_0046366 and circRNA_0046367 compete against PPARα for binding to miR‐34a. These circRNAs act as endogenous regulators of miR‐34a, reducing hepatic steatosis by suppressing the interaction of miR‐34a with MRE within PPARα mRNA. Aberrant circRNA_0046366 and circRNA_0046367/miR‐34a/PPARα signaling is the potential novel target for treating hepatic steatosis.^342^, ^343^ In high‐fat‐diet‐mediated hepatic steatosis, circRNA_021412 expression significantly decreases.^344^ circRNA_021412 downregulation suppresses the competitive inhibition on miR‐1972, therefore repressing Lpin1, the target gene of miR‐1972. Lpin1 downregulation can downregulate long‐chain acyl CoA synthase, causing hepatic steatosis.

NAFLD is the condition of excess fat accumulation within hepatocytes not associated with alcohol abuse. Many NAFLD patients experience nonalcoholic steatohepatitis (NASH).^357^ Aberrant circSCD1 expression influences hepatocyte lipidation and enhances fatty liver disease via JAK2/STAT5 pathway.^345^ Jin et al.^353^ established four circRNA–miRNA–mRNA pathways using the NASH mouse model. Their results showed that circRNA expression patterns might be employed to diagnose NASH.

### 3.3 CircRNAs in CVD

**FIGURE 6:**
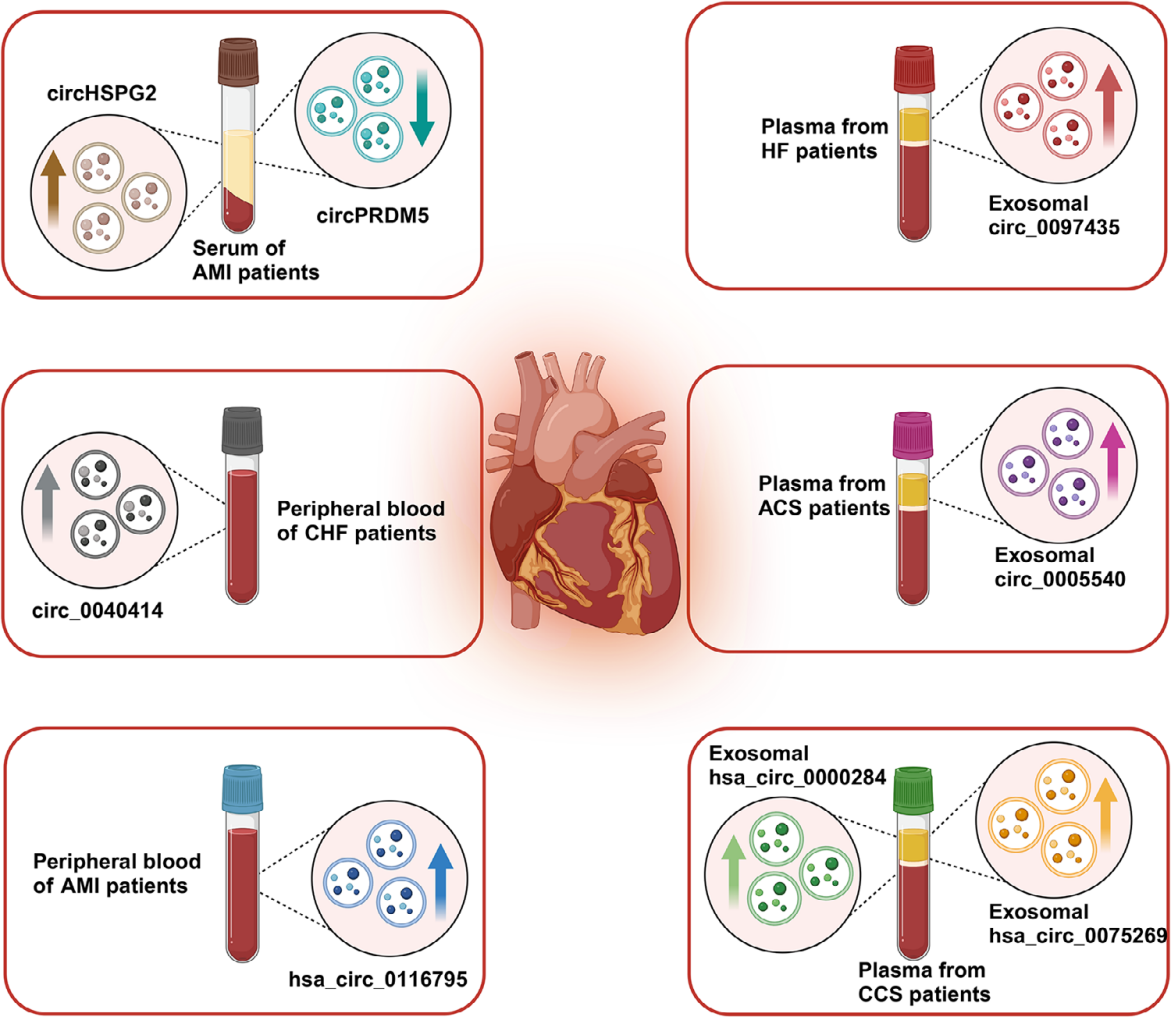
CircRNAs in cardiovascular disease. This figure showcases the diverse applications of circRNAs in blood samples from patients with various cardiac conditions. The top left displays plasma containing circHSPG2, associated with AMI patients. Adjacent is plasma from AMI patients featuring circPRDM5, emphasizing its potential role. The top right depicts exosomal circ_0097435 extracted from plasma of HF patients. Moving downward, the middle right panel presents plasma from ACS patients, containing exosomal circ_0005540. The middle left showcases plasma from CHF patients with circ_0040414. The bottom left highlights peripheral blood of AMI patients with hsa_circ_0116795, while the bottom right displays plasma from CCS patients, featuring exosomal hsa_circ_0000284 and circ_0075269. These findings underscore the significance of circRNAs in diagnosing and understanding the pathogenesis of cardiac diseases. (CircRNAs, circulating microRNAs; AMI: acute myocardial infarction; HF: hemodialysis; ACS, acute coronary syndrome; CHF, chronic heart failure; CCS, coronary chronic stable).

CVD is a group of disorders that affect the heart and cardiovascular system,^358^ including AS, angina, myocardial infarction (MI), stroke,^359^ HF, heart valve disease, and cardiomyopathy.^360^ CVDs are among the leading causes of disability and death worldwide.^361^, ^362^ They can be caused by various risk factors, including diabetes, smoking, advanced age, dyslipidemia, and hypertension.^363^ In the field of CVD research and treatment, exosomes play a crucial role, especially as biomarkers of disease progression and treatment response, reflecting various cardiovascular conditions such as coronary heart disease (CHD), AS, acute myocardial infarction (AMI), and HF.^364^, ^365^, ^366^, ^367^ Exosomes, as mediators of cell‐to‐cell communication, have emerged as important regulators of angiogenesis and cardiac repair.^368^, ^369^ Additionally, exosome‐derived circRNAs play a significant role in the pathophysiology of the cardiovascular system by adsorbing miRNAs and regulating protein production, thereby participating in the progression of CVDs. These findings provide new insights into the complex mechanisms of CVDs and offer an important theoretical basis for developing new diagnostic tools and therapeutics (Figure 6).

#### 3.3.1 CircRNAs in CHD

CHD is the most common heart disease worldwide. According to the World Health Organization, approximately 7 million people die from CHD every year.^370^ AS is a chronic inflammatory disease and the main pathological basis of CHD. The accumulation of lipid, calcium, and fibrous tissue in the inner wall of the artery increases the thickness and decreases the elasticity, leading to hardening, narrowing, and potential occlusion of the blood vessels.^371^ AS mainly occurs in large and medium‐sized arteries, such as the coronary artery, carotid artery, abdominal aorta, and femoral artery.^372^ It is also the root cause of stroke and MI.^373^ Many studies have shown that exosome‐derived circRNA is often regarded as a reliable biomarker reflecting the disease status of CHD due to its stability in body fluids and long half‐life. Vilades et al.^374^ found that the level of hsa_circ_0001445 was closely related to the degree of coronary AS in CHD patients and could improve the accuracy of cardiovascular risk assessment models. The Wang's team found that hsa_circ_0001879 and hsa_circ_0004104 were significantly upregulated in CHD patients, providing a new avenue for early diagnosis of the disease.^375^ The study by Wu et al.^376^ further revealed 355 DEcircRNAs in CHD patients, especially hsa_circ_0005540, which was significantly associated with CHD (*p* < 0.0001) and showed high discrimination power in ROC analysis (AUC = 0.853). Miao et al.^377^ also pointed out that circ‐YOD1, hsa‐miR‐21‐3p, and hsa‐miR‐296‐3p have important diagnostic value in CHD, with circ‐YOD1 showing high prognostic value. The study by Xiong et al.^378^ found that the expression level of CircNPHP4 derived from monocyte exosomes was significantly increased in CHD patients. This circRNA showed a high degree of discrimination and could effectively distinguish CHD patients from nonpatients.^378^ Liu et al.^379^ confirmed that circRNAs carried by plasma exosomes showed significant differential expression in chronic coronary syndrome (CCS), with exo‐hsa_circ_0075269 and exo‐hsa_circ_0000284 being significantly upregulated in CCS patients.^379^ Additionally, Liu et al. found that exosome‐derived circ_0026218 was involved in the development of AS by affecting miR‐188‐3p and regulating the TLR4/NF‐κB pathway.^380^ Taken together, these studies demonstrate the great potential of circRNAs in the diagnosis of CHD, providing new molecular markers for early diagnosis and possible new targets for the treatment and prognosis of the disease.

#### 3.3.2 CircRNAs in acute coronary syndrome

Acute coronary syndrome (ACS) is a sudden occurrence of CAD, mainly consisting of ST‐segment elevation myocardial infarction (STEMI), non‐ST‐segment elevation myocardial infarction (NSTEMI), and unstable angina.^381^ ACS is usually caused by plaque rupture in AS and subsequent thrombus formation.^382^ Studies have shown that circRNA plays a regulatory role in MI models.

##### Role as biomarkers

Huang et al.^383^ found that hsa_circ_0116795 was significantly upregulated in the peripheral blood of AMI patients, and its expression increased in cardiomyocytes under hypoxic conditions, suggesting it may be used as a biomarker for early diagnosis of AMI. Liu et al.’s study^384^ indicated that the expression of circPRDM5 in the serum of AMI patients was significantly reduced. The sensitivity of AMI diagnosis could be significantly improved when circPRDM5 was combined with cardiac troponin T and creatine kinase‐MB.^384^ Tian et al.,^385^ while exploring biomarkers of sudden cardiac death (SCD) caused by acute ischemic heart disease (IHD), found time‐dependent expression patterns of circSLC8A1 and circNFIX in animal and cellular models that matched findings from autopsy cases. This suggests their potential value in distinguishing IHD‐related SCD. In particular, the high sensitivity and specificity of circSLC8A1, as well as its positive correlation with creatine kinase‐MB in pericardial fluid, and downregulated circNFIX levels may reflect ischemic myocardial injury, which is negatively correlated with the degree of coronary artery stenosis.^385^ The study by Tong et al.^386^ analyzed microarray data of exocircRNA and mRNA in AMI from CHD patients. The constructed ceRNA network analysis revealed specific circRNAs (such as circRNA0001785, circRNA0000973, circRNA0001741, and circRNA0003922) as promising candidates for effective prediction of CHD.^386^

##### CircRNA promotes the progress of ACS

MI and I/R injury are the most common and fatal manifestations of ACS. Increasing evidence shows that circRNAs play an important role in the occurrence and development of ACS.

First, circRNAs affect cardiac fibrosis after MI by regulating the proliferation and migration of CFs. Pang et al.^387^ showed that circHelz can bind to Yes‐associated protein (YAP) and promote its localization to the nucleus, thereby promoting cell proliferation and growth. Reducing circHelz expression alleviates cardiac fibrosis and the activation of CFs.^387^ Wang et al.^388^ pointed out that exosome circUbe3a released by M2‐type macrophages promotes the proliferation and migration of CFs by adsorbing miR‐138‐5p and inhibiting the translation of Rho family GTPase 3 (RhoC). Deng et al.^389^ revealed that circ‐HIPK3 interacts with miR‐17‐3p and adenylate cyclase type 6 (ADCY6) to regulate calcium ion concentration in cardiomyocytes and affect cardiac function. Reducing its level can reduce cardiac fibrosis and maintain cardiac function after MI.^389^ These studies suggest that regulating the expression of specific circRNAs may effectively reduce cardiac fibrosis after MI, providing a new strategy for the treatment of CVDs.

Second, circRNAs affect MI and I/R injury by regulating inflammatory responses and immunomodulatory processes. For example, Huang et al. found that circ_SMG6 binds to miR‐138‐5p, upregulates early growth response 1 (EGR1) expression, promotes neutrophil recruitment, and exacerbates myocardial injury.^390^ Additionally, Zhu et al.^391^ showed that the expression of circITGB1 was increased in the plasma of AMI patients, which increased the expression of NFAT activating molecule 1 (NFAM1) by competitively binding to miR‐342‐3p and affected the maturation of DCs, highlighting the role of circRNA in inflammatory response and immune regulation. Ye et al.^392^ pointed out that the serum concentrations of IL‐1β and IL‐18 were increased, circ‐NNT and ubiquitin specific peptidase 46 (USP46) were upregulated, while miR‐33a‐5p was downregulated in MI patients. By regulating USP46, circ‐NNT promoted pyroptosis in myocardial I/R injury.^392^

Notably, circRNAs also act by affecting specific signaling pathways. Zhang et al.^393^ found that circ_0030235 activated the PI3K/AKT and MEK/ERK signaling pathways by regulating miR‐526b to promote injury in MI models using an oxygen glucose deprivation/reperfusion (OGD/R) model. Zhu et al.^394^ found that circMAT2B increased OGD‐induced cell damage and inflammatory damage by positively regulating miR‐133 and activating the PI3K/AKT and Raf/MEK/ERK signaling pathways in MI. Li et al.^395^ found that inhibition of circARPA1 could effectively reduce myocardial cell fibrosis and apoptosis, emphasizing the importance of the Wnt/β‐catenin signaling pathway in this process.

In addition, numerous studies have revealed that circRNAs aggravate myocardial injury. For example, circROBO2 can act as a sponge for miR‐1184, promoting cardiomyocyte apoptosis by upregulating the expression of TNF receptor associated death domain.^396^ Circ_0049271 aggravated myocardial cell damage through the miR‐17‐3p/FZD4 signaling axis in hypoxia‐reoxygenation induced myocardial cell damage.^397^ The expression of circHSPG2 and MAPK kinase kinase 2 (MAP3K2) is increased in the serum of AMI patients, while the expression of miR‐1184 is decreased. Downregulation of circHSPG2 can reduce myocardial cell injury.^398^ Circ_0023461 is upregulated under hypoxic conditions and upregulates the expression of phosphodiesterase 4D (PDE4D) by acting on miR‐370‐3p, thereby regulating the viability and glycolytic ability of AC16 cardiomyocytes.^399^ CircRbms1 promotes myocardial cell injury by regulating the miR‐742‐3p/forkhead box O1 (FOXO1) axis in the progression of MI.^400^ Circ_0124644 increases myocardial cell injury in AMI by targeting miR‐590‐3p and affecting the expression of SRY‐box TF 4.^401^ Circ_0007059 is upregulated during MI and promotes inflammation and cardiomyocyte apoptosis by acting as a sponge for miR‐378 and miR‐383.^402^ Under hypoxia/reoxygenation (H/R) conditions, the expression of circ‐CBFB in cardiomyocytes increases, promoting apoptosis by adsorbing miR‐495‐3p, revealing the role of circ‐CBFB in H/R injury.^403^

In summary, circRNAs play a key role in cardiac fibrosis, myocardial apoptosis, inflammatory response, and myocardial I/R injury after MI by regulating the proliferation, migration, myocardial apoptosis, inflammatory response, and immunomodulatory processes of CFs, as well as specific signaling pathways. These studies not only deepen our understanding of the pathogenesis of CVDs but also provide important clues for the development of new therapeutic strategies.

##### CircRNA inhibits the progress of ACS

By acting as a “molecular sponge” for miRNA, circRNAs can affect the activity of miRNA and regulate the expression of downstream target genes. This mechanism also plays a key inhibitory role in the occurrence and development of ACS.

CircRNAs play an important role in alleviating myocardial hypoxia. For example, Zhao et al.^404^ revealed the protective effect of circHSPG2 on cardiomyocytes under hypoxic conditions. By acting as a molecular sponge for miR‐25‐3p, circHSPG2 positively regulates the miR‐25‐3p/proapoptotic WT1 regulator axis and protects cardiomyocytes from hypoxia‐induced damage.^404^ Similarly, Zhou et al.^405^ found that circ_0001747 in ADSC exosomes alleviated I/R injury in cardiomyocytes by targeting miR‐199b‐3p. Gao et al.^406^ found that H/R‐induced cardiomyocyte death could be alleviated by enhancing the expression of mmu_circ_000338 in H/R‐treated and I/R‐injured mouse hearts. By directly binding to histone deacetylase 7 (HDAC7) and interfering with its entry into the nucleus, mmu_circ_000338 alleviated the inhibition of HDAC7 on forkhead box protein A2 (FOXA2) transcription, thereby upregulating FOXA2 and inhibiting receptor‐interacting protein kinase 3‐dependent necrosis.^406^ Zhao et al.^407^ investigated hypoxic conditions in vitro and found that circRNA_010567 enhanced mitochondrial activity by adsorbing miR‐141 and increasing death‐associated protein kinase 1 expression, thus playing a protective role in hypoxia‐induced myocardial cell damage. Liu et al.^408^ revealed the protective role of circ_0002612 in I/R injury. Circ_0002612 releases the expression of its target peroxisome proliferator‐activated receptor gamma coactivator 1 alpha (Ppargc1a) by competitively binding to miR‐30a‐5p, thereby inhibiting the expression of NLRP3 and contributing to the survival of cardiomyocytes and reducing apoptosis.^408^

In addition, circRNAs are also involved in the process of cardiac repair and remodeling. These studies reveal the important role of circRNA in the pathological process of the heart and provide a new target for the treatment of heart diseases. For example, Wang et al.^409^ found that circHIPK3 could promote cardiac endothelial cell cycle progression and proliferation by reducing miR‐29a activity and increasing vascular endothelial growth factor A (VEGFA) expression, thus alleviating damage after MI. Garikipati et al.^410^ showed that overexpression of circFndc3b, which was downregulated after MI, promoted angiogenesis, reduced cardiomyocyte apoptosis, and improved cardiac function. Ju et al.^411^ found that overexpression of circRNA FEACR could inhibit MI and improve cardiac function by directly binding to nicotinamide phosphoribosyltransferase (NAMPT), improving NAMPT protein stability, enhancing SIRT1 expression, and promoting forkhead box protein O1 (FOXO1) transcriptional activity, thereby upregulating the transcription of the ferroptosis inhibitor Fth1. Li et al.^412^ revealed that regulating the circCELF1/miR‐636/Dickkopf WNT signaling pathway inhibitor 2 (DKK2) pathway can reduce myocardial fibrosis and improve cardiac function. Zhu et al.^413^ showed that circNFIB expression was reduced after MI, and its overexpression could reduce the proliferation of CFs by adsorbing miR‐433. Zhou et al.^414^ found that circRNA ACR activated (Pink1) expression and reduced myocardial infarct size by blocking DNA methyltransferase 3B (DNMT3B)‐mediated DNA methylation of the Pink1 promoter. Sun et al.^415^ explored the role of circFoxo3 in MI and found that overexpression of circFoxo3 improved cardiac dysfunction caused by MI. Wang et al.^416^ focused on the role of mitochondrial fission and apoptosis‐related circRNA (MFACR) in cardiac cell death and pointed out that MFACR prevented mitochondrial protein 18 kDa (MTP18) translation by inhibiting miR‐652‐3p and reducing cardiac cell death. Zhao et al.^417^ found that circMACF1 could act as a “sponge” of miR‐500b‐5p to upregulate the expression of EMP1 and slow down the progression of AMI. Notably, Wei et al.^418^ found that circWhsc1 in EVs produced by endothelial cells under hypoxic conditions promoted cardiomyocyte proliferation and cardiac regeneration. This process enhances tripartite motif containing 59 (TRIM59) phosphorylation, thereby increasing TRIM59 binding to signal transducer and activator of transcription 3 (STAT3) and promoting STAT3 phosphorylation, which ultimately leads to cardiomyocyte proliferation, potentially providing a therapeutic target for cardiac repair after MI.^418^ Yu et al.^419^ showed that circCEBPZOS levels were reduced in the serum of patients with poor cardiac remodeling. Overexpression of circCEBPZOS can improve cardiac function by regulating the miR‐1178‐3p/phosphoinositide‐dependent kinase‐1 (PDPK1) axis, thereby reducing adverse remodeling and promoting angiogenesis after MI.

In terms of enhancing the antioxidant capacity of cardiomyocytes, studies have shown that circRNAs are involved in regulation through different mechanisms. For example, Fan et al.^420^ found that H_2_O_2_‐induced cardiomyocyte injury could be alleviated by increasing circ_HIPK3 expression. The mechanism is that circ_HIPK3 upregulates the expression of its target IRS1 by adsorbing miR‐33a‐5p, thereby improving cell survival.^420^ Chen et al.^421^ found that elevated circ‐SWT1 expression attenuated H_2_O_2_‐induced cardiomyocyte injury in an AMI model. Circ‐SWT1 interacts with miR‐192‐5p to reduce the inhibition of SOD2 by miR‐192‐5p, thereby enhancing the antioxidant capacity of cells.^421^

These research results demonstrate the multifaceted role of circRNAs in MI and its subsequent cardiac pathological processes, involving not only the protection and repair of cardiomyocytes but also the regulation of cardiomyocyte death. These findings advance our understanding of the molecular mechanisms of CVDs and provide possibilities for developing new therapeutic strategies.

#### 3.3.3 CircRNAs in HF

CircRNAs have potential as markers in the diagnosis of chronic heart failure (CHF). Feng et al.^422^ found that circ_0040414 was significantly upregulated in CHF patients and inhibited AKT signaling pathway activity by promoting apoptosis and inflammatory response, suggesting its possibility as a biomarker of CHF. Sun et al.,^423^ through the analysis of circRNA in the plasma of HF patients, specifically pointed out that hsa_circ_0062960 was highly correlated with the serum level of B‐type natriuretic peptide, which had high diagnostic value. Therefore, circRNAs have great potential as diagnostic markers of HF and can provide a new direction for the early diagnosis and treatment of HF.

In recent years, remarkable progress has been made in understanding the molecular mechanisms of HF, especially in the mechanism of circRNA action. Han et al.^424^ identified 56 DEcircRNAs in the peripheral blood samples of HF patients and healthy volunteers through next‐generation sequencing technology, highlighting the possible important role of hsa_circ_0097435 in HF, which may participate in the regulation of myocardial cell damage by absorbing multiple miRNAs. Guo et al.^425^ pointed out that circ_0036176 reduced the proliferation of CFs by inhibiting the CDK/retinoblastoma protein (Rb) pathway by encoding the Myo9a‐208 protein, and the intervention of miR‐218‐5p could relieve its inhibitory effect on the proliferation of CFs. Zhu et al.,^426^ by studying the role of circRNA in hypertrophic preconditioning (EHP), identified a novel circRNA, c‐Ddx, which was involved in the antihypertrophic memory of EHP by binding to and activating eukaryotic elongation factor 2. Wang et al.^427^ found that increased expression of circSnap47 in the HF condition resulted in decreased cardiomyocyte viability and increased apoptosis. By silencing circSnap47 and regulating its downstream target gene miR‐223‐3p and the MAPK pathway signaling pathway, the survival rate of cardiomyocytes can be improved, and cell apoptosis can be reduced, thus inhibiting the progression of HF.^427^ These studies not only reveal the potential role of circRNA in HF and cardiac hypertrophy but also provide important clues for developing new therapeutic strategies.

In addition, circRNAs also have a protective effect on HF. Yan et al.^428^ found that the endogenous proliferation ability of cardiomyocytes was activated after surgical ventricular reconstruction (SVR), and circRNAs played an important role in this process. By performing SVR surgery on mice with MI, they found that the upregulation of circMap4k2 expression was correlated with cardiomyocyte proliferation. Overexpression of circMap4k2 significantly promoted the proliferation of cardiomyocytes, while silencing circMap4k2 inhibited the proliferation. Further studies found that circMap4k2 affects antizyme inhibitor 1 (Azin1), a key factor in cardiomyocyte proliferation, by binding to miR‐106a‐3p, thereby reducing cardiac remodeling after SVR.^428^ Hu et al.^429^ then explored the core circRNA signaling pathways related to HF from the global triple network of miRNA, circRNA, and mRNA. They found that the downregulation of circRNA CHRC was associated with cardiac hypertrophy, and it affected the progression of cardiac hypertrophy and HF by interacting with miR‐431‐5p to regulate the key TF Krüppel‐like factor 15 (KLF15). This finding provides a new target for the treatment of HF.^429^ Li et al.^430^ showed that statins could upregulate the expression of circRNA–RBCK1 and promote its interaction with miR‐133a, which involves the direct interaction between TF activator protein 2 alpha and ring‐B‐box‐coiled‐coil protein interacting with protein kinase C‐1 (RBCK1). Through this signaling pathway, statins can improve cardiac diastolic function in patients with HF.^430^ Wang et al.^431^ investigated the role of a specific circRNA HRCR in cardiac hypertrophy and HF and found that HRCR could act as a sponge for miR‐223 and prevent the development of cardiac hypertrophy. They further found that the overexpression of miR‐223 led to cardiac hypertrophy, and HRCR increased the expression of the antiapoptotic protein ARC, an inhibitor of apoptosis, by sequestering miR‐223, thereby reducing the cardiac hypertrophy response.^431^ Zuo et al.^432^ investigated the role of endothelial progenitor cell‐derived exosomal circ_0018553 during cardiac hypertrophy and found that this circRNA plays an important role in Ang II‐induced cardiac hypertrophy. Circ_0018553 affects the expression of sirtuin 2 (SIRT2) by acting as a sponge for miR‐4731, thereby inhibiting the process of myocardial hypertrophy.^432^

Taken together, these studies reveal the complex regulatory roles of circRNA in the process of HF pathology. Through different mechanisms, including directly affecting cardiomyocyte proliferation, regulating the activity of specific miRNAs, participating in signaling pathways, and interacting with drugs, circRNAs play key regulatory roles in HF.

#### 3.3.4 CircRNAs in other CVDs

In other CVDs, circRNAs also play an important role. Zhu et al.^433^ identified a large number of DEcircRNAs in patients with valvular heart disease accompanied by atrial fibrillation (AF), suggesting that cAMP and Wnt signaling pathways may play a key role in the pathological process. These pathways, regulated by DEcircRNAs, may alter the electrophysiological properties and systolic function of the heart by affecting gene expression and protein synthesis in cardiomyocytes.^433^ Wu et al.’s^434^ study further explored the specific role of circRNA in AF and found that mmu_circ_0005019 regulated the expression of potassium channel SK3 (KCNN3) by acting as a sponge for miR‐499‐5p, thereby affecting the expression of key ion channel proteins in cardiac electrophysiology. Additionally, Li et al.^435^ showed that circRNA secreted by MSC‐derived exosomes can reduce oxidative stress in cardiomyocytes and improve cell survival rates in myocardial injury caused by sepsis by regulating the miR‐497‐5p/MG53 axis, demonstrating potential therapeutic value. These studies not only reveal the complex regulatory network of circRNA in CVDs but also provide important clues for developing new therapeutic strategies.

### 3.4 CircRNAs in kidney disease

Kidney disease is a global public health problem affecting hundreds of millions of people worldwide.^436^, ^437^ It includes various types such as chronic kidney disease (CKD), acute kidney injury (AKI), and nephritis. CKD is of particular interest due to its high incidence and close association with other major health problems like CVD and diabetes.^438^ Recently, an increasing number of studies have focused on the effects of exosome‐derived circRNAs on AKI, DN, nephrotic syndrome, and renal cancer. These circRNAs have the potential to become new diagnostic markers and therapeutic targets, bringing new hope for the diagnosis and treatment of kidney diseases.

#### 3.4.1 Acute kidney injury

CircRNAs play an important regulatory role in the progression of AKI. The study by Kuang et al.^439^ revealed that circ_0001818 exacerbates sepsis‐induced AKI by adsorbing miR‐136‐5p, thereby increasing the expression of thioredoxin interacting protein and aggravating cell death and inflammation. Li et al.^440^ found that circ‐FANCA also plays a key role in sepsis‐induced AKI by influencing the interaction between miR‐93‐5p and oxidative stress responsive 1 to regulate cell survival and apoptosis. Additionally, using the exosome inhibitor GW4869, the level of circ‐FANCA released by exosomes under septic conditions can be reduced, thereby decreasing cell damage.^440^ Kölling et al.^441^ investigated the role of circRNAs in acute renal rejection and found that the concentrations of hsa_circ_0001334 and hsa_circ_0071475 were significantly increased in the urine of patients with acute renal rejection. The abnormal expression of these circRNAs not only plays an important role in diagnosis but can also serve as an indicator of treatment response. Moreover, there was a correlation between hsa_circ_0001334 and a decrease in glomerular filtration rate 1 year after transplantation, suggesting it may be associated with the deterioration of renal function after acute rejection.^441^ Yang et al.^442^ investigated the protective effect of human urine‐derived stem cell‐derived exosomes (USC‐Exos) in AKI. USC‐Exos affected the interaction between miR‐138‐5p and FOXO3a by increasing the expression of circDENND4C. Through this mechanism, USC‐Exos promote cell proliferation and inhibit the activation of NLRP3, which helps reduce pyroptosis and inflammation, ultimately alleviating AKI.^442^

Taken together, these studies highlight the different roles of circRNAs in the development and progression of AKI, and further research will help to understand the mechanisms of AKI and identify appropriate therapeutic targets.

#### 3.4.2 Nephrotic syndrome

Nephrotic syndrome refers to a group of clinical symptoms and signs caused by abnormal glomerular permeability, including massive proteinuria, hypoproteinemia, hyperlipidemia, and edema.^443^, ^444^ Primary nephrotic syndrome, which is not caused by external diseases, results from conditions that directly affect the kidneys. Common forms of primary nephrotic syndrome include minimal change disease, membranous nephropathy (MN), and focal segmental glomerulosclerosis (FSGS).^445^ In contrast, secondary nephrotic syndrome arises from systemic diseases or other conditions that damage the kidneys, such as lupus nephritis (LN).^446^ Recently, the unique function of circRNA in nephrotic syndrome has garnered significant attention. The differential expression and regulatory mechanisms of circRNA in pathological states, including MN and FSGS, highlight its crucial role in the development and progression of nephrotic syndrome.

#### 3.4.3 Lupus nephritis

LN is a severe complication of systemic lupus erythematosus (SLE) that affects the kidneys. SLE is an autoimmune disease where the body's immune system mistakenly attacks and destroys healthy tissues, leading to inflammation and kidney problems.^447^ SLE is more common in women, particularly those of reproductive age, and is also more prevalent in certain racial and ethnic groups, such as African Americans, Asians, and Hispanics.^448^, ^449^ Approximately 40–70% of patients with SLE develop LN.^450^ Recent studies have highlighted the complex role of circRNA in the pathological mechanisms of LN, revealing both its promotive and inhibitory effects on disease progression. For instance, Miao et al.^451^ discovered that circRTN4 is upregulated in MCs in LN and transferred from monocytes to MCs via exosomes. CircRTN4 interacts with miR‐513a‐5p, enhancing fibronectin expression, promoting cell proliferation, and ECM deposition, thereby exacerbating kidney injury. This suggests that circRTN4 upregulation contributes to the pathological process of LN.^451^ Ouyang et al.^452^ found significantly higher levels of circRNA_002453 in the plasma of LN patients compared with a control group. The expression level of circRNA_002453 was positively correlated with disease severity indicators, indicating its potential as a biomarker for LN diagnosis.^452^ However, some studies have shown the opposite. Zhang et al.^453^ showed that the expression of hsa_circ_0123190 is decreased in LN patients, leading to increased activity of its adsorbed hsa‐miR‐483‐3p. This interaction affects the expression of the apelin receptor (APLNR) gene, which is related to renal fibrosis, highlighting the critical role of hsa_circ_0123190 in inhibiting LN pathogenesis.^453^ Additionally, Luan et al.^454^ reported that circMTND5, located in the mitochondrial genome, is significantly downregulated in renal biopsy samples of LN. This downregulation is associated with mitochondrial dysfunction, decreased mitochondrial gene expression, and increased profibrotic gene expression, promoting renal fibrosis. CircMTND5 mitigates mitochondrial damage and renal fibrosis by adsorbing miR6812, suggesting that its downregulation adversely affects LN, while increased expression may provide renal protection.^454^

These studies collectively demonstrate the complex role of circRNAs in LN, showing that specific circRNA–miRNA interactions can either promote or inhibit disease progression. This provides crucial molecular targets for developing new therapeutic strategies.

#### 3.4.4 Others

First, the study by Li et al.^455^ revealed that the expression of numerous circRNAs is altered in patients with idiopathic membranous nephropathy (IMN). Notably, the significant upregulation of hsa_circ_0001250 is closely related to the high levels of proteinuria characteristic of IMN. This finding suggests that hsa_circ_0001250 is involved in the pathological process of IMN by targeting hsa‐miR‐639 and hsa‐miR‐4449, thereby regulating the expression of specific genes. This provides new insights for the development of noninvasive diagnostic methods.^455^ Second, the work of Sun et al.^456^ found that circ_0000524 affects podocyte apoptosis through its interaction with miR‐500a‐5p and CXC motif chemokine ligand 16 (CXCL16), which is key to the pathogenesis of MN. This study not only reveals the importance of podocyte apoptosis in MN but also suggests a new treatment strategy by interfering with the circ_0000524/miR‐500a‐5p/CXCL16 signaling pathway.^456^ Additionally, Qiu et al.^457^ showed that circ_CDYL expression is significantly increased in MN and affects podocyte apoptosis by regulating the expression of miR‐149‐5p and TNF superfamily member 11. This finding highlights the critical role of podocytes in renal filtration function and suggests that circ_CDYL may become a new target for treating MN.^457^ Finally, the study by Cui et al.^458^ focused on FSGS and found that circZNF609 is upregulated during disease development. It affects podocyte injury and renal fibrosis by regulating the expression of miR‐615‐5p. This study illuminates the molecular mechanisms of FSGS and provides directions for developing new treatment strategies.^458^

CircRNAs play diverse and complex roles in nephrotic syndrome. By interacting with miRNAs, circRNAs can affect key pathological processes such as podocyte apoptosis and proteinuria, thus playing a central role in the occurrence and development of nephrotic syndrome.

### 3.5 CircRNAs in musculoskeletal and connective tissue diseases

#### 3.5.1 CircRNAs in RA

Inflammatory RA causes joint deformities and complete impairment of joint function. There are abnormalities in mast cells, B cells, macrophages, DCs, plasma cells, and T cells involved in RA pathogenesis, as well as the functional behavior of fibroblast‐like synoviocytes (FLSs).^459^

CircRNAs and their sponge effect on miRNAs contribute to the pathogenesis of RA by regulating target genes. Many circRNAs, such as circRNA_092516, circRNA_003524, circRNA_103047, and circRNA_101873, are documented to be aberrantly expressed in RA.^460^ CircRNAs in exosomes and RNA methylation are closely related to RA pathogenesis.^461^ Additionally, circulating RNAs play a role in RA's pathological mechanisms in PBMCs and macrophages. Ouyang et al.^462^ identified and validated five circRNAs (092516, 003524, 103047, 104871, 101873) significantly elevated in PBMCs of RA patients. The circ_0008360 gene has emerged as a pivotal regulator of inflammation, with its dysregulation considered highly significant in RA.^463^ Geng and colleagues^464^ investigated the functional consequences of circ_0088036 in the advancement of RA and revealed the mechanisms behind it. Circ_0088036 contributes to the activity of RA induced by TNF‐α, partly through the miR‐326/FZD4 signaling pathway.^464^ Additionally, circ_0088036 promotes synovial pathogenesis through the circ_0088036/miR‐140‐3p/SIRT1 axis in RA.^465^ Circ_0000479 facilitates RA development by interacting with miR‐766, leading to increased FKBP5 expression.^466^ Yang et al.^467^ discovered that circRNA_17725 promotes M2 polarization of macrophages to alleviate arthritis symptoms. Wang et al.^468^ demonstrated that blocking the circPTN/miR‐145‐5p/FZD4 signaling axis could effectively inhibit RA pathology.

CircRNAs affect the proliferation, migration, and inflammatory reactions of RA‐FLSs. These interactions form circRNA–miRNA axes that participate in various biological activities within RA‐FLSs. FLSs are mesenchymal‐origin cells crucial for the synovium's physiological functions, contributing to synthesizing specific constituents in synovial fluid and articular cartilage. Recent investigations have revealed that circRNAs contribute to various FLS activities, including metastasis, proliferation, and cytokine production.^469^ Circ_0001947 is involved in RA pathogenesis by enhancing STAT3 expression through sequestering miR‐671‐5p.^470^ Circ_0002984 regulates RA‐FLS activity by interacting with miR‐543, leading to upregulated PCSK6 expression.^471^ CircAFF2 promotes RA initiation and advancement by modulating the miR‐650/CNP axis, enhancing proliferation, inflammatory response, migration, and invasion of RA‐FLSs.^472^ CircMAPK9 affects RA‐FLS activity by modulating the miR‐140‐3p/PPM1A axis.^473^ CircSirt1's protective mechanism on FLS involves impeding cell proliferation, fostering apoptosis, and facilitating the miR‐132‐mediated Sirt1 pathway, thus reducing inflammation.^474^ Inhibition of circ_0004712 suppresses aggressive phenotypic changes in RA‐FLSs by modulating the miR‐633/TRAF6 axis and NF‐κB signaling.^475^ Circ_0025908 silencing alleviates dysfunctions in RA‐FLSs by targeting the miR‐650/SCUBE2 axis.^476^ Circ_0007707 modulates inflammatory response and cell apoptosis in RA‐FLSs via the miR‐27b‐3p/PDE3B axis, highlighting its potential as a therapeutic target for RA.^477^ Circ_0088194 affects RA‐FLSs through the miR‐30a‐3p/ADAM10 axis, promoting inflammation, migration, proliferation, and inhibiting apoptosis.^478^ Silencing circ‐PTTG1IP represses cell proliferation, migration, and inflammatory response in RA‐FLSs by modulating the miR‐671‐5p/TLR4 axis.^479^ Inhibiting circCDKN2B‐AS_006 mitigates arthritis severity by suppressing tumor‐like growth and metastasis in RA‐FLSs.^480^

CircRNAs are critical for RA progression, as they are released by FLSs in RA (RA‐FLSs‐Exos). The proliferation and migration of RA‐FLSs‐derived exosomes overexpressing circFTO is inhibited, which promotes the modification of chondrocytes by m6A, ultimately resulting in RA by upregulating the sex‐determining region Y‐related high‐mobility group box 9 (SOX9).^481^ A microarray analysis of RA revealed a distinct profile of circRNA expression.^460^ The overexpression of CircEDIL3 leads to an upregulation of PIAS3 protein expression, which in turn inactivates STAT3, thereby alleviating the progression of RA. Moreover, CircEDIL3 originating from SMSCs‐Exos effectively suppresses inflammation‐induced angiogenesis and promotes pannus progression, both in vitro and in vivo, via the miR‐485‐3p/PIAS3/STAT3/VEGF functional module, ultimately ameliorating RA.^482^ CircFBXW7, delivered by exosomes from MSCs to RA‐FLSs, suppresses cell proliferation, migration, invasion, and inflammatory cytokines like TNF‐α, IL‐1, IL‐6, and IL‐8 via the circFBXW7/miR‐216a‐3p/HDAC4 axis.^483^

CircRNA expression levels are tissue‐ and stage‐specific, with many circRNAs found in human peripheral blood, suggesting their potential as biomarkers for RA screening, diagnosis, characterization, and monitoring. Investigating novel diagnostic and therapeutic strategies targeting circRNAs is promising. Due to their unique expression characteristics and pivotal role in RA, circRNAs hold promise as diagnostic biomarkers and therapeutic targets.^460^

#### 3.5.2 CircRNAs in osteoarthritis

**FIGURE 7:**
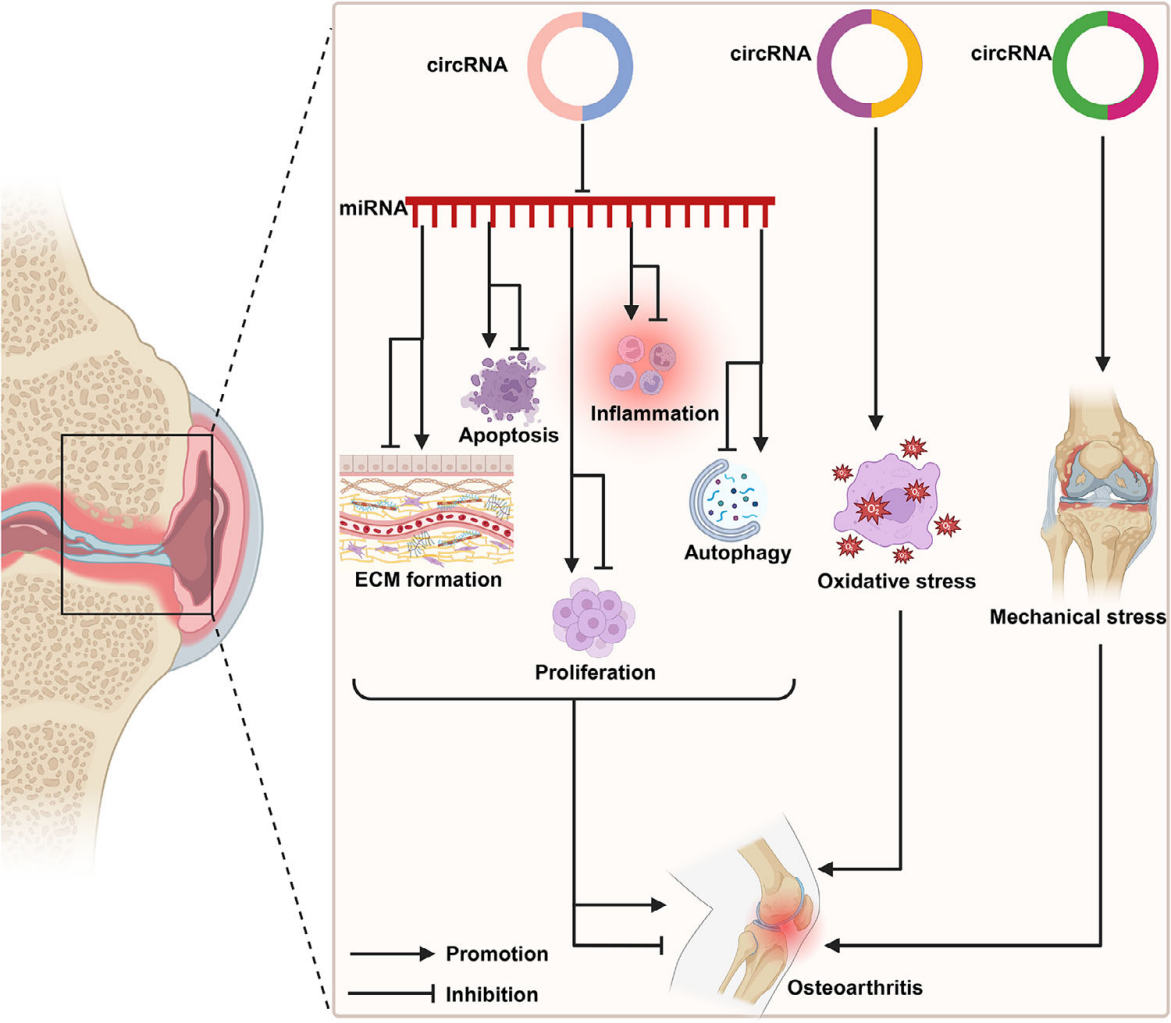
CircRNAs in osteoarthritis. This diagram elucidates the intricate interactions among circRNAs, miRNAs, and diverse cellular processes implicated in the pathogenesis of osteoarthritis. The circRNA–miRNA interplay is represented as a pivotal regulatory mechanism, modulating apoptosis, inflammation, autophagy, oxidative stress, mechanical stress, cellular proliferation, and extracellular matrix formation. Collectively, these processes play a crucial role in either facilitating or impeding the progression of osteoarthritis. The figure also highlights the importance of understanding these molecular interactions for potential therapeutic interventions in managing osteoarthritis.

It is also known as degenerative arthritis, senile arthritis, and hypertrophic arthritis.^484^ A key feature of osteoarthritis (OA) is abnormal chondrocytes, which cause joint pain, tenderness, stiffness, joint swelling, movement limitations, and joint deformity.^485^ A number of mechanisms are involved in OA, including inflammation, proliferation, apoptosis, autophagy, differentiation, oxidative stress, and mechanical stress (Figure 7). CircRNAs play a key role in OA pathogenesis by acting as intracellular miRNA sponges in chondrocytes, endplate chondrocytes, MSCs, synoviocytes, and macrophages.^486^ In addition, some circRNAs are involved in OA through other mechanisms, including RBP, m6A, or intercellular exosomal mechanisms, including ECM metabolic imbalance, local tissue inflammation, and multidirectional cell differentiation.^486^

Many circRNAs are disorderedly expressed in OA synovial tissues and immune cells, contributing to abnormal chondrocyte proliferation, synovial inflammation, cartilage erosion, bone damage, and abnormal immune responses. A study by Lin et al.^487^ found that circPhf21a was upregulated in an OA mouse model, affecting OA chondrocyte proliferation through its target VEGFA. CircumPhc3 was downregulated in OA and regulated FoxO1 through miR‐9‐3p, affecting chondrocyte metabolism.^488^ Further, circHGF competes with miR‐25‐3p to regulate its expression, affecting the proliferation and osteogenic differentiation of bone marrow mesenchymal stem cells (BMSCs).^489^ Further research indicates that miR‐9‐5p/KLF5 targets circStrn3 and regulates cartilage matrix metabolism and subchondral bone remodeling in OA.^490^ The findings suggest that circ_0008365 might be a potential therapeutic target or diagnostic biomarker for OA, as it acts as a sponge for miR‐338‐3p to regulate SOX9 expression in vitro.^491^ In another study, knockdown of hsa_circ_0134111 alleviated OA symptoms by sponging miRNA‐224‐5p.^492^ According to experiments, circVMA21 protected injured chondrocytes via miR‐495‐3p/FBWX7 axis through its role in OA rats.^493^ In OA, circCTNNA1 was downregulated, and its overexpression suppressed synoviocyte apoptosis by sponging miR‐29a.^494^ The overexpression of Hsa_circ_0005567 reduces chondrocyte apoptosis and may inhibit OA progression by promoting the polarization of M2 macrophages through the miR‐492/SOCS2 axis.^495^ CircRHOT1 augments the expression of CCND1 in chondrocytes by functioning as a sponge for miR‐142‐5p. Both the overexpression of CCND1 and the inhibition of miR‐142‐5p counteract the chondrocyte phenotypes resulting from circRHOT1 depletion, thereby playing a role in chondrocyte autophagy and proliferation during OA.^496^ A circRSU1–miR‐93‐5p–MAP3K8 axis modulates OA progression via oxidative stress regulation, serving as a possible therapeutic target.^497^

In a comparative analysis of synovial exosomes between OA synovial and control groups, 98 circRNAs were identified to be differentially expressed. Among these, 34 circRNAs were found to be enriched in the PI3K/Akt and autophagy pathways, exhibiting correlations with 7 mRNAs. These circRNAs play crucial roles in the pathological mechanisms underlying OA.^498^ CircRNAs act as miRNA sponges to inhibit mRNA translation responsible for OA development, with miRNA sponges acting as circRNA sponges.^499^ In exosomes originating from chondrocytes treated with IL‐1β, there was an elevation in CircRNA–BRWD1 levels. The miR‐1277/TRAF6 axis was modulated by exosomal circ‐BRWD1, and the suppression of circ‐BRWD1 hindered ECM degradation in chondrocytes stimulated by IL‐1β.^500^ Exosomes expressing CircRNA_0001236 might alleviate cartilage degradation, suppress OA progression, and enhance cartilage repair.^501^ Combined with sleep‐related circRNA3503, small EVs prevent OA. CircRNA3503‐OE‐sEVs, by sponging hsa‐miR‐181c‐3p and hsa‐let‐7b‐3p, effectively eased inflammation‐triggered apoptosis and restored the balance between ECM synthesis and degradation.^502^ Additionally, these sEVs facilitated chondrocyte renewal, thus helping to counteract the gradual depletion of chondrocytes.^502^

Cartilage degeneration is the main pathological characteristic of OA. Previous studies have shown that aberrant biological functions of chondrocytes, including ECM formation, apoptosis, inflammation, proliferation, and autophagy, are causally involved in OA pathogenesis. CircRNAs participate in OA pathogenesis in chondrocytes through ECM formation, apoptosis, proliferation, inflammation, and autophagy.^503^ CircSERPINE2 overexpression could alleviate HCs apoptosis and promote ECM anabolism through the miR‐1271‐ERG pathway, providing a potential therapeutic strategy for OA progression.^504^ CircPDE4D was significantly downregulated in OA cartilage tissues and during stimulation with inflammatory cytokines. Targeting the circPDE4D–miR‐103a‐3p–FGF18 axis might provide a promising approach for OA therapy.^505^ Another study revealed an important role of the CircCDK14/miR‐125a‐5p/Smad2 axis in OA progression, providing a potential molecular therapeutic strategy.^506^ CircCDH13 contributes to OA pathogenesis by functioning as a sponge for miR‐296‐3p and regulating the miR‐296‐3p–PTEN pathway. Silencing CircCDH13 could reduce chondrocyte apoptosis, inhibit ECM catabolism, and promote ECM anabolism.^507^ Certain circRNAs or related pathways can be used as diagnostic and prognostic biomarkers for OA treatment, providing potential targets for developing effective interventions.

#### 3.5.3 CircRNAs in Sjögren's syndrome

Sjögren's syndrome (SS) is a chronic autoimmune disorder of the exocrine glands characterized by lymphocytic infiltration in the salivary and lacrimal glands (LGs), resulting in oral and eye dryness.^508^ Extraglandular manifestations such as musculoskeletal pain, fatigue, and systemic features also develop in a significant percentage of patients. This exocrinopathy can occur alone (primary SS, pSS) or secondary to another autoimmune disease such as RA, SLE, and systemic sclerosis (SSc).^509^ Studies have demonstrated significantly different circRNA expression profiles in the minor salivary glands of pSS patients compared with non‐pSS individuals by RNA‐seq. Researchers identified 13 significantly DEcircRNAs, most of which showed upregulated expression in pSS. circRNA sequencing indicated circ‐IQGAP2 (hsa_circ_0129656) and circ‐ZC3H6 (hsa_circ_0001062) as noninvasive biomarkers of pSS.^510^

#### 3.5.4 CircRNAs in spondyloarthritis

Ankylosing spondylitis (AS) is a typical inflammatory rheumatic disease characterized by pathological new bone formation, ankylosis, and chronic inflammation in the spine, with osteoclastogenesis playing a critical role in bone formation.^511^ Previous research has shown that exosomes from MSCs participate in the modulation of osteoclastogenesis in vitro.^512^ Researchers discovered that the circRNA derived from TNFRSF1B, circ‐0110634, was significantly upregulated in AS‐MSCs and exosomes from AS‐MSCs compared with HD‐MSCs and HD‐MSC exosomes. They found that circ‐0110634 plays an inhibitory role in osteoclastogenesis, with AS‐MSCs delivering exosomal circ‐0110634 to restrain the osteoclastogenesis of PBMCs. This role is similar to that of hsa_circ_0002715 and hsa_circ_0035197 in patients with new‐onset RA.^513^ Researchers investigated the circRNA profiles of exosomes via high‐throughput RNA sequencing and identified 56 DEcircRNAs in the exosomes of patients with AS compared with healthy controls. These circRNAs were mainly involved in the negative regulation of the TF nuclear factor‐κB and bone remodeling, which are potentially related to AS. The most highly AS‐correlated pathways identified were the “Notch signaling pathway” and those primarily involved with “cholinergic synapse.”^514^ Another study conducted circRNA and miRNA expression profiling of osteogenically differentiated BMSCs from patients with AS compared with healthy donors using RNA sequencing. The results showed that 418 circRNAs were significantly differentially expressed, with 204 circRNAs upregulated and 214 downregulated. In vitro experiments showed that silencing hsa_circ_0070562 weakened the osteogenesis of AS‐BMSCs, indicating that circ_0070562 functions as a pro‐osteogenic factor and might serve as a potential biomarker and therapeutic target for AS.^515^

#### 3.5.5 CircRNAs in osteoporosis

Osteoporosis is one of the most common systemic metabolic bone diseases, particularly prevalent in postmenopausal women.^516^ It occurs due to excessive bone resorption and impaired bone formation.^517^ Accumulating evidence suggests that dysregulated circRNAs are associated with the occurrence and progression of osteoporosis, regulating osteoblast and osteoclast differentiation, and may serve as potential biomarkers for the disease. For example, studies have identified circARSB, circAKT3, circPTEN, and circTRPM7 as being associated with osteogenic differentiation during glucocorticoid‐induced osteoporosis (GIOP) through a circRNA‐targeted miRNA–mRNA axis, providing insights into the pathophysiological mechanisms of GIOP.^518^ A total of 387 circRNAs were found to be differentially expressed in osteoporosis compared with healthy controls, with circDNAH14 (hsa_circ_0016624) preventing osteoporosis by regulating BMP2 via miR‐98 sponging.^519^ Among women with postmenopausal osteoporosis (PMO), Liu et al. identified 250 DEcircRNAs, with 64 showing a decreasing expression trend and 186 showing increased expression. Circ_0007059 might play a crucial role in osteoclastogenesis via the miR‐378/BMP‐2 signaling pathway, suggesting that targeting the circ_0007059/miR‐378/BMP2 axis could be a novel therapeutic approach for osteoporosis.^520^ Qiao et al. found that circRNA_0048211 negatively targets miRNA‐93‐5p to upregulate BMP2, thus alleviating the progression of PMO.^521^ Furthermore, using postmenopausal mouse models, Wang et al.^522^ focused on DEcircRNAs in osteoporotic mice. They identified two ceRNA regulatory pathways in this osteoporosis mouse model: novel circRNA 0020/miR‐206‐3p/Nnmt and circRNA 3832/miR‐3473e/Runx3, validated by real‐time PCR.^522^

Some studies have revealed that hsa_circ_0001275, hsa_circ_0002060, and hsa_circ_0006859 are recognized as potential novel diagnostic biomarkers of osteoporosis.^523^, ^524^, ^525^ CircFOXP1 can promote osteogenic differentiation of ADSCs and bone regeneration via miR‐33a‐5p.^526^ Circ_0007059 can inhibit bone marrow stromal cell differentiation into osteoclasts via the mRNA‐378/BMP‐2 axis.^520^ By constructing a circRNA–miRNA–TF–mRNA regulatory network using bioinformatics analysis, researchers identified four associated circRNAs in the ceRNA network: hsa_circ_0023417, hsa_circ_0078309, hsa_circ_0063533, and hsa_circ_0036760, suggesting that these circRNAs may be important regulators of osteoporosis development.^516^

## 4 CircRNAs IN CANCER

In this section, we examine the pivotal role of circRNAs in cancer biology, focusing on their implications in tumorigenesis, tumor progression, and therapeutic resistance. By exploring their interactions within the tumor microenvironment (TME) and their influence on cancer cell behavior, we highlight circRNAs as potential biomarkers for early diagnosis and as targets for innovative therapeutic strategies. This section aims to elucidate the complex landscape of circRNAs in cancer and their promise for advancing cancer diagnostics and treatments.

### 4.1 Multiple cancer–circRNA signaling pathways

Carcinogenesis is closely linked to several signaling pathways. The complexity of signals and their functional roles are essential for the emergence and spread of malignancies and other diseases. Various types of human malignancies have been linked to dysregulated signaling pathways.^527^, ^528^, ^529^, ^530^ Additionally, certain signaling pathways trigger epithelial–mesenchymal transition (EMT), a significant accelerator of cancer progression. During this process, considered an inducer of cancer metastasis, epithelial cells lose their polarity and differentiated state, obtaining a mesenchymal‐like phenotype. Although the underlying processes remain unclear, it has been discovered that certain circRNAs function as ceRNAs for miRNAs involved in EMT signaling pathways, contributing to tumor growth.^531^ In this study, we explore the roles of signaling pathways in malignant carcinomas, focusing on molecular mechanisms and potential avenues for future intervention.

#### 4.1.1 The PI3K/AKT/mTOR signaling pathway and circRNAs

Numerous malignancies exhibit highly activated PI3K/AKT/mTOR signaling pathways, facilitated by upstream oncoprotein receptor tyrosine kinases, G‐protein‐coupled receptors, or RAS oncogenes. Carcinogenesis is associated with dysregulation of the PI3K/AKT/mTOR signaling pathway. Consequently, research has increasingly focused on the associations of circRNAs with the PI3K/AKT signaling pathway.^532^, ^533^ CircRNAs commonly function as ceRNAs for miRNAs in tumor progression. Based on the ceRNA mechanism, downstream pathways are activated or suppressed by sponging miRNAs. For instance, in CRC, circCDYL (116), circ_0001313,^533^ hsa_circRNA_002144,^534^ and circIL4R^535^ utilize the ceRNA mechanism to regulate the PI3K/AKT signaling pathway, either promoting or inhibiting tumor growth. By acting as a miR‐149‐5p sponge, circNRIP1 enhances the progression of gastric cancer (GC) via the AKT1/mTOR pathway.^536^ CircFGFR3 regulates the ERK1/2 and AKT signaling pathways in non‐small cell lung cancer (NSCLC) by sponging miR‐22‐3p.^537^ Patients with hepatitis B‐associated HCC have a poor prognosis due to elevated levels of circRNA‐100338, which are linked to the activation of the mTOR signaling pathway in HCC via the circRNA‐100338/miR‐141‐3p/RHEB axis.^538^ According to Shi et al.^539^ circ 0014359 sponges miR‐153, which regulates the expression of p‐AKTser473 and accelerates the progression of gliomas. CircPLEKHM3^540^ and circKDM4B^541^ are other miR‐sponging circRNAs that, in ovarian cancer (OC) and breast cancer respectively, promote or suppress the AKT signaling pathway.

Furthermore, it has been discovered that circRNAs regulate tumor progression by altering epigenetic processes. Circ‐0124554 (circ‐LNLM), for instance, inhibits AKT ubiquitination and thus enhances CRC hepatic metastasis.^542^ Additionally, in oral squamous cell carcinoma (OSCC) and thyroid carcinoma respectively, circ_0067934 and circ_0007059 influence malignant cell behavior through phosphorylating AKT/mTOR.^543^, ^544^ According to a recent report, circ‐ANAPC7 regulates the CREB–miR‐373–PHLPP2 feed‐forward loop through the PHLPP2–AKT–TGF‐β signaling axis, preventing pancreatic cancer muscle atrophy and tumor progression.^545^ Interestingly, the PI3K/AKT/mTOR signaling pathway has been shown to influence tumor progression through circRNAs that encode novel proteins. The circ‐AKT3 RNA‐encoded tumor suppressor protein, for instance, competes with active PDPK1, thus reducing the tumorigenicity of glioblastoma.^50^ This evidence demonstrates that circRNAs play a dual role in protein modification and oncogenic promoter activity.

#### 4.1.2 The Wnt signaling pathway and circRNAs

Positive associations exist between tumorigenesis and the canonical Wnt signaling pathway. In summary, a destruction complex composed of casein kinase 1, adenomatous polyposis coli (APC), glycogen synthase kinase‐3 (GSK‐3), β‐catenin, and axin first phosphorylates and then ubiquitinates β‐catenin inside the cytoplasm. The formation of this complex is inhibited by the presence of Wnt ligands. Consequently, β‐catenin, the primary protein, is transported from the extracellular space to the nucleus to activate downstream proteins targeted by Wnt.^546^, ^547^ The sponging of miRNAs activates or represses downstream pathways based on the ceRNA mechanism.^548^, ^549^ CircRNAs within the cytoplasm interact with proteins in the destruction complex, influencing Wnt activation. The Wnt/β‐catenin signaling pathway is negatively regulated by APC, a key protein in the destruction complex. The tumor‐suppressive properties of has_circ_0009361 were revealed by Geng et al.,^546^ along with its ability to sponge miR‐582 and subsequently enhance the expression of APC2, which then influences the Wnt/β‐catenin pathway in CRC. The Wnt signaling pathway is activated or suppressed by Wnt ligands binding to the Wnt antagonist Dickkopf‐1 (Dkk1), lipoprotein‐related protein (LRP), or Frizzled (FZD) receptors on the cell surface.^550^ Because DKK1 selectively binds to LRP5/6, it inhibits the formation of the Wnt–LRP5/6–FZD complex and suppresses the downstream Wnt pathway. Hsa_circ_0000523, hsa_circ_0000177, and circ_100290 regulate these surface proteins in gliomas and CRCs and are associated with the Wnt pathway.^551^, ^552^, ^553^

Several circRNAs interact with the downstream genes of the Wnt pathway when β‐catenin enters the nucleus. CircMTCL1 accelerates the development of advanced laryngeal squamous cell carcinoma by inhibiting the degradation of C1QBP ubiquitin and regulating the accumulation of β‐catenin in the nucleus and cytoplasm.^554^ The Wnt signaling pathway is stimulated in endometrial cancer through the hsa_circ_0002577/miR‐197/CTNND1 axis, which also affects the expression of c‐Myc, cyclin D1, and β‐catenin.^555^ In papillary thyroid carcinoma (PTC), Bi et al.^556^  showed that circRNA 102171 interacts with CTNNBIP1 and then inhibits its interaction with the β‐catenin/TCF3/TCF4/LEF1 complex, thus stimulating the Wnt/β‐catenin pathway. The Wnt pathway is influenced by several novel proteins encoded by circRNAs.^547^, ^557^, ^558^, ^559^ The competitive interaction between the AXIN1‐295 aa protein, encoded by circAXIN1, and APC has been shown to contribute to the malfunction of the ‘destruction complex’ in GC, highlighting a key aspect of circRNA involvement in cancer pathology.^557^ The novel protein circβ‐catenin‐370 aa, encoded by circβ‐catenin in HCC, stabilizes β‐catenin and activates the Wnt pathway.^547^ Additionally, hsa_circ_0007059 appears to inhibit the interaction between β‐catenin and Wnt3a, potentially suppressing EMT in lung cancer.^560^ Furthermore, it has been demonstrated that circRNAs function as feedback loops to regulate cancer growth. CircESRP establishes a positive feedback loop that influences cancer progression through EMT.^561^ Accordingly, research has demonstrated the crucial role of circRNAs in the circRNA/Wnt/β‐catenin signaling pathway. If these findings are validated, they could lead to the development of innovative cancer treatments.

#### 4.1.3 The notch and hippo signaling pathways and circRNAs

Neurogenesis, angiogenesis, as well as overall cell survival and growth, are regulated by the Notch signaling pathway. Transmembrane proteins known as Notch receptors (NOTCH1, NOTCH2, NOTCH3, and NOTCH4) bind to specific ligands to trigger a myriad of biochemical reactions.^562^ CircRNAs commonly function as ceRNAs in Notch signaling, sponging up miRNAs to activate pathways by directly stimulating receptors and/or their ligands.^563^, ^564^, ^565^, ^566^ Aberrant Notch signaling is often linked to genetic alterations in key components, particularly NOTCH1. According to Xu et al.,^563^ circNFIX binds to NOTCH1 and sponges miR‐34a‐5p, activating the Notch pathway in gliomas. By sponging miR‐34a and consequently regulating Notch1, circ‐ASH2L promotes tumor growth in pancreatic ductal adenocarcinoma (PDAC).^565^ These findings suggest that circRNAs involved in Notch signaling could be utilized to develop novel approaches to inhibit the progression of cancer.

Numerous tumor suppressors and oncogenes are part of the Hippo signaling pathway. The PDZ‐binding motif and YAP become active and accumulate in the nucleus following the inactivation of the Hippo signaling pathway, subsequently enhancing cellular proliferation.^39^, ^567^, ^568^ Multiple studies have demonstrated the involvement of circRNAs in the Hippo signaling pathway, with dominant mechanisms including ceRNA competition,^569^, ^570^, ^571^ translation of novel proteins,^39^ feedback loops,^572^ and YAP nuclear accumulation.^573^ Circ‐LECRC, generated from YAP, acts as a “brake signal,” inhibiting the overactivation of carcinogenic YAP signaling in CRC.^572^ Louis and Coulouarn^528^ discovered that circACTN4 elevates YAP1 levels by acting as a miR‐424‐5p sponge, thereby recruiting Y‐box binding protein 1 (YBX1) to trigger FZD7 transcription and accelerate the progression of intrahepatic cholangiocarcinoma. According to Wu et al.,^567^ circYap binds to Yap mRNA, PABP, and eIF4G to prevent Yap translation initiation in breast cancer cells. The novel peptide circPPP1R12A‐73 aa, encoded by circPPP1R12A, accelerates the development of CRC by activating the Hippo‐YAP signaling pathway.^39^ Liu et al. ^569^ demonstrated that YAP1 suppresses the transcription of circRNA‐000425 in GC, thereby enhancing the oncogenic activity of miR‐106 and miR‐17. Altogether, these findings suggest that circRNAs influence tumor‐related signaling pathways by serving as miRNA sponges or interacting with proteins, thereby affecting the functioning of the Hippo/YAP pathway.

#### 4.1.4 The p53/Bcl‐2 signaling pathway and circRNAs

The apoptotic process and the cell cycle are regulated by the tumor protein 53 (p53) via a myriad of distinct routes. Additionally, through the ceRNA mechanism, circRNAs such as circ‐BRIC6, circ‐0001785, hsa_circ_006100, hsa_circ_0002874, circPVT1, and circ_0021977 regulate the p53 signaling pathway.^574^, ^575^, ^576^, ^577^, ^578^, ^579^ The homo‐tetrameric TF known as wild‐type p53 protein (wt‐p53) acts as a tumor suppressor by controlling the transcription of downstream target genes.^580^, ^581^ Mutant p53 (mut‐p53) loses its tumor‐suppressive functions and gains tumor‐promoting activities, known as GOF activities. Elevated levels of mut‐p53 proteins are necessary for effective mut‐p53 GOF activity in cancer cells. When the ability of remaining wt‐p53 to regulate tumor growth is inhibited by the creation of a hetero‐tetrameric mut‐p53/wt‐p53 complex, tumor cells proliferate, survive, migrate, and invade. It has been discovered that mut‐p53 and circRNA interact.^582^ For instance, mut‐p53 activates circPVT1^576^ and circ‐Ccnb1.^583^ CircPVT1 and mut‐p53 expression profiles were found to be upregulated in cancerous tissues compared with normal tissues in a study of 115 patients with head and neck squamous cell carcinoma. The mut‐p53/YAP/TEAD complex is hypothesized to be a mechanism for enhancing circPVT1 transcription.^576^

Wt‐p53 promotes circ‐Ccnb1 expression in breast cancer cells, but wt‐p53 suppression or mut‐p53 expression inhibits circ‐Ccnb1 expression. Circ‐Ccnb1 separates the CyclinB1/Cdk1 mitotic complex, which inhibits carcinogenesis and cellular invasion.^583^ Mechanistically, it has been proposed that circRNAs and p53 directly bind to prevent the degradation of p53. This interaction could prevent p53 from being ubiquitinated by E3 ubiquitin ligases (such as MDM2) and subsequently degraded.^582^ The p53/MDM2 complex is disrupted when the circRNA CDR1as binds to p53, leading to the stabilization of p53 in glioblastoma.^580^ Conversely, when circ‐DNMT1 binds to p53, it induces nuclear translocation of p53 and AUF1, thereby mediating DNMT1 upregulation and inhibiting p53 expression in breast cancer.^584^ Additionally, circRNAs influence Bcl‐2, a downstream target of p53, leading to apoptosis in HCC, CRC, and GC cells.^574^, ^578^, ^585^, ^586^ Furthermore, in OSCC and osteosarcoma, circ_0007534, circ_0001785.^579^, ^587^ Moreover, a new regulatory mechanism related to the p53 signaling pathway involves the generation of feedback loops. The FMR1/circCHAF1A/miR‐211‐5p/HOXC8 feedback loop in glioblastoma regulates the proliferative rate and carcinogenesis through MDM2‐dependent p53 signaling.^588^ The most prevalent cause of cancer is p53 inactivation or mutations, yet p53 stability is regulated by the formation of the p53/MDM2 complex. Consequently, p53 has emerged as one of the most promising treatment targets for cancer.

#### 4.1.5 CircRNAs promote EMT through the TGF‐β/Smad signaling pathway

Receptor‐regulated Smad (R‐Smad), which triggers transcriptional responses by attaching to Smad binding sites in cell nuclei and suppressing the expression of epithelial genes, mediates TGF‐β1 canonical signaling.^589^ EMT, initiated and perpetuated by TGF‐β1, is a crucial phase in cancer metastasis. CircRNAs also play a role in the process by which the TGF‐β/Smad pathway regulates the development and metastasis of various malignancies. CircPTK2 was shown by Wang et al.^590^ to be significantly downregulated in NSCLC cells and to be inversely related to EMT induced by TGF‐β. CircANKS1B binds to miR‐152‐3p and miR‐148a‐3p in triple‐negative breast cancer (TNBC), thereby enhancing the expression of upstream TF 1 (USF1), which in turn activates TGF‐β1.^591^ Through the ceRNA mechanism, circPTK2 and circANKS1B can either stimulate or inhibit EMT in tumorigenesis. The ceRNA mechanism also applies to the functions of circRIP2^592^ in bladder cancer and circANKS1B^593^ in OSCC. When CircPTEN1 binds to the MH2 motif of Smad4, it disrupts its interaction with Smad2/3, thereby inhibiting the expression of downstream genes involved in EMT induced by TGF‐β.^594^ Additionally, circRNAs such as circ‐DOCK5^595^ and circUHRF1^596^ have been demonstrated to form feedback loops that modulate the TGF‐β/Smad signaling pathway.

Here, we have concentrated on the canonical pathway linked to carcinogenesis and the development of cancer. CircRNAs also interact with other signaling pathways, including the carcinogenic MAPK and JAK/STAT pathways.^597^ Any abnormal interaction between these pathways may lead to cancer progression. Collectively, these data suggest that circRNAs play critical roles in regulating the behavior of cancer cells.

### 4.2 CircRNAs and exosomes

#### 4.2.1 Exosomal biogenesis and activity

The membrane‐bound EVs known as exosomes are generated from the late endosomal limiting membrane. These single‐membrane, small, secreted organelles range from 40 to 100 nm in diameter.^598^, ^599^ Moreover, they significantly affect various biological activities, including physiological and pathological inter‐ and intra‐cellular communication, as well as the transfer of biomolecules such as enzymes, proteins, RNAs, and lipids in diverse disorders.^600^, ^601^ The endosomal system accounts for the initial step in exosomal biogenesis. Following various maturation processes, invagination of the endosomal membrane leads to the formation of multivesicular bodies (MVBs). In addition to producing intraluminal vesicles, MVBs are also degraded by fusing with lysosomes.^602^ Exosomal biogenesis and production involve multiple mechanisms. The ESCRT‐mediated biogenesis of MVBs represents a frequently investigated pathway, dependent on intracellular homeostasis and cell type.^603^ Exosomes have emerged as potential diagnostic and prognostic biological markers, as well as treatment targets, significantly impacting the TME, tumor migration, invasion, progression, chemoresistance, and targeted drug delivery.^604^, ^605^, ^606^, ^607^

#### 4.2.2 The properties and biological functions of exosome

Exosomes serve as critical mediators for intercellular communication, particularly in the TME.^608^ An increasing number of studies indicate the presence of abundant binding sites for miRNAs in circRNAs. CircRNAs can stabilize miRNAs, regulate alternative splicing, encode small peptides, and serve as protein “decoys” and miRNA inhibitors (“sponges”) to exert their effects.^609^, ^610^ ExocircRNAs derived from tumor cells can influence target cells or organs via exosome transportation, potentially promoting or suppressing tumor growth.^611^

As an increasing number of studies are conducted, researchers are analyzing the relationship between exosomes and circRNAs in tumors, reporting that exocircRNAs are novel biological markers for tumor detection, providing a new direction for tumor diagnosis.^62^, ^612^ According to Dou et al.,^28^ circRNAs were discovered in tumor‐derived exosomes, and their abundances were higher compared with those in mutant KRAS cells, suggesting a potential link between circRNAs and tumorigenesis. This indicates the potential role of exocircRNAs as tumor biomarkers.

According to Shang et al.,^613^ circPACRGL was remarkably upregulated in CRC cells when tumor‐derived exosomes were added, indicating its role in inhibiting CRC proliferation and metastasis. The level of exosomal circSHKBP1 was significantly increased in GC serum and tissues, and it was associated with unfavorable prognoses and advanced tumor node metastasis (TNM) stages. Additionally, exosomal circSHKBP1 enhanced GC cell proliferation, motility, invasiveness, and angiogenesis by modulating the miR‐582‐3p/HUR/VEGF axis and inhibiting the degradation of HSP90.^614^

Hsa_circ_0074854 may be transferred by exosomes from NSCLC cells to macrophages, where its expression level was elevated. To inhibit NSCLC invasion and migration, the knockdown of Hsa_circ_0074854 suppresses HuR interaction and macrophage M2 polarization.^614^ ExocircRNAs derived from MSCs represent a potential antitumor treatment. By functioning as a sponge for miR‐216a‐3p and thus releasing HDAC4, exosomal circFBXW7 generated from MSCs inhibits synovial cell proliferation, migration, and invasion, and suppresses the inflammatory response in RA.^483^

Recent studies have revealed that circRNAs are highly abundant in human platelets compared with other hematopoietic cells and are packaged and released in platelet‐derived exosomes and microvesicles.^615^ Given that platelets are implicated in tumor migration, inflammation, and hemostasis, research on exocircRNAs may provide a new avenue for disease diagnosis and treatment.^615^

#### 4.2.3 Exosomes contain abundant and stable CircRNA

In an RNA‐Seq study of total RNA with depleted ribosomal RNA from MHCC‐LM3 liver cancer cells and the exosomes they produce, it was first discovered that exocircRNAs are abundant and stable.^26^ RNA‐Seq analysis revealed that the ratio of circRNA levels to their linear RNA counterparts in exosomes was approximately sixfold higher than in the producer cells. Additionally, circRNA levels remained stable even after being incubated for up to 24 h at ambient temperature.^26^ According to these findings, exosomes contain an exceptionally high concentration of stable circRNAs.

ExocircRNA exhibits a distinct expression pattern from that of the parent cells,^26^, ^28^ indicating that exosomes actively incorporate circRNAs. Exosome RNA sorting has been shown to involve a variety of methods, including specialized RNA sequences and/or secondary structures associated with RBPs.^616^, ^617^ The precise mechanism by which circRNAs are sorted into exosomes remains undetermined.

CircRNA may be more abundant in exosomes due to its stability. The extended half‐life of circRNA, relative to linear RNA, is attributed to its resistance to exonuclease digestion, as it lacks a 5′ or 3′ end.^73^, ^105^, ^618^ Although the production of nascent circRNA is not highly efficient due to its lengthy half‐life, it tends to accumulate in cells with slower division rates, such as neuronal cells.^119^, ^619^, ^620^ Additionally, exosomes can protect their RNA cargo from degradation by RNases.^621^, ^622^ Consequently, circRNA that has been incorporated into exosomes may be highly stable.

The concentration of miRNA in producer cells can influence the amount of circRNA present in exosomes. Among the most extensively studied circRNAs, CDR1‐AS has been shown to bind to miR‐7 and significantly inhibit miR‐7 activity.^33^ When miR‐7 is produced ectopically, CDR1‐AS is significantly reduced in exosomes but slightly increased in cells.^26^ This evidence suggests that the relative miRNA levels in producer cells are at least partially responsible for regulating the trafficking of circRNAs into exosomes.

#### 4.2.4 ExocircRNAs’ biological functions

CircRNAs may influence the expression of miRNA target genes in recipient cells and maintain the miRNA sponging activities of exosomes. The exosomal form of CDR1‐AS is the first circRNA shown to maintain biological activity by sponging miR‐7, exosomal CDR1‐AS can potentially reverse miR‐7‐mediated growth inhibition in recipient cells.^26^ There is increasing evidence that cancer cells, endothelial cells, and adipose cells interact with one another to promote cellular proliferation, invasion, EMT, metastasis, chemoresistance, and cancer cachexia.

##### Cell proliferation

Several reports indicate that exocircRNAs enhance cellular proliferation. Human hepatic epithelial cells exposed to arsenite release circRNA 100284, which is then conveyed via exosomes to surrounding normal cells. By acting as a miR‐271 sponge and upregulating EZH2 expression, exocircRNA_100284 accelerates the cell cycle and enhances cellular proliferation.^623^ A circRNA called exosomal CDR1‐AS promotes the onset and progression of HCC. By acting as a sponge for miR‐1270 and increasing alpha‐fetoprotein expression in HCC cells, the upregulation of CDR1‐AS enhances cell proliferation and motility. Exosomes carrying CDR1‐AS are then delivered to nearby cells, stimulating their migration and proliferation.^624^ Circ0000284 is found in plasma exosomes, malignant tissues, and cholangiocarcinoma cell lines. Circ0000284 upregulates LY6E expression and encourages cholangiocarcinoma cell proliferation, migration, and invasion by acting as a sponge for miR‐637 in cells. Circ0000284, released into exosomes, is then delivered to surrounding cells, promoting their proliferation and motility.^625^

ExocircRNAs produced by healthy cells inhibit proliferation in recipient cells, in contrast to those produced by tumors, which increase it. For example, circ0051443, downregulated in HCC patients’ plasma exosomes, is released by healthy hepatic cells and transported to adjacent HCC cells, where it increases BAK1 expression and sponges miR331‐3p, resulting in cell cycle arrest and apoptosis in tumor cells.^626^

##### EMT, invasion, and metastasis

Intercellular communication mediated by exosomes has been shown to facilitate the formation of prometastatic niches, induce EMT, and alter the TME, all of which contribute to tumor invasion and metastasis.^627^, ^628^, ^629^ Recent research implicates circRNAs in exosomes in the invasion and metastasis of tumors. Pancreatic cancer cells release circPDE8A, and overexpression of exosomal circPDE8A in patient plasma is linked to a poorer prognosis. In pancreatic cancer cells, circPDE8A acts as a miR‐338 sponge, activating the metastasis‐associated in colon cancer (MACC)/MET/ERK or AKT pathways and promoting invasive development. Additionally, by stimulating the MACC/MET/ERK or AKT pathway, exosomal circPDE8A promotes EMT in recipient cells.^630^ CircRanGAP1 is upregulated in plasma exosomes from preoperative GC patients compared with those from healthy controls and postoperative GC patients. More significantly, the plasma exosomes from these individuals increased the migratory and invasive rate of GC cells, suggesting that exosomal circRanGAP1 may perform a role in the onset and advancement of GC.^631^ As opposed to nonmetastatic or low‐metastatic HCC cells, exosomes from highly metastatic HCC cells have a higher concentration of circPTGR1. The miR‐449a/MET pathway, activated by coculture with exosomal circPTGR1, promotes HCC cell invasion and motility. Additionally, patients with HCC have a poorer prognosis and more advanced clinical stages when circPTGR1 expression is high in serum exosomes.^632^

Exosoma CircRNA may facilitate the invasion of cancerous cells not only by increasing their invasiveness but also by changing the TME. HUVECs receive circIARS via exosomes secreted from pancreatic cancer cells. Pancreatic cancer cells may more readily invade HUVECs due to the presence of exosomal circIARS, which increases their permeability by upregulating RhoA and sponging miR‐122.^633^ ExocircRNA‐100338, which is increased in an HCC cell line with a high level of metastasis, also enhances the ability of HUVECs to permeate, proliferate, and undergo angiogenesis. CircRNA‐100338 may bind RBPs such as NOVA2, which is known to regulate vascular growth and lumen development, as shown by RNA pull‐down analysis. These findings suggest that circRNA‐100338 could regulate angiogenesis by interacting with NOVA2, but further research is needed to confirm these hypotheses.^63^

Multiple reports indicate that various circRNAs are implicated in metastasis. CircPRMT5, commonly upregulated in urothelial carcinoma of the bladder (UCB) cells, acts as a miR‐30c sponge and modulates the SNAIL1/E‐cadherin pathway, stimulating EMT in UCB cells. Additionally, the presence of circPRMT5 in serum and urine exosomes has been positively associated with lymph node metastasis (LNM) and advanced tumor progression.^548^ OC tissues exhibit elevated levels of circWHSC1, which upregulates hTERT and MUC1, acts as a miR‐1182 and miR‐145 sponge, and enhances cell proliferation, motility, and invasiveness. Exosomal circWHSC1 upregulates N‐cadherin and MUC1, causing peritoneal mesothelial cells to adopt a fibroblast‐like morphology. Additionally, OC cells, when injected intraperitoneally into mice along with exosomes from cells overexpressing circWHSC1, enhance peritoneal dissemination. This suggests that exosomal circWHSC1 may be transported to peritoneal mesothelial cells, facilitating peritoneal dissemination in vivo.^634^

##### Chemoresistance

CircRNAs in exosomes are associated with chemoresistance. In a microarray analysis of exosomal RNAs from chemosensitive and chemoresistant CRC cells, the expression levels of 105 circRNAs were significantly upregulated, while those of 34 circRNAs were downregulated in exosomes from chemoresistant CRC cells. Research suggests that hsa_circ_0032883 could be a potential biomarker for chemotherapy response, as it was found to be significantly elevated in the serum exosomes of chemosensitive patients compared with those who are chemoresistant.^635^ Another study identified a positive correlation between ciRS‐122 and chemoresistance in CRC. ciRS‐122 enhances the expression of PKM2 and acts as a miR‐122 sponge. ciRS‐122, transferred from chemoresistant cells to recipient cells, promotes glycolysis, and reduces drug sensitivity.^636^

##### Adipocyte browning

CircRNAs may be transported into adipose tissues by exosomes, potentially causing cancer cachexia in addition to facilitating tumor cells' intracellular crosstalk. Unlike cancer without cachexia, which typically has a better prognosis, cancer cachexia is characterized by an uncontrollable decrease in total body weight. Reports indicate that cancer cachexia is driven by abnormally increased thermogenesis due to adipocyte browning.^637^, ^638^ These findings suggest that exocircRNAs from tumors may contribute to the development of cancer cachexia.

##### ExocircRNA's potential in clinical settings

CircRNAs can be found in serum,^26^ urine,^66^ and saliva,^108^ with their tertiary structure contributing to their stability. Additionally, various circRNAs are dysregulated in different malignancies.^639^ To date, circRNAs have shown promise as noninvasive biomarkers for tumor detection. Recent evidence using high‐throughput sequencing indicates that various exocircRNAs have potential as diagnostic biomarkers for cancers. Circ‐0051443 was shown to be considerably reduced in exosomes generated from the plasma of patients with HCC compared with healthy controls utilizing microarray analysis. The diagnostic potential of exosomal circ‐0051443 was evaluated based on plasma exosomes from 60 HCC patients and 60 healthy individuals. An AUC value of 0.8089 for exosomal circ‐0051443 indicated moderate diagnostic accuracy.^626^ Hsa_circ_0000419, circ‐KIAA, and hsa_circ_0065149 exhibited lower levels in plasma exosomes from patients with GC than in those from healthy individuals. AUC values of 0.84, 0.75, and 0.64, respectively, for these circRNAs indicated reasonable diagnostic accuracy.^78^, ^640^, ^641^ In a circRNA microarray analysis of three pairs of esophageal squamous cell carcinoma (ESCC) and nontumor tissues, 1045 circRNAs were found to be upregulated and 1032 downregulated in ESCC. Both hsa_circ_0043603 and hsa_circ_0001946 were found to be significantly downregulated in plasma from 50 ESCC patients compared with 50 controls. When used together as a diagnostic tool, these two circRNAs achieved a specificity of 98% and a sensitivity of 84%.^642^ Exosomal hsa‐circ‐0004771 levels in the circulation were shown to be considerably elevated in the serum samples of 110 CRC patients in contrast to 35 patients with benign intestinal diseases and 35 healthy controls. Exosomal hsa‐circ‐0004771 in the circulation demonstrated a moderate ability to distinguish between stage I/II CRC patients and healthy controls, with AUC values of 0.86 and 0.88, respectively.^643^ Poor prognosis and clinical response to treatment in small‐cell lung cancer were linked to serum exosomal FECR1.^644^ CircPDAC was elevated and detectable in the sera of pancreatic cancer patients (specificity 0.90, sensitivity 0.45). Additionally, circPDAC was also detected in the sera of patients with intraductal papillary mucinous neoplasm, a known precancerous lesion.^645^

### 4.3 ExocircRNAs and cancer

#### 4.3.1 ExocircRNAs and NSCLC

ExocircRNAs play crucial roles in the regulation of NSCLC development and progression by targeting cancer‐related signaling pathways and/or regulating the expression of genes involved in NSCLC pathology, including cell proliferation, apoptosis, metastasis, stemness, and therapy resistance.^646^ Additionally, certain exocircRNAs are differentially expressed between NSCLC tissues and corresponding normal tissues, highlighting their potential as promising biomarkers for early diagnosis and prognosis of NSCLC. Here, we provide a summary of dysregulated exocircRNAs associated with NSCLC.

##### Biological properties and activity of exocircRNAs in NSCLC

**FIGURE 8:**
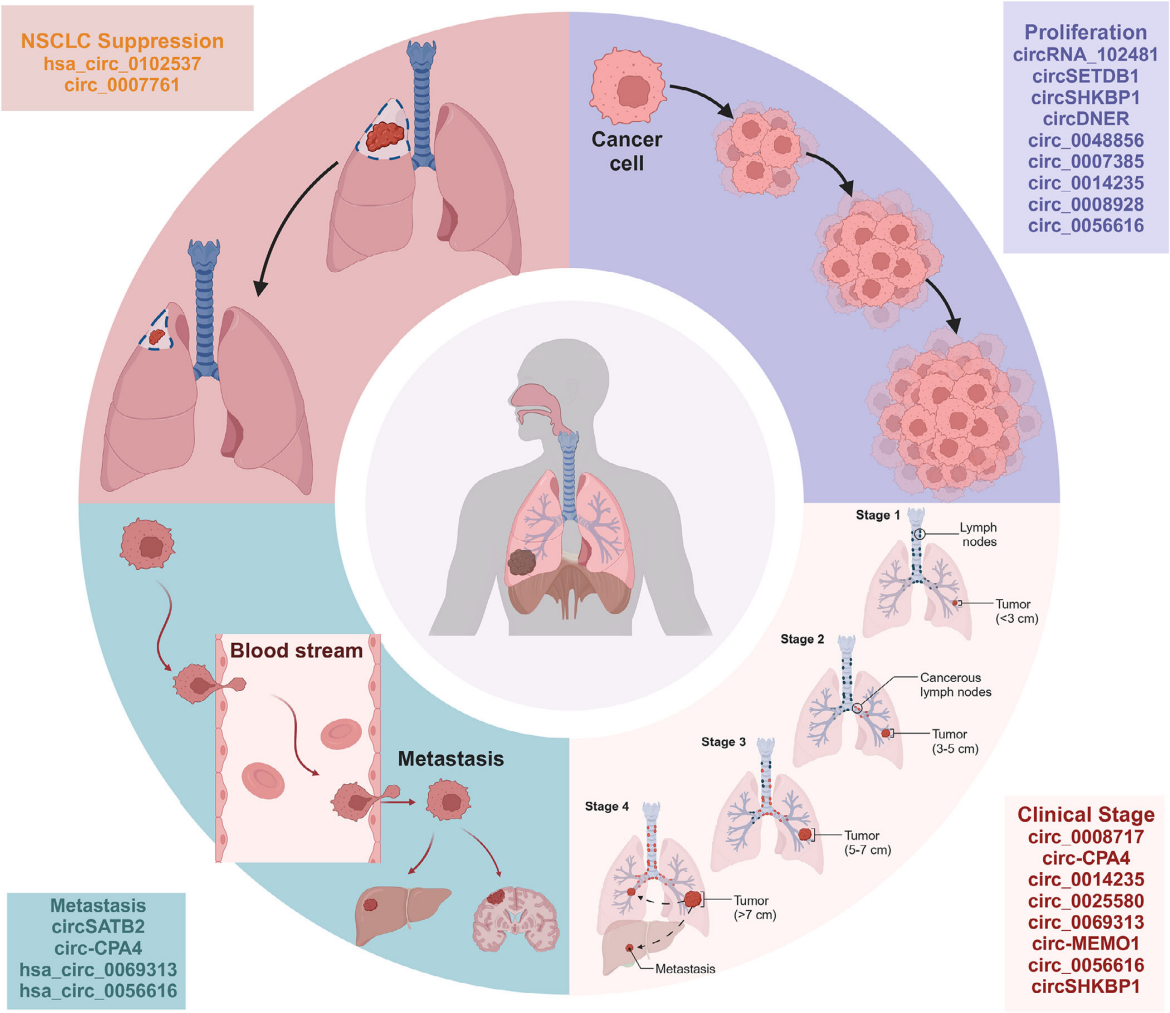
Representation of major biological activities of dysregulated exosomal circRNAs in NSCLC. This diagram illustrates the progression of NSCLC from normal lung tissue to advanced stages. The sequence begins with normal lung cells, which undergo proliferation and transformation into malignant cells, leading to the formation of small tumors that progressively enlarge and invade adjacent tissues. As the tumor expands, it disrupts local anatomical structures and has the potential to metastasize via the bloodstream and lymphatic system, ultimately reaching distant organs such as lymph nodes, bones, and internal viscera.

Continuous proliferation and evasion of apoptosis are characteristic changes in cancer cells.^647^ The specific biological implications of numerous circRNAs on NSCLC are yet unknown. ExocircRNAs are found to be abnormally expressed in body fluids, tissues, plasma, and serum in cases of NSCLC.^648^ This review outlines the research conducted to understand how exocircRNAs are related to NSCLC (Table 3). In recent years, exocircRNAs have been linked to the progression of NSCLC, specifically to proliferation, apoptosis, invasion, metastasis, angiogenesis, glycolysis in NSCLC cells, recurrence, mechanisms of treatment resistance, EMT in NSCLC cells, and mortality, by regulating their target miRNAs and downstream tumor‐associated signaling pathways^649^, ^650^ (Figure 8). Table 3 presents the positive correlation between several upregulated circRNAs and NSCLC progression. Additionally, the downregulation of hsa_circ_0102537 and circ_0007761 inhibits the progression of NSCLC.^651^

**TABLE 3:** The biological functions and potential clinical values of exosomal circular RNAs in NSCLC.

Exosomal circRNA	Target cell	Expression	Pathway	Potential clinical value	Function	References
circRNA_102481	PC9 HCC827	Up	miR‐30a‐5p/ROR1	Diagnostic biomarker and therapeutic target	Promote EGFR‐TKIs sensitive cell proliferation and inhibit cell apoptosis	^652^
circ_0008717	A549 H1299	Up	miR‐1287‐5p/PAK2	Therapeutic strategy	Promoted tumorigenesis in NSCLC	^653^
circSETDB1	A549 PC9	Up	miR‐7/Sp1	Occurrence and development	Enhances the proliferation, migration, and invasion of LUAD cells	^654^
circSHKBP1	A549 H1299	Up	miR‐1294/PKM2	Potential biomarker for the diagnosis and treatment of NSCLC	Promotes the proliferation, colony formation, migration, and invasion of NSCLC cells	^655^
hsa_circ_0102537	A549 H1975 H1299	Down	–	Therapeutic target	Inhibits the migration and invasion of LUAD cells	^651^
circ‐CPA4	A549 H1299	Up	let‐7/PD‐L1	Therapeutic agents	Regulated cell growth, mobility, stemness, and drug resistance in NSCLC cells	^650^
circ_0047921 circ_0056285 circ_0007761	–	UP Up Down	–	Diagnostic tool for NSCLC	Promising biomarkers for an NSCLC diagnosis	^656^
hsa_circ_0014235 hsa_circ_0025580 hsa_circ_0026403	–	UP UP UP	–	Potential biomarkers and therapeutic targets	Novel diagnostic biomarkers for LUSC	^657^
hsa_circ_0001492 hsa_circ_0001346 hsa_circ_0000690 hsa_circ_0001439	–	UP UP UP UP	–	Novel diagnosis and treatment biomarkers	Biomarkers and the pathological effects of lung cancer	^658^
circDNER	A549	UP	miR139‐5p/ITGB8	Potential prognostic biomarker and therapeutic target	Increased lung cancer cell proliferation, invasion and migration	^659^
circ_0048856	A549 H1299 SK‐MES‐1	UP	miR‐1287−5p	High diagnostic value for NSCLC	Facilitated proliferation, migration and invasion, inhibited apoptosis of NSCLC cells	^660^
hsa_circ_0069313	–	UP	NER	Discriminate NSCLC and benign lung tumor	The development of noninvasive biomarkers for more aggressive NSCLC	^661^
circ_0007385	A549 H1299	UP	miR‐1253/FAM83A	Diagnosis and treatment in NSCLC	Enhances non‐small cell lung cancer cell proliferation and stemness	^662^
circFARSA	A549 PC9	UP	PI3K/AKT	Diagnostic and prognostic biomarker	Increase in vitro migration and invasion	^649^
circ_PIP5K1A	A549 H1299	UP	miR‐101/ABCC1	Diagnosis and treatment in NSCLC	Promoted A549 and H1299 cell migration and invasion	^663^
circ‐MEMO1	A549 H1299	UP	MiR‐101‐3p/KRAS	Early diagnostic marker for NSCLC	Promotes the Progression and Aerobic Glycolysis of Non‐small Cell Lung Cancer	^664^
hsa_circ_0014235	A549 H1299	UP	miR‐520a‐5p/CDK4	Promising biomarker for NSCLC	Promoted DDP chemoresistance, leading to increased cell proliferation, migration and invasion	^665^
circ_0008928	A549 H1299	UP	miR‐488/HK2	Potential biomarker for the diagnosis	Knockdown suppressed cell proliferation, migration, invasion, and glycolysis metabolism, and improved CDDP sensitivity in CDDP‐resistant NSCLC	^666^
hsa_circ_0002130	HCC827 H1975	UP	miR‐498/GLUT1, HK2 and LDHA	Promotion role in osimertinib‐resistant NSCLC	Affected cell growth, apoptosis and glycolysis in NSCLC cells	^667^
hsa_circ_0000190	A549 H460 H1299	Up	miR‐142‐5p/PD‐L1	Therapeutic strategy of progression in NSCLC	Increased cell migration and invasion in NSCLC cells	^668^
hsa_circ_0056616	PC9 PC14	Up	–	Potential biomarker for theragnostic of lymph node metastasis in lung adenocarcinoma	Promoted colony formation frequency, proliferation rate, migration rate	^669^
hsa_circ_0000190 hsa_circ_0001649	A549 HCC827	Up	–	Valuable blood‐based biomarker to estimate the prognosis of lung cancer	Associated with clinicopathological features and the treatment response of LC patients	^670^
circSATB2	A549 H460	Up	miR‐326/FSCN1	Potential biomarker for clinical detection of NSCLC and metastatic NSCLC	Promote the progression of NSCLC, and may participate in cell‐to‐cell communication	^671^
circRNA‐002178	A549 PC9 95D	Up	miR‐34/PDL1/PD1	Novel diagnosis biomarker for LUAD	As a ceRNA to enhance PDL1 and PD1 expression in cancer cells and T cells	^672^
hsa_circ_0000519	H2170 A549	Up	miR‐1258/RHOV	A new insight into NSCLC development and treatment	Promoted the cell growth and metastasis in NSCLC	^673^
circPLK1			miR‐1294/HMGA1	Potential prognostic marker for NSCLC	Promoted NSCLC progression	^674^
circ‐IARS	A549 H1299	Up	miR‐1252‐5p/HDGF	A potential diagnostic biomarker for NSCLC and a potential target for the treatment of NSCLC	Promoted cell proliferation, migration, and invasion and inhibited cell apoptosis in NSCLC	^675^
circUSP7	A549 H1299	Up	miR‐934/SHP2	Promoted tumor progression and plays a role in immune evasion in NSCLC	Reduced the therapeutic efficacy of anti‐PD1 treatment in NSCLC	^676^
circRACGAP1	A549 H1299	Up	PTBP1/SIRT3/RIF1	The development of therapeutic strategies	A driver for stemness and metastasis of NSCLC	^677^
circ_0076305	A549 H292	Up	miR‐186‐5p/ABCC1	Regulating Cisplatin resistance of NSCLC	Potential circRNA‐targeted therapy for NSCLC	^678^
circARHGAP10	A549 H292	Up	miR‐638/FAM83F	Promoted proliferation, migration, invasion, and glycolysis of NSCLC cells	A theoretical basis for developing effective NSCLC diagnostic biomarkers and therapeutic targets	^679^
circVMP1	A549 H1299	Up	miR‐524‐5p‐METTL3/SOX2	Enhanced the proliferation, sphere formation, migration, invasion, and DDP resistance	Potential promising bio‐marker for NSCLC	^680^
circCD226	A549 H1299	Up	miR‐1224‐3p/HMGA2	Enhanced NSCLC cell proliferation, migration, invasion and stemness	A potential driver in NSCLC development	^681^

Abbreviations: ABCC1, ATP binding cassette subfamily C member 1; CDK4, cyclin‐dependent kinase 4; DDP/CDDP, cisplatin; FAM83A, family with sequence similarity 83 member A; FAM83F, family with sequence similarity 83 member F; FSCN1, fascin actin‐binding protein 1RHOV, RAS homolog family member V; GLUT1, glucose transporter 1; HDGF, hepatoma‐derived growth factor; HK2, hexokinase 2; HMGA1, high mobility group at‐hook 1; HMGA2, high mobility group AT‐hook 2; ITGB8, integrin beta8; KRAS, kirsten rat sarcoma viral oncogene homolog; LDHA, lactate dehydrogenase A; let‐7, let‐7 microRNA; METTL3/SOX2, methyltransferase Like 3/SRY‐Box Transcription Factor 2; NER, nucleotide excision repair; PAK2, p21‐activated kinase 2; PI3K/AKT, phosphoinositide 3‐kinase/protein kinase B; PKM2, pyruvate Kinase M2; PTBP1/SIRT3/RIF1, polypyrimidine tract‐binding protein 1/sirtuin 3/regulator of telomere lengths and telomere C‐strand synthesis; ROR1, receptor tyrosine kinase‐like orphan receptor 1; SHP2, SH2 domain‐containing phosphatase 2; Sp1, specificity protein 1.

ExocircRNAs target specific organs and cells by transporting exosomes, which subsequently play a role in controlling the proliferative, apoptotic, invasive, and metastatic processes of NSCLC cells. CircRNA_102481 promotes the proliferation of cells sensitive to EGFR‐TKIs while inhibiting their apoptosis by acting as a sponge for miR‐30a‐5p and downregulating ROR1 expression.^652^

Another study revealed an increase in hsa_circ_0008717 expression in serum exosomes from lung adenocarcinoma (LUAD) cases. Suppression of hsa_circ_0008717 decreased malignant behaviors in LUAD cells, arrested the cell cycle, and induced apoptosis, while repressing their proliferation, migration, and invasiveness, by modulating the miR‐1287‐5p/PAK2 axis.^653^

Similarly, circDNER expression is increased in exosomes from LUAD cells and cases. CircDNER acts as a sponge for miR‐139‐5p, while ITGB8 is the target of miR‐139‐5p. Consequently, circDNER enhances the ability of LUAD cells to proliferate and migrate by modulating the miR‐139‐5p/ITGB8 axis.^659^ Circ‐CPA4 is also involved in transport within LUAD cells. As discovered by Hong and colleagues, let‐7 is sponged by circ‐CPA4, while PD‐L1 is a target of let‐7. Mechanistically, circ‐CPA4 knockdown inhibited in vitro LUAD cell growth and suppressed in vivo LUAD tumor growth via the let‐7/PD‐L1 axis.^650^

Hsa_circ_0001346, hsa_circ_0000690, and hsa_circ_0001439 were found to be increased in lung cancer, presenting potential novel diagnostic and treatment biomarkers.^658^ Ning et al.^662^ discovered that overexpression of hsa_circ_0007385 enhances NSCLC cell proliferation and stemness via the miR‐1253/FAM83A signaling pathway. Circ_0048856 levels are increased in NSCLC cells and tissues, promoting proliferation, migration, and invasion, while inhibiting apoptosis in NSCLC cells.^660^ Additionally, hsa_circ_0102537 was predominantly packaged in exosomes, but less commonly found in LUAD tissues and plasma compared with normal subjects.^651^

##### ExocircRNAs target signaling pathways in NSCLC

**FIGURE 9:**
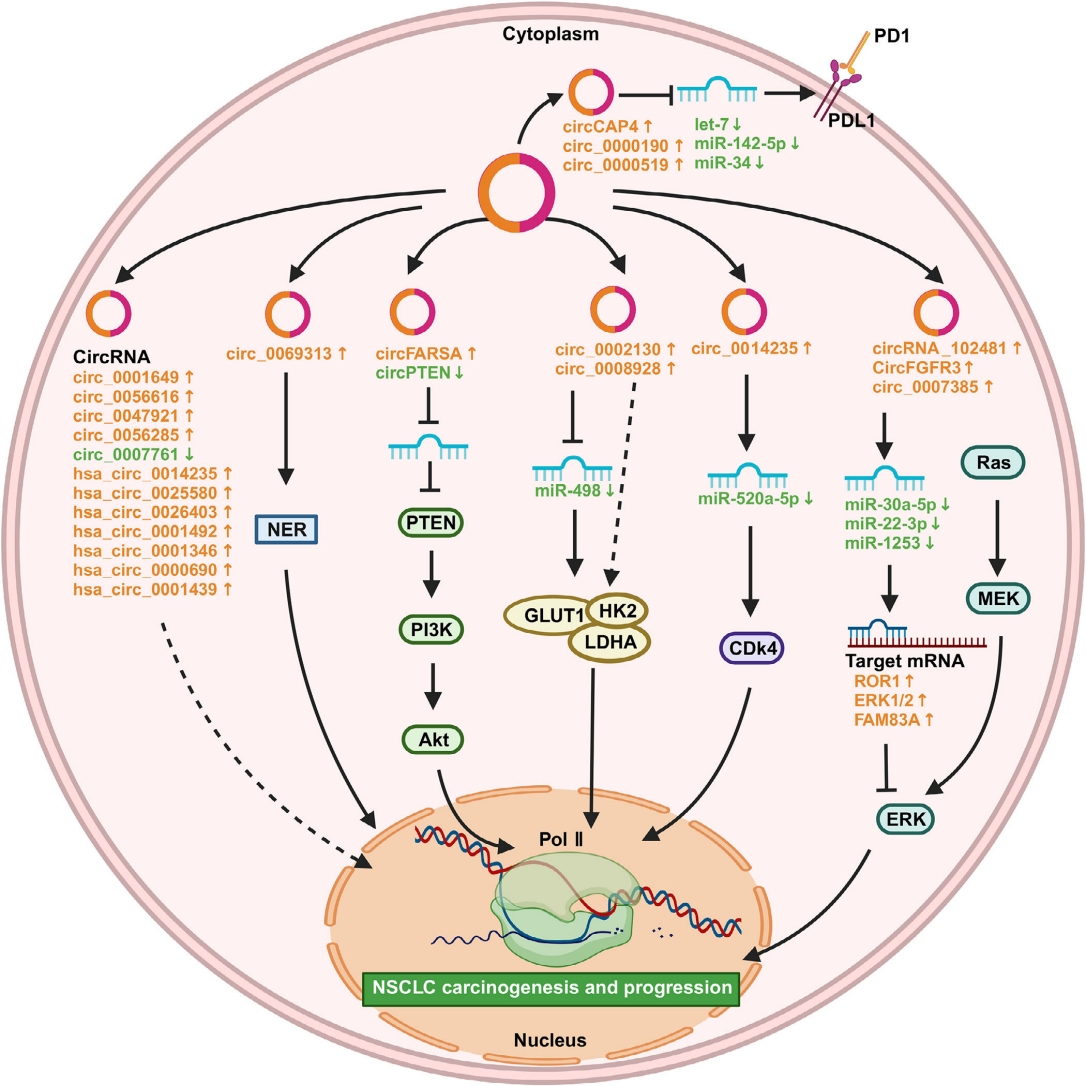
Regulation by circRNAs of signaling pathways in NSCLC. CircRNAs participate in the carcinogenesis and progression of NSCLC by regulating the expression of key components involved in cancer‐related signaling pathways.

An expanding volume of studies demonstrates that exocircRNAs contribute to the progression of NSCLC by influencing cancer‐related signaling pathways, specifically the PI3K/AKT and MAPK pathways. The PI3K/AKT signaling pathway holds significant importance in controlling diverse cellular biological processes such as cell cycle, glucose transport, and the development of cancer.^682^ Aberrations in the PI3K/AKT pathway are tightly linked to the advancement of NSCLC.^683^ Multiple exocircRNAs have been pinpointed as modulators of NSCLC tumorigenesis through their interaction with the PI3K/AKT signaling cascade. As an illustration, Chen and colleagues^649^ uncovered that exosomal circFARSA stimulates the PI3K/AKT pathway, bolstering the growth of NSCLC cell lines, specifically A549 and PC9. The MAPK signaling pathway is renowned for its role in cancer development. Disturbances in the MAPK/ERK cascade contribute significantly to various facets of cancer progression.^684^ Wang et al.^25^ revealed that circ‐ZKSCAN1 hinders the proliferation of NSCLC cells. This mechanism involves the upregulation of FAM83A expression by sequestering miR‐330‐5p, thereby suppressing the MAPK signaling pathway.^25^ Furthermore, reports indicate that hsa_circ_0004050 downregulates DUSP9 expression by sponging miR‐1233‐3p in the NSCLC cell line A549, thus impeding the ERK/JNK signaling cascade.^685^ Our investigations have demonstrated elevated levels of circ‐ZKSCAN1 in both NSCLC tissues and cell lines. This upregulation leads to the suppression of the MAPK signaling pathway by absorbing miR‐330‐5p, resulting in increased FAM83A expression and consequently, augmented NSCLC cell growth.^25^ Taken together, these findings underscore the pivotal role of circRNAs in regulating NSCLC signaling cascades (Figure 9).

##### Relations of exocircRNAs with NSCLC clinicopathological features

Presently, due to the intricate pathological traits of NSCLC, detecting its early symptoms is challenging. Consequently, many patients receive a diagnosis only when the disease has progressed to an advanced stage, thereby missing the optimal window for surgical intervention. Furthermore, the inadequate prognosis evaluation significantly impedes the customization of treatment plans for individual patients and efforts to extend their survival. In the past few years, several traditional biomarkers, including AFP, PD‐L1, and ALK, have been utilized for both the diagnosis and prognosis assessment of various cancers, NSCLC being one of them. Table 4 presents the relations of exocircRNAs with clinicopathological features in NSCLC cases. Wang and colleagues explored the relation of circ_0008717 level with clinicopathological features in 24 LUAD cases, revealing that LUAD cases who had circ_0008717 upregulation showed an advanced TNM and a large tumor size compared with those showing circ_0008717 downregulation.^653^ According to Hong et al.,^650^ exosomal circ‐CAP4 upregulation was tightly associated with TNM and extra‐lung metastasis (EM). As discovered by Xian et al.,^656^ upregulation of the serum markers hsa_circ_0025580 and hsa_circ_0014235 in LUAD patients was strongly linked to tumor sizes and TNM stages, which was not the case for sex, age, or smoking status. Moreover, exosomal circ_0047921 and circ_0056285 expression significantly increased among LUAD cases except circ_0007761 was decreased, which showed a positive relation to family history of cancer, but not with other clinical features.^656^ Furthermore, hsa_circ_0026403, hsa_circ_0025580, and hsa_circ_0014235 expression was also related to TNM stage and tumor sizes, rather than to sex, age, and smoking status.^657^ Moreover, hsa_circ_0069313 and Circ‐MEMO1 upregulation showed a positive relation to TNM, LNM as well as EM.^661^, ^664^ The expression of hsa_circ_0056616 showed a positive association with tumor size, TNM stages, and EM, which was not the case for age and sex.^669^ Based on the above evidence, tumor size, and TNM stage exhibited the highest association with aberrant exocircRNA expression whereas age, gender, number of tumors, smoking status, and LNM presented no significant relationship.

**TABLE 4:** Relationship between exocircRNAs and clinicopathologic characteristics in NSCLC.

circRNAs	Expression	N(H/L)	Gender	Age	TS	TNM	LNM	PT	SS	EM	References
circ_0008717	Up	24/24	–	–	Y	Y	–	–	–	–	^653^
circ‐CPA4	Up	25/25	N	N	–	Y	–	N	N	Y	^650^
hsa_circ_0014235	Up	15/15	N	N	Y	Y	–	–	N	–	^656^
hsa_circ_0025580	Up	15/15	N	N	Y	Y	–	–	N	–	^656^
hsa_circ_0069313	Up	108	–	–	N	Y	Y	N	–	Y	^661^
Circ‐MEMO1	Up	45/7	N	N	N	Y	Y	–	–	–	^664^
hsa_circ_0056616	Up	–	N	N	Y	Y	–	–	–	Y	^669^
circSHKBP1	Up	61/39	N	N	–	Y	Y	N	N	N	^655^
circSATB2	Up	44/15	N	N	–	N	Y	N	N	N	^671^
circPLK1	Up	25/25	N	N	N	Y	–	–	–	Y	^674^
circUSP7	Up	63/63	N	N	N	Y	Y	N	N	–	^676^

N, no supply; Y, supply.

##### ExocircRNAs’ significance in diagnosing and predicting the prognosis of NSCLC

Although great progresses have been made in surgical, locoregional, and medical treatments, NSCLC still has a high mortality rate due to disease metastasis and relapse postoperatively.^686^ NSCLC‐derived exosomes are found to redirect cancer cell metastasis in cells with no specific organ metastatic capacity.^650^ Exosomes can shuttle circRNAs between cells while regulating tissue genesis and cell differentiation. ExocircRNAs have been implicated in recent research as candidate prognostic and diagnostic biological markers for NSCLC.^651^, ^655^, ^661^ Table 5 presents the latest research on the involvement of exocircRNAs in diagnosing and predicting the prognosis of NSCLC.

**TABLE 5:** ExocircRNAs serve as potential diagnostic biomarkers for NSCLC.

CircRNAs	ROC	95 CI%	Sensitivity (%)	Specificity (%)	References
hsa_circ_0014235	0.8254	0.762–0.889	–	–	^657^
hsa_circ_0025580	0.8003	0.741–0.862	–	–	^657^
circ_0048856	0.943	0.904−0.982	88	80	^660^
hsa_circ_0069313	0.724	–	–	–	^661^
circ‐MEMO1	0.76	0.6259−0.8941	56.67	96	^664^
hsa_circ_0002130	0.792	0.676–0.909	–	–	^667^
hsa_circ_0056616	0.812	0.720–0.903	79.2	81	^669^
hsa_circ_0000190	0.95	0.926−0.974	90	90.2	^670^
circSATB2	0.66	0.581–0.74	–	–	^671^
circRNA‐002178	0.9967	–	–	–	^672^
circSHKBP1	0.853	–	–	–	^655^
circPLK1	0.866	–	–	–	^674^
circCD226	0.9571	–	–	–	^681^

In NSCLC individuals, there is an increase in the levels of hsa_circ_0025580 and hsa_circ_0014235, which may have an impact on NSCLC progression^657^ According to Wang and colleagues,^657^ hsa_circ_0014235 was a new marker to diagnose NSCLC, and its AUC value of the ROC curve value was 0.8254 (95 CI%: 0.762–0.889) to discriminate NSCLC.^657^ Moreover, hsa_circ_0025580 had 0.8003 (95 CI%: 0.741–0.862).^657^ According to He and coworkers,^660^ circ_0048856 showed upregulation in plasma‐derived exosomes with better diagnostic ability in NSCLC (AUC = 0.943, and the sensitivity and specificity were 88 and 80%, respectively). In addition, hsa_circ_0069313, circRNA‐002178, and circSHKBP1 have the ROC values of 0.724, 0.9967, and 0.853, respectively, to distinguish NSCLC from health, respectively.^655^, ^660^, ^661^ Moreover, Zhang et al.^671^ reported that circSATB2 was mostly released via exosomes in NSCLC cells, with significant upregulation in NSCLC cells and tissues compared with normal lung cells and para‐carcinoma tissues. According to the ROC curve, circSATB2 displayed great significance in diagnosing NSCLC, and the AUC was 0.66 (95 CI%: 0.581–0.74).^671^ As suggested by Ding and colleagues,^664^ circMEMO1 upregulation in nearby tissues predicted dismal survival for NSCLC cases.

Nevertheless, further study is required before circRNAs can be used in clinical settings since their function in NSCLC is still relatively unknown. Moreover, a large number of samples, multiple centers, and independent external cohort validation are required for circRNA to become a widely accepted biomarker. Besides, there are only a very few cirRNAs that have been found to have an impact on tumors. In addition, only a limited number of cirRNAs have been identified in NSCLC, and further research is required.

#### 4.3.2 ExocircRNAs and HCC

HCC arises from liver tissue and stands as a predominant cause of mortality worldwide, accounting for approximately 75−85% of primary liver cancers.^687^ HCC is intricately linked with various factors, including chronic hepatitis, cirrhosis, aflatoxin exposure, excessive alcohol consumption, and genetic predisposition.^688^ CircRNAs derived from HCC cells and their associated exosomes exert multifaceted influences on HCC progression, impacting processes such as tumor cell proliferation, metastasis, and apoptosis regulation. These circRNAs also hold promise as potential markers and therapeutic targets for HCC, examples of which include circTGFBR2,^689^ circ_002136,^690^ and hsa_circ_0074854.^691^ Conversely, certain exosome‐derived circRNAs have been identified as potential inhibitors of HCC, as evidenced by circRNAs like hsa_circ_0004658^692^ and circ_0051443,^626^ among others. In summary, exosomes play a pivotal and intricate role in the context of HCC, with exosome‐derived circRNAs serving as significant contributors. This role opens up novel perspectives and strategies for early HCC diagnosis and treatment. This section will delve into the specific role of exocircRNAs in HCC.

##### Exosome‐derived circRNA promotes HCC

Numerous studies have provided compelling evidence of the significant involvement of exosome‐derived circRNAs in advancing the progression of HCC. These specific circRNAs exert a substantial influence on the biological characteristics of HCC cells, including proliferation, metastasis, and drug resistance, thus actively fostering the advancement of HCC.

First, exocircRNAs derived from HCC cells can function as miRNA “sponges,” adsorbing miRNAs and thereby contributing to HCC progression. For example, circWHSC1 absorbs miR‐142‐3p and promotes proliferation and metastasis of HCC cells by directly targeting homeobox A1 (HOXA1), suggesting that circWHSC1 may become a diagnostic indicator of HCC.^693^ Circ_MMP2 interacts with miR‐136‐5p and MMP2, leading to the upregulation of MMP2 and the promotion of HCC cell progression. Elevated MMP2 levels are associated with HCC metastasis and poor overall survival in HCC patients.^694^ Similarly, circPTGR1 promotes cancer cell metastasis by competing for miR449a binding to MET.^632^ CircRNA Cdr1as promotes AFP expression by adsorbing miR‐1270.^624^ Circ_002136 augments Ras‐related expression by blocking miR‐19a‐3p, increasing Ras‐related protein Rab‐1A (RAB1A) expression and promotes HCC cell progression.^690^ CircTGFBR2 enhances ATG5‐mediated cytoprotective autophagy by interfering with the interaction between miR‐205‐5p and autophagy‐related protein 5 (ATG5), thereby promoting HCC progression.^689^ Notably, in arsenide‐induced HCC, circRNA_100284 can be transferred into normal hepatocytes and induce cell cycle acceleration, cell proliferation, and malignant transformation of normal hepatocytes by acting as a sponge for miRNA‐217, suggesting that circRNAs have the potential to induce malignant transformation in normal cells.^623^

Second, exocircRNAs may mediate drug resistance in HCC cells. For example, exosome‐derived circZFR from cancer‐associated fibroblasts (CAFs) is highly expressed in cisplatin (DDP)‐resistant HCC cell lines and can inhibit the STAT3/NF‐κB pathway, promote tumor growth and enhance DDP resistance. Targeting circZFR and related pathways may help improve the efficacy and survival of patients treated with chemotherapy for HCC.^695^ Exosome‐derived circRNA‐SORE from HCC cells is frequently upregulated in sorafenib‐resistant HCC cells, and further studies have shown that circRNA‐SORE stabilizes the cytoplasmic oncogenic protein YBX1, which is a key component in the development of HCC. Silencing of circRNA‐SORE by siRNA could overcome sorafenib resistance in HCC patients.^696^ CircPAK1 derived from HCC cell exosomes promotes HCC cell proliferation, invasion, and metastasis, while inhibiting apoptosis and angiogenesis. Further studies showed that exosomes from ravastatinib‐resistant HCC cells mediate the transport of circPAK1, contributing to ravastatinib resistance.^697^

In addition, HCC‐derived exocircRNAs can inhibit immune cells in the TME and promote angiogenesis to exert procancer effects. For example, circUHRF1 promotes tumor lung metastasis and reduces the number of natural killer group 2, member D (NKG2D)‐positive cells in metastatic tumor nodules and contributes to NK cell dysfunction and poor clinical prognosis in HCC patients.^698^ Hsa_circ_0074854 regulates the expression of HCC cells by interacting with HuR protein and regulating its protein expression level, thereby affecting the EMT process in HCC cells. Conversely, exosomes carrying downregulated hsa_circ_0074854 can translocate to macrophages and inhibit their polarization, which may ultimately impede the migration, invasion, and EMT of HCC cells.^691^ CircFUT8, whose expression is upregulated in HCC cells, can be modified by m6A, thereby promoting HCC progression. However, M1‐type macrophage‐derived exosomes can inhibit HCC progression by transfecting miR‐628‐5p into HCC cells and downregulating m6A modification, suggesting an anticancer role for M1‐type macrophage‐derived exosomes.^699^ CircGSE1 can maintain the expression of transforming growth factor‐beta receptor 1 (TGFBR1) and Smad3, which promotes the expansion of Treg cells and thereby inhibits the activity of effector T cells, such as CD8^+^ T cells, resulting in immune escape from HCC cells.^700^ In addition, circTMEM181 increases CD39 expression on macrophages and inhibits CD8^+^ T cells, leading to resistance to PD‐1 treatment in HCC.^701^ Similarly, circCCAR1 is able to promote CD8^+^ T cell exhaustion, leading to impaired antitumor immunity in TME. The mechanism is that circCCAR1 prevents the ubiquitinated degradation of the PD1 protein by binding directly to PD1, thereby impairing the tumor killing capacity of CD8^+^ T cells^702^ Notably, in vitro and animal studies have shown that circRNA‐100338 promotes the invasion and metastasis of HCC cells and promotes vascular endothelial cell proliferation, angiogenesis, and which correlates with lung metastasis and reduced survival in HCC patients.^63^

##### Exosome‐derived circRNA inhibits HCC

Conversely, it has also been shown that exosome‐derived circRNAs can inhibit HCC progression by adsorbing miRNAs. For example, circ_0051443 can act as a sponge for miR‐331‐3p, thereby attenuating the regulatory effect of miR‐331‐3p on BCL2 antagonist/killer 1 (BAK1), which inhibit HCC cell progression.^626^ Macrophage exosomes overexpressing recombination signal binding proteinJκ (RBPJ) overexpress hsa_circ_0004658, which led to the upregulation of junctional adhesion molecule 3 through the adsorption of miR‐499b‐5p, thereby inhibiting HCC cell proliferation and promoting apoptosis, suggesting the diagnostic and therapeutic potential of hsa_circ_0004658 as a biomarker and target for HCC cell therapy.^692^

Taken together, these data demonstrate the important role of exosome‐derived circRNAs in promoting/inhibiting HCC development, mediating drug resistance, promoting proliferation, metastasis, and regulating immune escape. These findings provide important clues for understanding the mechanism of exosome‐derived circRNAs in the pathophysiology of HCC cells and offer new opportunities for their clinical application as diagnostic and prognostic markers as well as therapeutic targets in HCC cells.

#### 4.3.3 ExocircRNAs and CRC

CRC is a malignant tumor, that starts in the lining of the colon or rectum. It is the third most common cancer worldwide, after lung and breast cancer, and the second leading cause of cancer death after lung cancer.^703^ Risk factors for CRC include a diet high in processed meat, obesity, smoking, low fruit and vegetable intake, and a history of inflammatory bowel disease.^704^ Patients with hereditary nonpolyposis colorectal cancer or familial adenomatous polyposis and relatives with a positive family history of CRC are also at increased risk.^705^ Several studies have found that circRNAs in exosomes are strongly associated with CRC. These circRNAs may have an important impact on cancer cell proliferation, migration, invasion and resistance to radiotherapy and drugs by regulating the expression of tumor‐related genes or modulating signaling pathways, and these findings provide new insights into the mechanism of action of circRNAs in exosomes, as well as clues in the search for biomarkers and new therapeutic targets for CRC. This section focuses on these studies.

##### Exosome‐derived circRNA promotes CRC

CircRNAs in CRC tissues can block downstream signaling pathways by adsorbing miRNAs, thereby affecting cancer cell growth, metastasis and drug resistance.

First, exosome‐derived circRNAs in CRC cells can promote tumor invasion, and metastasis by promoting endothelial cell migration and proangiogenesis. For example, circ_001422 was able to inhibit the expression of miR‐195‐5p, which in turn promotes the kinase insert domain receptor (KDR)/mTOR signaling pathway and facilitates endothelial cell migration and angiogenesis, suggesting that circ_001422 has a role as a biomarker in the diagnosis of CRC.^706^ CircTUBGCP4 activates AKT through regulation of the miR‐146b‐3p pathway, thereby promoting vascular endothelial cell migration, lumen formation and tumor metastasis, providing important clues to understand the molecular mechanisms of CRC development.^707^ Second, cancer cell exosome‐derived circRNAs can attenuate the sensitivity of CRC cells to radiation, resulting in a reduced effect of radiotherapy. For example, circ_0067835 interacts with miR‐296‐5p and exerts a procancer effect by regulating the miR‐296‐5p/insulin‐like growth factor 1 receptor (IGF1R) pathway in cancer cells. When downregulated in vivo, circ_0067835 inhibited CRC growth and increased cellular sensitivity to radiation.^708^ In addition, circ_IFT80 can target miR‐296‐5p and further inhibit musashi1 (MSI1), a gene associated with tumor progression and radiosensitivity, thereby promoting tumorigenesis and reducing cellular sensitivity to radiation.^709^ In addition, exosome‐derived circRNAs can also promote tumorigenesis under hypoxic conditions. For example, under hypoxic conditions, CAFs exosomes can secrete circEIF3K, which in turn promotes CRC cell proliferation, invasion and metastasis. The miR‐214/PD‐L1 pathway plays an important role in this process.^710^ The hypoxic CRC cell‐derived exosome circ‐133 is able to translocate to normal tumor cells and promote migration via the miR‐133a/guanine nucleotide exchange factor‐H1 (GEF‐H1)/RhoA axis, suggesting a difference in metastatic potential between hypoxic and relatively normoxic cancer cells.^711^

In terms of drug resistance, circRNAs may act as miRNA sponges to mediate CRC resistance to chemotherapeutic agents. For example, circ_0006174‐enriched exosomes from doxorubicin (DOX)‐resistant CRC cells promoted DOX resistance by binding miR‐1205 and upregulating cyclin D2 (CCND2). This finding has important clinical implications for the treatment of CRC patients and is expected to be a future target for diagnosis and therapy.^712^ The exosome‐derived hsa_circ_0005963 of oxaliplatin‐resistant CRC cells enhances cellular resistance to chemotherapeutic agents by adsorbing miR‐122, leading to overexpression of the M2 isoform of pyruvate kinase (PKM2), which subsequently increases cellular glycolytic metabolism. Targeting circRNA with siRNA (si‐ciRS‐122) can inhibit glycolysis and reverse resistance to oxaliplatin.^636^ In addition, increased expression of circ_0000338 in CRC cells correlates with increased 5‐FU resistance. It has been shown that circ_0000338 negatively regulates the expression of miRNAs miR‐217 and miR‐485‐3p by interacting with these two miRNAs, leading to 5‐FU resistance in CRC patients.^713^ Notably, some exosome‐derived circRNAs are also capable of encoding proteins, thereby mediating drug resistance. For example, circATG4B is expressed at elevated levels in exosomes released from drug‐resistant CRC cells and is capable of encoding the protein circATG4B‐222aa. CircATG4B‐222aa competitively interacts with the membrane vesicle transport protein transmembrane p24‐trafficking protein 10 (TMED10) and acts as a drug resistance blocker. This competitive interaction with transmembrane p24‐trafficking protein 10 (TMED10), acting as a decoy to prevent TMED10 from binding to autophagy‐related gene 4B (ATG4B), an interaction that leads to increased autophagy in CRC cells, thereby inducing the development of chemotherapeutic resistance.^714^

##### Exosome‐derived circRNAs inhibit CRC

In contrast, the effect of circRNAs on CRC also has therapeutic potential, and certain exosome‐derived circRNAs are also able to inhibit CRC, providing an important basis for clinical practice and the development of therapeutic strategies.

It has been suggested that adsorption of miRNAs by circRNAs may also inhibit CRC. For example, circFNDC3B in CRC cell lines and exosomal samples is able to adsorb miR‐937‐5p and thus inhibit its function. In contrast, miR‐937‐5p is known to promote EMT. In animal studies, overexpression or exosomal treatment of circFNDC3B was shown to inhibit the growth, angiogenesis and liver metastasis of CRC transplant tumors.^715^ CircEPB41L2 acts as a sponge for miR‐215p and miR‐9425p in exosomes, and overexpression of circEPB41L2 inhibits CRC cell proliferation, enhances apoptosis, and inhibits migration and invasion, which subsequently blocks the regulation of PTEN/AKT signaling pathway by these two miRNAs, ultimately presenting an inhibitory effect on CRC progression in cells and animal models.^716^

In addition, some circRNAs act as protein decoys capable of binding proteins to exert inhibitory effects. For example, the plasma exosome circLPAR1 can be internalized by CRC cells and inhibits METTL3–eukaryotic translation initiation factor 3 subunit h (eIF3h) through binding to eIF3h, thereby inhibiting mRNA translation, which in turn inhibits the expression of the oncogene bromodomain‐containing protein 4 (BRD4), thereby suppressing CRC cell proliferation, invasion and migration. Clinical studies have shown that downregulation of circLPAR1 is associated with poor survival in CRC patients. Therefore, circLPAR1 may serve as a potential diagnostic marker for CRC.^62^

Some circRNAs have oncogenic effects in cancer cells, but tumor cell exosomes excrete them, thus escaping their inhibitory effects and maintaining cancer cell adaptation. An example is the exosome‐derived circRHOBTB3 in CRC cells. in response to this situation, researchers have proposed to intervene in the secretion process of circRHOBTB3 through antisense oligonucleotides, thereby increasing its intracellular expression and consequently enhancing its inhibitory effect on CRC.^717^

Notably, it has been shown that some circRNAs not only inhibit tumors but also promote resistance to chemotherapeutic agents. For example, hsa_circ_0000338 has a tumor growth inhibitory effect in CRC cells, but its delivery to other CRC cells via exosomes leads to a role in promoting drug resistance. When knockdown of hsa_circ_0000338 was targeted by siRNA, it was shown to enhance drug resistance, suggesting that hsa_circ_0000338 may have a tumor growth inhibitory effect in CRC cells. These findings provide important clues to further understand the function of circRNAs.^635^ More studies on exosome‐derived circRNAs in CRC have been presented in Table 6.

**TABLE 6:** Expression of exosome‐derived circRNA in CRC.

circRNAs	Samples	Regulation	Signaling pathway	Function	References
circ_001422	Tissues; blood; CRC cells	Upregulated	miR‐195‐5p/KDR/mTOR	Proangiogenesis	^706^
circTUBGCP4	Tissues; blood	Upregulated	miR‐146b‐3p/AKT	Promotes angiogenesis and tumor metastasis	^707^
circ_0067835	Tissues; CRC cells	Upregulated	miR‐296‐5p/IGF1R	Promotes CRC development and enhances radioresistance	^708^
circ_IFT80	Blood; CRC cells	Upregulated	miR‐296‐5p/MSI1	Promotes tumorigenesis and reduces radiosensitivity	^709^
circEIF3K	Tissues; cells	Upregulated	miR‐214/PD‐L1	Induces CRC progression	^710^
circ‐133	Tissues; Blood; CRC cells	Upregulated	miR‐133a/GEF‐H1/RhoA	Promotes cell migration	^711^
circ_FMN2	Blood; CRC cells	Upregulated	miR‐338‐3p/MSI1	Accelerates CRC progression	^718^
circPACRGL	CRC cells	Upregulated	miR‐142‐3p/TGF‐β1, miR‐506‐3p/TGF‐β1	Enhances CRC cell proliferation, migration and invasion, as well as differentiation of N1–N2 neutrophils	^719^
circCOG2	Blood; tissues; CRC cells	Upregulated	miR‐1305/TGF‐β2/SMAD3	Promotes CRC proliferation, migration, and invasion	^720^
circLONP2	Tissues; CRC cells	Upregulated	circLONP2/DDX1/pri‐miR‐17	Enhances the invasiveness of CRC cells	^721^
circCOL1A2	Tissues; CRC cells	Upregulated	miR‐665/LASP1	Promotes CRC cell proliferation, migration, invasion, and EMT	^722^
circFMN2	Blood; tissues; CRC cells	Upregulated	miR‐1182/hTERT	Promotes CRC progression	^723^
circPABPC1	Tissues; CRC cells	Upregulated	miR‐874 or miR‐1292/ADAM19 or BMP4	Promotes CRC liver metastases	^724^
circ‐RNF121	Tissues; CRC cells	Upregulated	miR‐1224‐5p/FOXM1	Promotes CRC cell proliferative and metastatic ability	^725^
circ‐HMGCS1	Blood; colon cancer cells	Upregulated	miR‐34a‐5p/SGPP1	Enhances colon cancer cell viability and invasion and suppress apoptosis	^726^
circ_0006174	Tissues; CRC cells	Upregulated	miR‐1205/CCND2	Enhances DOX resistance	^712^
hsa_circ_0005963	Tissues; CRC cells	Upregulated	miR‐122/PKM2	Reduces drug susceptibility in chemosensitive cells	^636^
circ_0000338	Tissues; CRC cells	Upregulated	miR‐217/AEG‐1;miR‐485‐3p/TPX2	Enhances chemotherapy resistance	^713^
circATG4B	Tissues; CRC cells	Upregulated	TMED10/ATG4B	Participates in the decreased chemosensitivity of CRC cells	^714^
circLPAR1	Tissues; CRC cells	Downregulated	eIF3h/BRD4	Internalized by CRC cells, and suppresses tumor growth	^62^
circFNDC3B	Tissues; CRC cells	Downregulated	miR‐937‐5p/TIMP3	Inhibits tumorigenic, metastatic, and angiogenic properties of CRC	^715^
circEPB41L2	Blood; CRC cells	Downregulated	miR‐21‐5p/PTEN/AKT; miR‐942‐5p/PTEN/AKT	Restrains CRC progression and the activity of PTEN/AKT signaling pathway	^716^
circRHOBTB3	Tissues	Downregulated	NF‐κB, RAS, and ERK	Represses metabolic pathways and intracellular ROS production in CRC	^717^
hsa_circ_0000338	Blood; CRC cells	Downregulated	Wnt; cGMP‐PKG	May have dual regulatory roles in chemo‐resistant CRC	^635^

Abbreviations: ADAM19, a disintegrin and metalloproteinase‐domain‐containing protein 19; AKT, protein kinase B; ATG4B, autophagy‐related gene 4B; BMP4, bone morphogenetic protein 4; BRD4, bromodomain‐containing protein 4; CCND2, cyclin D2; cGMP‐PKG, cyclic guanosine monophosphate‐dependent protein kinase; DDX1, DEAD box 1; eIF3h, eukaryotic translation initiation factor 3 subunit h; ERK, extracellular signal‐regulated kinases; FOXM1, forkhead box M1; GEF‐H1, Rho guanine nucleotide exchange factor; HOXB7, Homeobox B7; hTERT, human telomerase reverse transcriptase; IGF1R, insulin‐like growth factor 1 receptor; KDR, kinase insert domain receptor; LASP1, multifunctional LIM and SH3 protein 1; MSI1, musashi1; mTOR, mammalian target of rapamycin; NF‐κB, nuclear factor kappa‐light‐chain‐enhancer of activated B cells; PKM2, M2 isoform of pyruvate kinase; PTEN, phosphatase and tensin homolog; RAS, rat sarcoma; SGPP1, sphingosine‑1‑phosphate phosphatase 1; SMAD3, mothers against decapentaplegic homolog 3; TGF‐β1, transforming growth factor beta 1; TIMP3, tissue inhibitor of metalloproteinase 3; TMED10, transmembrane p24‐trafficking protein 10; TPX2, targeting protein for xenopus kinesin‐like protein 2; Wnt, wingless‐related integration site.

In summary, exosome‐derived circRNAs have important roles in the development and treatment of CRC. These studies provide potential therapeutic targets and theoretical bases by deeply exploring the functions of circRNAs in exosomes, which provide important clues for future clinical practice and the development of therapeutic strategies. However, the different effects of circRNAs further suggest that researchers need to consider their mechanisms of action and regulatory networks more comprehensively in order to better understand their roles in disease development and provide more accurate targets and pathways for future therapeutic strategies.

#### 4.3.4 ExocircRNAs and pancreatic cancer

Pancreatic cancer is a type of cancer caused by the growth of malignant cells in the pancreatic tissue and is the fourth most common tumor in the world.^727^ The most common type of pancreatic cancer is PDAC, which accounts for 90% of pancreatic cancer cases.^728^ Studies have shown that the median survival of patients with pancreatic cancer who do not undergo surgical resection is approximately 2−8 months, and that approximately 94% of patients survive less than 5 years after diagnosis.^729^ The main risk factors for pancreatic cancer include smoking, obesity, high‐fat diet and chronic pancreatitis.^730^ Because pancreatic cancer is not obvious in its early symptoms and is often detected at an advanced stage, it is more difficult to treat and has a poorer prognosis.^731^ Some studies have found that circRNAs in exosomes are closely associated with pancreatic cancer. These circRNAs may have important effects on cancer cell proliferation, migration, invasion and drug resistance.

First, circRNAs exert oncogenic effects by adsorbing miRNAs and thereby blocking downstream signaling pathways. A study of 85 PDAC tissues, plasma exosomes, pancreatic cancer cells and human microvascular endothelial cells showed that circ‐IARS expression was upregulated in plasma exosomes from pancreatic cancer tissues and patients with metastatic disease, which could enter endothelial cells via exosomes and promote tumor invasion and metastasis. Overexpression of circ‐IARS significantly downregulated miR‐122 and zonula occludens‐1 (ZO‐1) levels, upregulated RhoA and RhoA‐GTP levels, increased filamentous actin (F‐actin) expression and adhesive plaques, and increased endothelial monolayer permeability to promote tumor invasion and metastasis.^633^ In addition, a study of liver metastasis specimens from PDAC patients showed that high expression of the plasma exosome circ‐PDE8A was associated with lymphatic invasion, TNM stage and poorer survival in PDAC patients. Further studies found that circ‐PDE8A acted as a sponge for miR‐338 and stimulated invasive growth through the MACC/MET/ERK or AKT pathways.^630^ In addition, the exosome circPDK1 significantly promotes pancreatic cancer cell proliferation, migration and glycolysis both in vitro and in vivo and is associated with poor patient survival. Mechanistically, circPDK1 can be activated at the transcriptional level by HIF‐1 alpha and sponge miR‐628‐3p, thereby activating the bromodomain PHD finger TF (BPTF)/c‐MYC axis. In addition, circPDK1 can act as a scaffold to enhance the interaction of ubiquitin conjugating enzyme E2O (UBE2O) with bridging integrator 1 (BIN1) and induce UBE2O‐mediated degradation of BIN1.^732^ In addition, hsa_circ_0000069 was significantly upregulated in exosomes derived from pancreatic cancer cells, which could act as a sponge for miR‐144 and thus promote tumor progression. miR‐144 targets the SCL/TAL1 interrupting locus (STIL), and knockdown of hsa_circ_0000069 significantly reduced the expression of STIL, CDK1, and cyclin B1 expression. Thus, downregulation of hsa_circ_0000069 induces pancreatic cancer cell cycle arrest in the G2‐M phase.^733^

Second, exosome‐derived circRNAs promote drug resistance in pancreatic cancer cells. For example, it has been shown that exosomes derived from pancreatic cancer cells in a hypoxic environment enhance the resistance of normoxic pancreatic cancer cells to gemcitabine. The mechanism is that the hypoxia‐induced exosome circZNF91 can be transfected into normoxic pancreatic cancer cells and competitively binds to miR‐23b‐3p, which leads to the upregulation of sirtuin 1 (SIRT1) and thereby enhances the deacetylation‐dependent stability of the HIF‐1α protein, resulting in the resistance of pancreatic cancer cells to GEM. Thus, the exosome circZNF91 may serve as a potential therapeutic target.^734^

Conversely, some exosome‐derived circRNAs may also inhibit pancreatic cancer progression and impede immune escape of tumor cells, thereby exerting anticancer effects. For example, the bone marrow MSC (BM‐MSC)‐derived exosome hsa_circ_0006790 promotes the nuclear translocation of chromobox homolog 7 (CBX7), which recruits DNA methyltransferase to the promoter region of S100A11, thereby increasing DNA methylation and inhibiting S100A11 transcription. Inhibition of CBX7 or overexpression of S100A11 eliminates the inhibitory effects of hsa_circ_0006790 on PDAC growth, metastasis and immune escape.^735^ In addition, BM‐MSC‐derived exosomes significantly reduced pancreatic cancer cell invasion, migration, and proliferation, as well as tumor stemness. Further studies showed that circ_0030167 mainly regulates miR‐338‐5p, increased Wnt inhibitory factor 1 expression and inhibited the Wnt8/β‐catenin signaling pathway, thereby suppressing PC cell stemness and tumor progression.^736^ Overexpression of serum‐derived exosome hsa_circ_0012634 targets MiR‐147b, thereby inhibiting homeodomain‐interacting protein kinase 2, suppressing PDAC cell growth and glycolysis in vitro and inhibiting tumorigenesis in vivo.^737^

Taken together, the discovery of these exosome‐derived circRNAs provides new perspectives in pancreatic cancer research and reveals important mechanisms associated with cancer development and treatment. As research in this area continues, there is a clearer understanding of how circRNAs can be used for their potential role in the treatment and diagnosis of pancreatic cancer. These findings are promising for further research and clinical applications.

#### 4.3.5 ExocircRNAs and prostate cancer

Prostate cancer is a malignant tumor that develops in the prostate tissue, usually from prostate epithelial cells, and is a common malignancy of the male genitourinary system.^738^ Some studies have shown that the incidence is higher worldwide in regions such as North America, Europe, and Oceania.^739^ With demographic and lifestyle changes, the incidence of prostate cancer is also gradually increasing in some developing countries.^740^ Early‐stage prostate cancer usually has no obvious symptoms, so regular physical examinations and screening are crucial for early detection and treatment. Treatment options include surgery, radiotherapy, chemotherapy and endocrine therapy.^741^ Fortunately, many prostate cancers can be controlled with early detection and effective treatment. Chemoresistant prostate cancer (CRPC) is a type of prostate cancer that develops after androgen deprivation therapy, which stops the growth of male sex hormones on prostate cancer cells. However, sometimes prostate cancer cells gradually become resistant to this treatment, leading to the development of depot‐resistant prostate cancer.^742^ Several studies have found that circRNAs in exosomes are closely associated with prostate cancer development. These circRNAs may have important effects on cancer cell proliferation, migration, invasion and drug resistance. They are highlighted in the following section.

First, in prostate cancer cells, circRNAs can act as sponges for miRNAs and thus act as inhibitors of downstream signaling pathways. For example, in myeloid‐derived suppressor of cellular exocytosis (MDSC‐Exo)‐treated PC3 cells, MDSC‐Exo accelerated PCa cell proliferation, migration, and invasion, whereas circMID1 defects inhibited MDSC‐Exo‐regulated ex vivo CRPC progression. Mechanistically, MDSC‐derived exosome S100A9 adsorbed miR‐506‐3p by upregulating circMID1 expression, leading to increased midline 1 (MID1) expression and accelerated tumor progression. The results suggest that the S100A9/circMID1/miR‐506‐3p/MID1 axis is present in the MDSC‐exo‐regulated progression of castration‐resistant prostate cancer (CRPC).^743^ In addition, circ_0044516 was significantly upregulated in both exosomes and cell lines from prostate cancer patients. Further studies showed that circ_0044516 promoted proliferation and metastasis of prostate cancer cells. The mechanism is that circ_0044516 can downregulate the expression of miR‐29a‐3p in prostate cancer, thus playing an important role in the survival and metastasis of prostate cancer cells.^744^

Second, exosome‐derived circRNAs also promote drug resistance in prostate cancer cells. Circ‐XIAP was found to be overexpressed in the exosomes of docetaxel (DTX)‐resistant prostate cancer cells and could be delivered via exosomes. Knockdown of circ‐XIAP increased DTX sensitivity by inhibiting proliferation, migration and invasion of DTX‐resistant cells and inducing cell cycle arrest and apoptosis. The mechanism is that circ‐XIAP can directly target miR‐1182 and subsequently promote multidrug resistance in prostate cancer by regulating the miR‐1182/TPD52 axis, which is a promising therapeutic target for prostate cancer chemotherapy.^745^

In addition, exosome‐derived circRNAs may act as protein decoys for cancer‐promoting effects. One study showed that exosome‐derived circTFDP2 expression was upregulated in prostate cancer tissues and promoted prostate cancer cell proliferation, and metastasis in vitro and in vivo. Mechanistically, circTFDP2 interacts with poly ADP ribose polymerase 1 (PARP1) protein in its DNA‐binding domain to block its active caspase‐3‐dependent cleavage, ultimately alleviating DNA damage in PCa cells. In addition, the RBP eIF4A3 can interact with the flanking region of circTFDP2 to promote circTFDP2 production.^746^

In conclusion, exosome‐derived circRNAs play an important role in prostate cancer development and further studies will help to better understand their role in prostate cancer pathogenesis and provide more precise targets and pathways for future treatment.

#### 4.3.6 ExocircRNAs and breast cancer

ExocircRNAs have been identified as key regulators in cancer progression and carcinogenesis.^747^, ^748^ These circRNAs can distinguish between different breast cancer subtypes, providing quick guidance on the appropriate therapy protocol for patients, thereby contributing to improved outcomes. ExocircRNAs that have prognostic value may also have therapeutic applications; silencing their expression or targeting them therapeutically could enhance tumor prognosis.^749^ The stability of circRNAs is due to the absence of polyadenylated tails and 5′/3′ polarities.^750^ By using alternative splice sites and various splicing mechanisms, circRNAs can directly influence the transcription of linear isoforms. Additionally, circRNAs can regulate transcription by inducing DNA hypomethylation in the promoter region of the parental gene or by modulating intronic enhancers. For instance, FECR1 circRNA interacts with the FLI1 promoter through significant demethylation, thereby regulating the FLI1 gene, which promotes breast cancer growth and metastasis.^751^ The role of the circRNA‐related ceRNA network in breast cancer development is crucial and requires comprehensive examination.^752^ A microarray study revealed that circTADA2A‐E6 acts as a tumor suppressor by targeting SOCS3 as a downstream gene, while another circRNA, circTADA2A‐E5/E6, is downregulated in breast cancer.^753^ Another study reported that circ_000911 also functions as a tumor suppressor.^754^ Breast cancer is linked with the upregulation of circEPSTI1, a prognostic marker and mediator in TNBC.^755^ In TNBC cases with lymph node metastases and advanced clinical stages, circANKS1B is elevated, which inhibits cancer metastasis.^591^

Exosomes have the potential to serve as biomarkers for determining cancer cell proliferation.^756^ The expression profile of exosomes and cells from the MDA‐MB‐231 cell line revealed changes in gene expression. Research by Lin et al.^757^ identified nine circRNAs in plasma EVs that were upregulated, including hsa_circ_0005552, hsa_circ_0007177, hsa_circ_0002190, hsa_circ_0001439, hsa_circ_0000642, hsa_circ_0006404, hsa_circ_0000267, hsa_circ_0001073, and hsa_circ_0001417. Five circRNAs‐hsa_circ_0009634, hsa_circ_0020707, hsa_circ_0064923, hsa_circ_0104852, and hsa_circ_0087064‐were found to be upregulated in exosomes derived from highly aggressive cells and metastatic tumors in breast cancer patients.^758^ Despite a limited number of investigations on exocircRNA in breast cancer, emerging knowledge in this field may soon provide alternative therapeutic options. Studies on chemoresistant breast cancer have shown that circRNAs might play a role in metastasis or transformation. For instance, exosome‐transmitted circHIPK3 induces trastuzumab resistance in initially drug‐sensitive breast cancer cells.^759^ Though there are few studies on circRNAs and chemoradiation resistance, circRNAs could be novel biomarkers for evaluating chemotherapy efficacy and predicting recurrence in drug‐resistant cancers. Studies indicate that in vitro transduction of circ‐UBE2D2 via exosomes can enhance resistance to tamoxifen in breast cancer, with recent findings showing circ‐UBE2D2 expression being 20‐fold higher in MCF‐7/TAM‐R‐Exo compared with MCF‐7/Par‐Exo.^760^

#### 4.3.7 ExocircRNAs and GC

A set of exocircRNAs exhibit abnormal expression patterns in GC cells and tissues, thereby playing pivotal roles in the initiation and advancement of GC. CircRNAs possess remarkable stability and extended half‐life in bodily fluids because of their resistance to exonucleases and ribonucleases. The expression levels of exocircRNAs frequently demonstrate elevated sensitivity and specificity throughout different GC stages.^761^, ^762^

Shi and colleagues^763^ found that exosomal circ0088300, originating from CAF cells, sponges miR‐1305, boosting GC cell growth, movement, and invasion. Xie's^614^ research uncovered that exosomal circSHKBP1 regulates the miR‐582‐3p/HUR/VEGF pathway, thereby advancing GC progression. This suggests circSHKBP1 as a promising diagnostic biomarker for GC.^614^ Highly expressed in GC, exosomal circ0044366 notably hinders the multiplication, migration, and tube formation of HUVECs by adjusting VEGF, thus aiding in GC progression.^764^ CircNHSL1, abundant in GC tissues, cells, and their exosomes, has been shown to reduce migration and invasion in GC cells. Its knockdown inhibits tumor growth, highlighting its potential as a diagnostic indicator for GC progression.^765^ CircNEK9 levels are elevated in GC tissues and cells. As reported by Yu et al.,^766^ the transfer of circNEK9 via exosomes hastens GC cell proliferation, migration, and invasion by targeting miR‐409‐3p.  CircNRIP1, also prevalent in GC, fuels disease advancement. Studies have shown that exosomal exchange facilitates the transmission of circNRIP1 between GC cells, augmenting progression and metastasis via the miR‐149‐5p/AKT1/mTOR pathway.^536^ Exosomes derived from GC can trigger the expression of circ_0004303, aiding the migration and homing of MSCs in neighboring tissues and modulating their biological functions, thereby promoting GC progression.^767^ Lu et al.^631^ demonstrated that circRanGAP1 is upregulated in GC tissues and serum‐derived exosomes, facilitating GC invasion and metastasis via the miR‐877‐3p/VEGFA axis. CircITCH, which is downregulated in GC cell lines, tissues, and serum‐derived exosomes, suppresses GC metastasis by sponging miR‐199a‐5p and increasing Klotho expression, serving as a potential biomarker for GC occurrence and development^768^ Last, Jiang et al.^769^ found that CDR1as plays a significant suppressive role in GC metastasis through the miR‐876‐5p/GNG7 axis.

The level of exosomal circSHKBP1 significantly decreased after gastrectomy, and increased expression of exosomal circSHKBP1 was associated with advanced TNM stages and poor survival.^614^ There was a statistically significant decrease in plasma exosomal circ0065149 levels in patients with early GC.^640^ This suggests that exosomal circSHKBP1 is a promising biomarker for GC prognosis.^640^ Circ0065149 levels are closely related to the prognosis and survival of patients with GC. Exosomal circ0065149 can be used as an early predictor of the prognosis of GC.

GC serum and exosomes from cisplatin‐resistant cells were upregulated with CircPVT1. CircPVT1 regulates autophagy, invasion and apoptosis through miR‐30a‐5p/YAP1 signaling.^770^ Circ0000260 expression was increased in cancer tissues and serum‐derived exosomes of GC patients, and knockdown of circ0000260 reduced resistance to cisplatin.^771^ GC cells resistant to oxaliplatin secreted exosomes expressing circ0032821.^772^ Exosomal circ0032821 targeted miR‐515‐5p/SOX9 pathways to facilitate cell resistance to oxaliplatin.^772^

ExocircRNAs were associated with advanced TNM stage, drug resistance, poor prognosis, and poor survival of GC, which can serve as stable biomarkers for GC resistance and survival. It is expected that exosome‐derived circRNAs will be effective markers for improving drug resistance and prognosis in clinical application once further research and improvements are made.

#### 4.3.8 ExocircRNAs and glioma

The most common CNS tumor, gliomas are characterized by rapid progression, aggressive growth, substantial heterogeneity, resistance to treatment, and poor prognosis.^773^, ^774^, ^775^ In the TME, exocircRNAs may play a crucial role in tumor progression.

Exosomal circATP8B4 can sequester miR‐766 and contribute to radioresistance in glioma patients undergoing radiotherapy.^776^ CircMMP1 can enhance tumor cell proliferation and inhibit apoptosis, with elevated levels of exosomal circMMP1 observed in gliomas, where it drives oncogenesis through the circMMP1/miR‐433/HMGB3 axis.^777^ This suggests that targeting this pathway may offer a viable treatment option for patients. In glioma cells and tissues, circHIPK3 promotes miR‐124 sequestration‐mediated enhancement of CCND2 expression, leading to an increased proliferation and invasion. Through the miR‐421/ZIC5 pathway, exosomal circHIPK3 can promote tumor cell proliferation and resistance to temozolomide (TMZ) treatment.^778^, ^779^ Additionally, TMZ‐resistant glioma patients have higher levels of exosomal circNFIX, which can promote oncogenic progression. CircNFIX upregulation reduces glioma cell sensitivity to RPN2 by inhibiting miR‐378e.^780^ TMZ‐resistant U251 cells can be sensitized to this drug by knocking down acetyl heparinase, which influences glioma cell TMZ resistance.^781^ In TMZ‐resistant glioma tissues and cells, circ_0072083 can regulate ALKBH5 via miR‐1252‐5p, thereby contributing to resistance to TMZ. TMZ‐resistant cells may benefit from the Warburg effect by releasing exosomal circ_0072083, which can enhance their TMZ‐resistance.^782^

A high level of serum exosomal circMMP1 (circ_0024108) has been associated with a poor prognosis for glioma patients.^777^ Exosomal circNFIX is associated with TMZ resistance, suggesting that analyses of this circRNA can improve patient care and therapeutic monitoring.^780^, ^783^ In glioblastoma cells, positive cell migration regulator splicing factor 1 (SRSF1) exhibits multiple binding sites for circSMARCA5. Additionally, SRSF3 is expressed at higher levels in glioma, and it may act as a positive regulator of SRSF1‐dependent glioma cell migration. By inhibiting the SRSF1/SRSF3/PTB axis, exosomal SMARCA5 can inhibit the migration of GBM cells, suggesting that it may function as a potent tumor suppressor in this type of cancer.^784^ The upregulation of circGLIS3 in high‐grade gliomas can also be facilitated by exosomes, which facilitate tumor invasion, angiogenesis, and phosphorylation of Ezrin (T567). Accordingly, high levels of p‐Ezrin (T567) are associated with high‐grade gliomas and poor outcomes.^785^

## 5 CONCLUSION AND PERSPECTIVES

This review examines the role of exocircRNAs in tumor cell proliferation, metastasis, drug resistance, and progression. Research predominantly focuses on tumor‐derived exocircRNAs, yet it is imperative to consider the complex and multifaceted TME, which involves various cell types. In future studies, exocircRNAs from CAF, tumor‐associated macrophages (TAM), and other immune cells should be investigated.

Research has demonstrated the importance of exocircRNAs in tumorigenesis, but many aspects remain unclear. Primarily synthesized and retained in the nucleus, the mechanisms governing the localization and sorting of circRNAs into exosomes are not comprehensively understood. Recent findings suggest that m6A modification may facilitate the cytoplasmic export of circRNAs,^786^ potentially influencing their incorporation into exosomes. Furthermore, the role of RBPs like argonaute and mannose‐binding lectin in binding circRNAs,^787^ and the dependence of miRNA sorting on the ESCRT complex, particularly Ago2,^788^ point toward a regulatory function of exosome‐associated RBPs in circRNA sorting. The mediation of exosome sorting by hnRNPA2B1 for specific circRNAs such as circ‐NEIL3 and circ‐CCAR1 has been noted.^702^, ^789^ Further research is required to delineate these regulatory mechanisms in detail.

Numerous studies suggest that exocircRNAs may be valuable biomarkers for cancer diagnosis and prognosis due to their highly conserved structures and tissue‐specific expression patterns. However, this potential necessitates additional experimental validation, larger cohort studies, and substantial theoretical backing to establish their clinical utility. Moreover, the field of engineered exosomes for targeted cancer therapy, although still nascent, shows promise. Future research should aim at identifying specific exocircRNAs and developing effective and safe engineered exosomes for clinical use.

In summary, our review provides an extensive exploration of the complex roles of exocircRNAs in cancer progression. It underscores the need for further investigation into exocircRNAs from various cellular sources within the TME and elucidates the regulatory mechanisms behind their exosomal sorting. Recognizing their potential in cancer diagnostics and prognostics, we advocate for more rigorous research to validate their clinical application as biomarkers.

## AUTHOR CONTRIBUTIONS

Yuanyong Wang, Yuchen Yang, and Rui Li were responsible for drafting the initial manuscript. Jin Zhang, Jie Li, Hui Zhu, Zhuofeng Liu, and Tian Li made substantial contributions to revising the initial draft, ensuring its academic rigor and clarity. Sijia Sun skillfully crafted the illustrations for the paper, enhancing its visual representation. Jin Zheng critically reviewed the manuscript, providing further refinements and insights. Litian Ma, as the corresponding author, carefully reviewed the final manuscript, ensuring that all aspects adhered to high academic standards, and subsequently gave his approval for its publication. All authors have read and approved the final manuscript.

## CONFLICT OF INTEREST STATEMENT

The authors declare no conflict of interest.

## ETHICS STATEMENT

Not applicable.

## Data Availability

Not applicable.
